# Global blue carbon accumulation in tidal wetlands increases with climate change

**DOI:** 10.1093/nsr/nwaa296

**Published:** 2020-12-15

**Authors:** Faming Wang, Christian J Sanders, Isaac R Santos, Jianwu Tang, Mark Schuerch, Matthew L Kirwan, Robert E Kopp, Kai Zhu, Xiuzhen Li, Jiacan Yuan, Wenzhi Liu, Zhi'an Li

**Affiliations:** Xiaoliang Research Station for Tropical Coastal Ecosystems, Key Laboratory of Vegetation Restoration and Management of Degraded Ecosystems, and the CAS Engineering Laboratory for Ecological Restoration of Island and Coastal Ecosystems, South China Botanical Garden, Chinese Academy of Sciences, Guangzhou 510650, China; Center of Plant Ecology, Core Botanical Gardens, Chinese Academy of Sciences, Guangzhou 510650, China; Southern Marine Science and Engineering Guangdong Laboratory (Guangzhou), Guangzhou 511458, China; State Key Laboratory of Estuarine and Coastal Research and Institute of Eco-Chongming, East China Normal University, Shanghai 201100, China; State Key Laboratory of Estuarine and Coastal Research and Institute of Eco-Chongming, East China Normal University, Shanghai 201100, China; National Marine Science Centre, School of Environment, Science and Engineering, Southern Cross University, Coffs Harbour NSW 2450, Australia; National Marine Science Centre, School of Environment, Science and Engineering, Southern Cross University, Coffs Harbour NSW 2450, Australia; Department of Marine Sciences, University of Gothenburg, Gothenburg 40530, Sweden; State Key Laboratory of Estuarine and Coastal Research and Institute of Eco-Chongming, East China Normal University, Shanghai 201100, China; Lincoln Centre for Water and Planetary Health, School of Geography, University of Lincoln, Lincoln LN67TS, UK; Virginia Institute of Marine Science, College of William and Mary, Gloucester Point, VA 23185, USA; Department of Earth and Planetary Sciences and Rutgers Institute of Earth, Ocean, and Atmospheric Sciences, Rutgers University, New Brunswick, NJ 08854, USA; Department of Environmental Studies, University of California, Santa Cruz, CA 95064, USA; State Key Laboratory of Estuarine and Coastal Research and Institute of Eco-Chongming, East China Normal University, Shanghai 201100, China; Department of Earth and Planetary Sciences and Rutgers Institute of Earth, Ocean, and Atmospheric Sciences, Rutgers University, New Brunswick, NJ 08854, USA; Department of Atmospheric and Oceanic Sciences, Fudan University, Shanghai 200433, China; Center of Plant Ecology, Core Botanical Gardens, Chinese Academy of Sciences, Guangzhou 510650, China; CAS Key Laboratory of Aquatic Botany and Watershed Ecology, Wuhan Botanical Garden, Chinese Academy of Sciences, Wuhan 430074, China; Xiaoliang Research Station for Tropical Coastal Ecosystems, Key Laboratory of Vegetation Restoration and Management of Degraded Ecosystems, and the CAS Engineering Laboratory for Ecological Restoration of Island and Coastal Ecosystems, South China Botanical Garden, Chinese Academy of Sciences, Guangzhou 510650, China; Center of Plant Ecology, Core Botanical Gardens, Chinese Academy of Sciences, Guangzhou 510650, China; Southern Marine Science and Engineering Guangdong Laboratory (Guangzhou), Guangzhou 511458, China

**Keywords:** coastal wetlands, blue C, C burial rate, global change

## Abstract

Coastal tidal wetlands produce and accumulate significant amounts of organic carbon (C) that help to mitigate climate change. However, previous data limitations have prevented a robust evaluation of the global rates and mechanisms driving C accumulation. Here, we go beyond recent soil C stock estimates to reveal global tidal wetland C accumulation and predict changes under relative sea level rise, temperature and precipitation. We use data from literature study sites and our new observations spanning wide latitudinal gradients and 20 countries. Globally, tidal wetlands accumulate 53.65 (95%CI: 48.52–59.01) Tg C yr^−1^, which is ∼30% of the organic C buried on the ocean floor. Modeling based on current climatic drivers and under projected emissions scenarios revealed a net increase in the global C accumulation by 2100. This rapid increase is driven by sea level rise in tidal marshes, and higher temperature and precipitation in mangroves. Countries with large areas of coastal wetlands, like Indonesia and Mexico, are more susceptible to tidal wetland C losses under climate change, while regions such as Australia, Brazil, the USA and China will experience a significant C accumulation increase under all projected scenarios.

## Teaser

The global tidal wetlands accumulate 53.65 (95%CI: 48.52-59.01) Tg C yr-1, which is ∼30% of the organic C buried on the ocean floor. Modelling based on current climatic drivers and under projected emissions scenarios revealed a net increase in the global C accumulation by 2100.

## INTRODUCTION

Mangroves and tidal marshes are highly productive wetlands that photosynthetically sequester atmospheric CO_2_ as organic carbon (C) [1]. A varying fraction of this C is buried in tidally inundated suboxic and anoxic sediments and thereby largely prevented from returning to the atmosphere [2]. Sediments within these wetlands do not become saturated with C, because sea level rise expands soil volume and creates accommodation space, accelerating burial of organic matter, and ultimately enhancing the long-term preservation of sedimentary C [3–5]. For a decade now, this coastal wetland C has been regarded as blue carbon (BC), to describe its disproportionately large contribution to global C sequestration [6,7]. The preservation and restoration of BC ecosystems such as mangroves and saltmarshes have been suggested as effective approaches to mitigating climate change [1]. Although the role of BC in climate change mitigation and adaptation has reached international prominence [7–9], several questions remain unanswered in BC studies [7]: what is the global extent and spatial distribution of coastal wetland BC? What factors influence the BC burial rates? How does climate change impact C accumulation in these BC ecosystems?

Feedback between vegetation and soil accretion help mangroves and tidal marshes to remain within the intertidal zone in the face of sea level rise [3,4] through the accumulation of mineral and organic sediments (i.e. the inland space available for sediments to accumulate and be colonized by wetland vegetation) [3,10,11]. However, human activities are imposing pressure on tidal wetlands, including climate-change-induced accelerated sea level rise, subsidence through groundwater as well as the extraction of other substances, and the reduction of sediment supply from the construction of systems that reduce sediment flow such as dams. Furthermore, a lack of accommodation space, which is the space made available for organic and inorganic material to deposit, in relation to an increase in relative sea level rise rate (RSLR), may limit the sediment accumulation rates in specific tidal wetlands [11,12]. As a result, there is growing concern that a lack of space for inland migration brought about through land use change or steep landward slopes may prevent the adaptation of mangrove and tidal marsh to fast rates of sea level rise [13,14].

BC accumulation in tidal wetlands is potentially driven by climatic factors (e.g. temperature, rainfall and evapotranspiration) [15–17], coastal oceanographic processes (e.g. tidal amplitude, currents and geomorphology) [13,14] and nutrient availability [18]. Identifying the controlling factors of C accumulation dynamics is critical for understanding the fate of the C buried by these wetlands under future climate change scenarios on global and regional scales. If climate change alters the fundamental drivers of organic C accumulation, the amount of sequestered C may also be modified and provide a different feedback to global warming.

**Figure 1.**
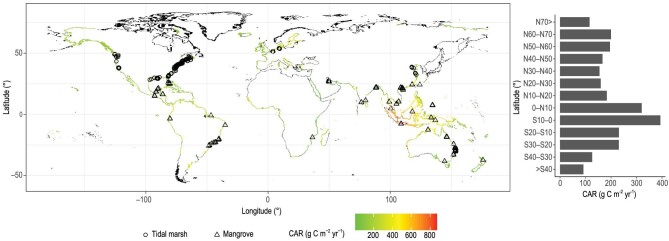
The spatial distribution of tidal wetlands and the observed C accumulation rate (CAR) points. The right panel indicates the arithmetic average CAR at every 10-degree band of latitude.

Here, we estimate global tidal wetland BC accumulation rates in different regions and countries. We rely on literature (n = 564) data supplemented by new (n = 49) soil core data from previously unaccounted-for areas in Indonesia, tropical South America and Africa (Fig. 1). Soil cores were dated with the radiometric geochronologies (^137^Cs, ^239+^^240^Pu and ^210^Pb) or Sediment Elevation Table (SET) methods covering timescales of sub-decade to decades. Both methods account for surface and subsurface processes, including organic and mineral sedimentation, sediment compaction and organic matter decomposition [19,20], and are widely used for sediment and carbon accretion rate measurements [21]. We first evaluate the spatial patterns and drivers of global soil accretion and C accumulation building on recent work focusing on C stocks [22,23]. We then predict C accumulation rates (CARs) in global tidal wetlands under differing emission scenarios based on Coupled Model Intercomparison Project Phase 5 (CMIP5) projections. We further combine projected CARs with a future global tidal wetland area change dataset [11] to develop the first projections of how global tidal wetland C accumulation will change in response to anthropogenic CO_2_ emissions. This approach focuses on a centennial timescale of anthropogenic climate change and prevents bias from other methods operating at different timescales [20]. Previous studies have shown that C accumulation increases with RSLR at specific locations [12,15], but there are no projections of how RSLR will drive global tidal wetland C accumulation.

## RESULTS AND DISCUSSION

### Global coastal C accumulation rates

The arithmetic average CARs were estimated to be 194 ± 15 g C m^−2^ y^−1^ for mangroves and 168 ± 7 g C m^−2^ yr^−1^ for tidal marshes, based on all compiled coastal wetland sites worldwide. The tidal marsh CAR in this study was much lower than the value in previous report by Ouyang and Lee [24], who estimated the global coastal marsh CAR to be 245 g C m^−2^ yr^−1^ based on data from 143 global sites. The mangrove CAR in our study was similar to a previous estimate of 163 g C m^−2^ yr^−1^ by Breithaupt *et al*. [2] based on 66 mangrove sites, but much lower than an earlier estimate (210 g C m^−2^ yr^−1^) by Chmura *et al.* [25], likely due to the increased dataset in more recent studies (Fig. 1).

**Figure 2.**
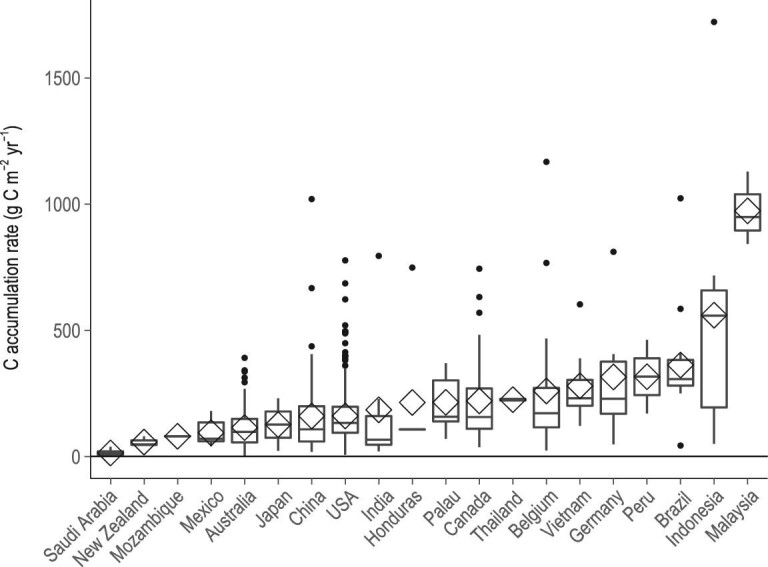
National C accumulation rates worldwide. The 20 countries listed here where observations are available account for 62% of global tidal wetland distribution. Diamond is the mean value, box is the interquartile range, error bar is the largest and smallest value within 1.5 times interquartile range above 75% and below 25%, respectively. Black points indicate outside values.

Tidal wetland CARs in the northern and southern hemispheres were not significantly different (p < 0.05). However, pooling the data into broad latitudinal bands revealed that tropical mangroves between 0° and 10° had the highest C accumulation per unit area (428 ± 7 g C m^−2^ yr^−1^ for the northern hemisphere and 384 ± 11 g C m^−2^ yr^−1^ for the southern hemisphere) and temperate tidal marshes between 30° and 40° had the lowest (144 ± 6 g C m^−2^ yr^−1^ for the northern hemisphere and 88.7 ± 3.5 g C m^−2^ yr^−1^ for the southern hemisphere) (Fig. 1). Mangroves located in the 0^o^ to 10^o^ latitudinal band, where ∼50% of mangrove living biomass occurs [26], were under-represented in previous global datasets [2,24,25]. Our new observations in Brazil, Indonesia and India help to fill this gap (Fig. 2). For example, Indonesian mangroves had the highest soil accretion rate (SAR, 36 ± 22 mm yr^−1^) and CAR (Fig. 2, 1722 ± 183 g C m^−2^ yr^−1^) [27].

### Drivers of global coastal C accumulation

A variety of environmental factors can drive wetland soil C sequestration [15–17]. We collected climatic and environmental factors for each site, including mean annual temperature (MAT), mean annual precipitation (MAP), tidal range, elevation, RSLR, total suspended matters (TSM) and tropical cyclone frequency, to attempt to discover the principal environmental factors that drive soil C accumulation in tidal marshes and mangrove forests (see details in Materials and Methods section). Our study contains a robust dataset to assess the main drivers of CARs on a global and local scale. We developed linear mixed-effect models (see Materials and Methods section and Table S1) that provide results for the detected main environmental factors on CARs (Table S2) as standardized coefficients (Fig. S1). These coefficients indicate the proportional change in CARs in response to one standard deviation change in an environmental factor. The model fit to observations is good (Fig. S2) and the residuals were normally distributed without obvious bias (Figs S3 and S4).

The linear mixed model using RSLR and MAT as covariate effects totally explained 51% of the variability in CAR in tidal marshes (Table S1), which is consistent with model and regional field evidence linking CAR with these environmental factors [12,15]. In mangroves, MAT and MAP explained 57% of the variation in CAR (Table S1, and the detailed model section listed in the Materials and Methods section). The positive relationship between mangrove CAR and temperature (Fig. S5) supports recent studies demonstrating how warming increases plant production, soil C stocks and soil surface elevation [5,16]. Precipitation is another important driver of soil C accumulation in warmer climate mangrove soils, but not in tidal marshes found in cooler regions (Fig. S5). This finding was also consistent with recent reports on how precipitation controls canopy height, aboveground biomass C and function of mangrove forests [30,31]. In mangrove soils, precipitation regulates organic C decomposition by modifying the oxygen supply to the soil [9,32,33] and increases plant productivity and growth by providing freshwater and nutrients [34]. Soil inundation following rainfall also increases mangrove belowground biomass [35]. Therefore, increasing precipitation in tropical coastal regions due to climate change [36] should also increase the C accumulation capacity in some of the world's largest mangrove systems including in the Indo-Pacific and tropical South American regions.

Local scale environmental factor like tidal range, marsh elevation and tropical cyclone frequency are also regarded as critical factors that affect the CAR in coastal wetlands. In this study, we extracted tidal range for each site from a recently developed global tidal range dataset [35,36]. However, the tidal range variables did not significantly affect the CAR in either tidal marshes or mangroves globally (Table S1 and Fig. S6).

Besides tidal ranges, elevation was also regarded as a local environmental factor directly related to the tidal wetland CAR. As a result of the apparent elevation distribution, compiled tidal marsh data can be further divided into high and low marsh in some sites. However, when we compared the CAR differences between upper tidal (high) marsh and lower tidal (low) marsh no significant difference was found (Fig. S7), which was similar to our previous observations in the USA [5]. Similarly, there were some mangrove sites that reported the relative location; we thus separated these sites into high and low tidal sites with apparent elevation difference. However, no significant difference between these tidal areas were observed (Fig. S7). As most of our compiled studies did not report site elevations, we further extracted the elevation data for each site from the CoastalDEM database, which is a digital terrain model providing bare earth elevations for coastal areas with 90 m horizontal resolution [37]. Again, we found that the elevation did not affect the CAR in either tidal marshes or mangroves (Fig. S6b).

As tropical cyclones have been reported to greatly affect the mangrove aboveground biomass and plant height [29], we also evaluated the tropical cyclone effect on mangrove CARs based on the Global Cyclone Hazard Frequency and Distribution, v1 (1980–2000) [38]. Our results (Table S1 and Fig. S6c) also suggested that the tropical cyclone frequency did not significantly affect mangrove CARs. Although higher tropical cyclone frequency was reported to damage the mangrove canopy height and reduce the aboveground biomass [29], the soil C accumulation in mangroves was a balance between biomass C inputs and soil C decomposition. It was therefore reasonable that tropical cyclone frequency has a limited influence on the mangrove soil CAR.

### Global extrapolation and projection

The global extrapolations were based on the 12 148 coastline segments from the Dynamic Interactive Vulnerability Assessment (DIVA) modeling framework [39], in which the existing coastal wetlands, as reported by the United Nations Environment Programme World Conservation Monitoring Center (UNEP WCMC), were combined [40]. Our global extrapolations show that tidal wetland accumulates 53.65 (95%CI: 48.52–59.01) Tg C yr^−1^ (Fig. 1 and Table 1), which is comparable to the carbon burial in global lakes [41], 30% of the carbon burial in the oceans [42] and 0.5% [43] of the current rate of anthropogenic carbon dioxide emissions. We further separated the data between vegetation types (Table S3), and found that tidal marsh contributed 12.63 Tg C yr^−1^. The value was comparable to previous reports for the global tidal marsh. For example, Ouyang and Lee [24] estimated ∼10.6 Tg C yr^−1^ sequestrated in global 41 657 km^2^ salt marsh, based on data from 143 sites. However, our global mangrove estimation (41 Tg C yr^−1^) was generally higher than previous estimates (18.4 to 34 Tg C yr^−1^) [25,44], likely because the new soil core data from tropical mangroves contain some of the highest mangrove CARs on the globe (Fig. 1). In terms of C sequestration per country, Indonesia was found to contain the greatest with 14.7 Tg C yr^−1^, and Australia the second greatest at 6.86 Tg C yr^−1^ (Table 1 and Table S3).

**Table 1.** Top ten countries in median annual tidal wetland C sequestration rate under current conditions and projected scenarios in 2100. Tidal wetland C sequestrations are displayed in the moderate-emission RCP4.5 and high-emission RCP8.5 scenarios under two assumed human activity scenarios as defined by Schuerch *et al.* [11]: inhibition of wetland inland migration in the regions with a population density of over 5 people km^−2^ and in the regions with a population density of over 300 people km^−2^.

Country	Current (C Tg)	RCP4.5 Pop. 300 (C Tg)	RCP8.5 Pop. 300 (C Tg)	RCP4.5 Pop. 5 (C Tg)	RCP8.5 Pop. 5 (C Tg)
Indonesia	14.7	19.22	19.10	15.06	10.35
Australia	6.86	12.71	26.63	12.25	25.29
USA	4.11	6.06	7.07	4.50	4.71
Brazil	3.26	4.24	5.51	3.83	4.36
Malaysia	2.01	3.54	3.28	3.23	2.56
Papua New Guinea	1.96	2.31	2.78	1.98	2.21
Mexico	1.56	1.85	7.43	0.97	0.73
Nigeria	1.33	1.03	0.61	0.99	0.15
China	1.24	1.87	3.64	1.82	3.45
Thailand	1.12	1.11	1.00	1.13	0.84
Others	15.45	17.66	22.95	13.04	12.85
**Total**	**53.65**	**71.6**	**100**	**58.8**	**67.5**

**Figure 3.**
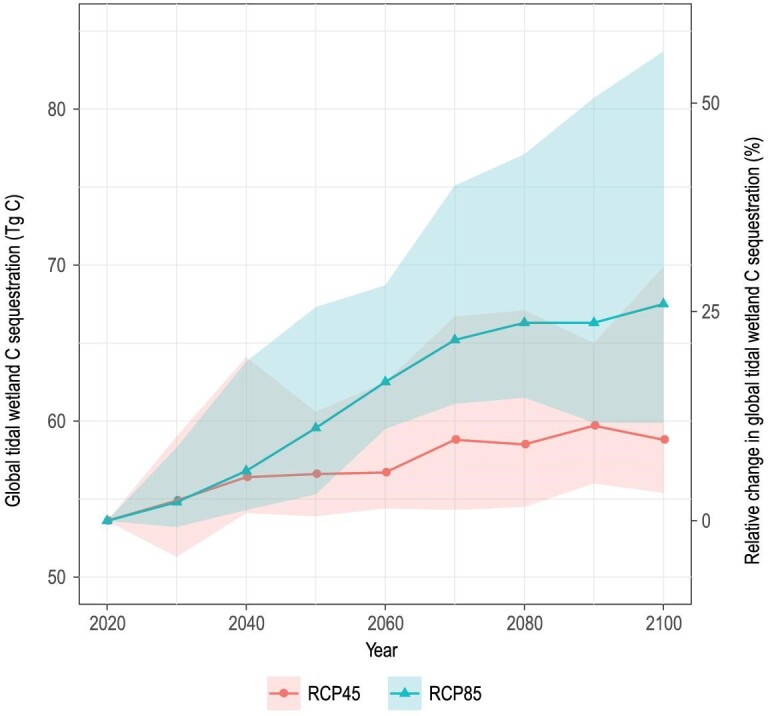
Projected global change in tidal wetland C sequestration. Results are displayed for moderate-emission scenario (RCP4.5) and high-emission scenario (RCP8.5) under the most restricted human adaptation scenario defined by Schuerch *et al.* [11] (i.e. population density threshold set as 5 people km^−2^; higher population density than this value would have no lateral accommodation space for tidal wetlands), giving the most conservative predicted increases in the global CARs found in this study. The colored shadings represent the 95% range (2.5%–97.5%) of the projected C distribution in global tidal wetlands. The solid lines denote the median of the distribution.

**Figure 4.**
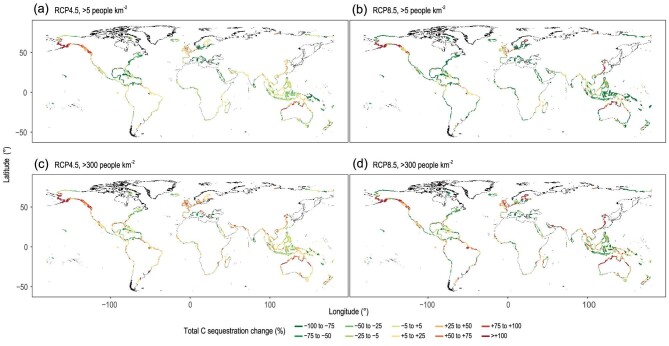
The projected 2100 spatial distribution of tidal wetland C sequestration changes. Relative changes in tidal wetland C sequestration are displayed for the RCP4.5 (a and c) and RCP8.5 (b and d) scenarios under two assumed human activity scenarios by Schuerch *et al.* [11]: inhibition of wetland inland migration in the regions with a population density of over 5 people km^−2^ (a and b) and over 300 people km^−2^ (c and d).

To estimate changes in global CAR under projected Representative Concentration Pathway (RCP)4.5 and RCP8.5 scenarios by 2100, we applied the linear changes in CAR that is a function of MAP, MAT and RSLR for tidal marshes and mangroves (Models 1 and 2, see details in Materials and Methods section). The coastal wetland area changes under corresponding scenarios were extracted from a recent integrated global model developed by Schuerch *et al.* [11]. The model considered both the ability of coastal wetlands to vertically build up through sediment accretion, and the accommodation space indicated by population density, namely, the vertical and lateral space available for fine sediments to accumulate and be colonized by wetland vegetation under different climate change scenarios [11]. We estimate that global tidal wetland C accumulation (Fig. 3) will increase by at least 35%, i.e. from 53.65 to 67.5 (95%CI: 60–83.7) Tg C yr^−1^ in 2100 under the high-emissions RCP8.5 scenario (Table 1), even though the total area will decrease by 30% as a result of highly limited accommodation space [11]. Under the moderate-emissions RCP4.5 scenario, total C accumulation will increase from 10% to 34% relative to current values, i.e. from 58.8 to 71.6 Tg C yr^−1^ in 2100, depending on the projected total wetland area regulated by population density [11] (Table 1 and Figs 3 and 4). We thus suggest that even with a decrease in wetland area, the total C accumulation in tidal wetlands will keep increasing until the end of the 21st century due to increased temperature and precipitation in mangroves, and higher RSLR in tidal marshes. Our estimated increases in soil C accumulation would have numerous implications for coastal C budgets and the assessment of their sink potential. Given increasing atmospheric C and global temperatures, our modeling implies that the soil organic C accumulation and wetland extent will be more resilient to sea level rise by 2100 than previously suggested [3,11].

At the national scale, Australia will have the greatest C sequestration in 2100, ranging from 12 to 27 Tg C yr^−1^ depending on scenario assumptions (Table 1 and Table S3). Our projections indicate that Indonesia, Malaysia and Mexico are vulnerable to climate change under the most limited lateral accommodation space [11], while countries such as Australia, USA, Brazil and China will have significant gains in tidal wetland C sequestration capacities under the RCP4.5 and RCP8.5 scenarios due to expanding wetland area [11,45].

Most of the C that accumulated in the tidal wetland sediments was derived from autochthonous organic matter, particularly root material [46,47]. However, the methods used in this study do not account for the C that accumulates along the active root zone. The changes in C attributed to root mass increases can be important to the ecosystem scale C stocks of coastal wetlands, which is outside the scope of the sediment dating techniques employed in this study [48,49]. Furthermore, much of the organic material produced in tidal wetlands can be exported to the ocean or buried in adjacent areas such as tidal flats [2] and the coastal ocean [49]. For instance, mudflats near tidal wetlands may have comparable sediment CARs for which tidal vegetated wetlands are often a major source of organic matter [2,50]. In addition, lateral exports of bicarbonate from tidal marsh [51] and mangrove [49] soils into the coastal ocean can exceed soil accumulation and represent an overlooked C sequestration mechanism on timescales of thousands of years. Under warming conditions, mangrove forests are migrating in a poleward direction, encroaching on tidal marshes and thereby increasing the C accumulation capacity of global tidal wetlands [17,52,53]. Therefore, our global C sequestration predictions are conservative since they do not include C that accumulates in active roots, exported to nearby ecosystems, and the increased C sequestration due to the ongoing poleward extension of mangroves.

Our global extrapolations and projections of literature and new data under RCP4.5 and RCP8.5 contextualize the empirical relationships between CAR and environment factors [54]. The projection of global tidal wetland C sequestration considered both wetland area changes [11] and the CAR response to future climate change factors (MAT, MAP and RSLR). Although the model we used for future tidal wetland areas is contested among researchers [11,36,55], the results of our global projection mainly rely on the increasing CAR under future climate change factors. For example, the global C sequestration is projected to increase by at least 35% towards the final decade of this century (Fig. 3) even though the total area of tidal wetlands will decrease by 30% as predicted by Schuerch *et al.* [11]. Moreover, the patterns of CAR in response to climate change that we observed in this study are in line with a previous modeling simulation of salt marsh C accumulation in response to climate change [15]. We thus believe that our study captures the actual CAR responses to altered RSLR, precipitation and temperature, which may benefit future process-based models.

## CONCLUSION

Overall, our results highlight the feedback between climate change and C sequestration in tidal wetlands. Projected increases in precipitation, temperature and RSLR will drive a net increase in global C sequestration in wetlands during the 21st century. The projected 59 to 100 Tg C yr^−1^ total global tidal wetland C accumulation in 2100 (Table 1) represents an additional sink of 5 to 46 Tg of atmospheric CO_2_-C yr^−1^ for a total of 53.65 Tg C yr^−1^. Even though these global tidal wetlands only occupy <0.1% of the global area, they could offset at least 0.5% of the current anthropogenic CO_2_ emission rates, a spatial efficiency that is 15 times higher than terrestrial ecosystems and 50 times higher than the open ocean per unit area [46]. Therefore, our results demonstrate that preserving and rehabilitating mangroves and salt marshes will remain an effective approach to tackling global climate change with significant regional benefits in tidal wetland-rich countries.

## MATERIALS AND METHODS

### Data sources

We used the Web of Science (Thomson Reuters, New York, NY) and Google Scholar (Google Inc., Mountain View, CA) to search the literature using the terms: (accretion)+(tidal wetlands or salt marsh or mangrove* or coastal wetland or coastal marsh)+(soil or sediments). To be included in our dataset, studies had to cover the temporal scale of sub-decade to decades, which are compatible with anthropogenic climate change and future CMIP5 projections. Data were extracted until September 2018 from published studies or obtained via personal communication. We compiled a total of 102 studies with 564 reported sites that matched our criteria (Table S2).

Besides the above-reported data, we also included 49 sites in our own survey, which contained some regions that had never been reported before, like Africa, and some poor data regions such as India, Brazil and Indonesia. The sediment core intervals were sealed and cooled for transport. In the laboratory, cores were sectioned at 2 cm intervals; samples with known volumes were weighed and freeze-dried. The dry bulk density was calculated by dividing the dry weight of the sediment by the initial volume. The total organic C was analyzed in a Flash Element Analyzer. For each of these study sites, we collected information on latitude, longitude, MAT, MAP, tidal range, mean tidal height and soil or sediment properties.

Soil C content data in some studies were derived from the measurement of loss on ignition (LOI). LOI measurement of mangrove soils was transformed into organic C content divided by 1.724 [56]. For tidal marsh soils, we applied the quadratic relationship specific to tidal marshes reported by Craft *et al.* [57]: TOC = 0.04 × LOI + 0.0025 × LOI^2^. Bulk density (BD) was also not reported in some sites. The missing BD was calculated based on a mixing model that describes the BD as a function of LOI in intertidal wetland sediments [58]. The model assumes that the bulk volume of sediment is equal to the sum of self-packing volumes of organic and mineral components or BD = 1/[LOI/k_1_ +(1−LOI)/k_2_], where k_1_ and k_2_ are the self-packing densities of the pure organic and inorganic components, respectively. The values of k_1_ and k_2_ were estimated to be 0.085 ± 0.0007 and 1.99 ± 0.028 g cm^−3^, respectively [58].

Some studies have directly reported the CARs, which were calculated by SAR multiplied by the soil C density. The vertical SAR represented average SARs from sub-decade to decades depending on the different dating methods. Where reports made available both SAR and C density, the CAR was calculated in this study. Some studies have reported the SAR and C density or C content for multiple layers, reflecting their changes over time. For these studies, we averaged their C density and SAR over up to 30 cm soils that recorded the most recent C accumulation (less than 100 yrs for most sites).

The MAT and MAP for each site were acquired from world climate data [59]. RSLR data were collected from the Permanent Service for Mean Sea Level (PSMSL) database [60]. TSM (mg/L) is derived from Medium Resolution Imaging Spectrometer (MERIS) satellite data, processed in the framework of the GlobColour project (http://globcolour.info) [11]. We used the monthly averages from April 2002 to April 2012 that have a horizontal resolution of 1/24°. We extracted the tidal range for each site from a newly developed global tidal range dataset [35,36], representing the tidal range (that is, the difference between mean low water and mean high water), mean high water neap (MHWN) and mean high water spring (MHWS) tidal levels. Most of the compiled studies did not report the site elevations, and we thus extracted the elevation data for each site from the CoastalDEM database, which is a digital terrain model providing bare earth elevations for coastal areas with 90 m horizontal resolution [37]. We assumed that all the tidal wetlands were located between the lowest and highest tidal levels, and removed the elevation data which were much higher or lower than the high and low tidal levels.

The cyclone dataset is a 2.5-minute global grid based on more than 1600 storm tracks from 1 January 1980 to 31 December 2000 for the Atlantic, Pacific and Indian Oceans that were assembled and modeled at UNEP/GRID-Geneva PreView [61]. The site-specific cyclone hazard risk was extracted from the dataset.

### Statistical analysis

Linear mixed models (LMMs) were used to evaluate the factors that may drive the measured CAR and SAR. The study reference was included as a random factor because clustering replicated by study location could introduce spatial autocorrelation. The LMMs were fit assuming a Gaussian error distribution using the ‘lme4’ package for the R statistical program [62]. We constructed the LMMs separately for tidal marsh and mangrove (Table S1). There were many missing elevation data in our dataset, and Figs S2 and S3 have shown that elevation has no effect on tidal wetland CAR in either tidal marshes or mangroves globally. As a result, we did not include elevation in our LMMs analysis. Initial tidal marsh LMMs included all of the putative explanatory variables (including vegetation types, longitude, latitude, MAT, MAP, RSLR, tidal range and TSM) to explain the variations in CAR (see Table S1), while the initial mangrove LMMs also have the tropical cyclone risk variable (see the details in Table S1). Given the positive skew in the distribution of CAR, it was log-transformed prior to use in the models. All the environmental variables (MAT, MAP, RSLR, tidal range, TSM, cyclone risk) were standardized (subtracting the mean and dividing by standard deviation). The standardization makes coefficients comparable among environmental factors, which we show in Fig. S3. Neither of these data transformations significantly altered the statistical outputs, so were retained in the final models.

Model selection was performed using the Akaike information criterion (AIC) of competing models [63,64]. In tidal marshes, RSLR and MAT were included in the most parsimonious final model (Model 1), while only MAT and MAP were included in the final mangrove model (Model 2). All reported P-values from LMMs are generated by the ‘r.squaredGLMM’ function from ‘MuMIn’ package. Variance explained by the model was estimated by calculating R^2^ values for the minimally adequate LMM following Nakagawa and Schielzeth to retain the random effects structure [64].

Tidal Marsh: log(CAR) ∼ RSLR + MAT+ (1|Studies)(Model 1)

Mangrove: log(CAR) ∼ MAP + MAT+ (1|Studies)(Model 2)

To assess model performance, we compared modeled vs. observed CAR data, summarized by goodness-of-fit measures. The modeled values were calculated as the expected (or fitted) CAR given environmental variables in the tidal marsh or mangrove datasets. These modeled values were then compared with the observed values. Figure S4 shows that the modeled and observed log-transformed CAR values are close to the 1 : 1 reference line. We then summarized their goodness of fit by Pearson correlation coefficient, which is a measure between −1 and +1, where a higher value indicates a better fit. For all vegetation groups, the modeled and observed CARs are highly correlated (R^2^ = 0.51 for the tidal marsh model, and 0.47 for the mangrove model). This assessment validates that the model can predict the data well.

To check model assumptions, we performed residual diagnostics. Residual diagnostics in Fig. S5 show that the residuals are not correlated with CAR values. Figure S6 validates the normality assumption of the model residuals, supporting log-transformed CAR variables.

We then used the final linear models without the random effects to evaluate the strength of the simple statistical relationship for global tidal marsh and mangrove CAR projections. In tidal marshes, the CAR was a linear function of RSLR and MAT (Model 1). However, the CAR generally reached a maximum rate if the SAR did not increase anymore. In our dataset, the SAR is a function of RSLR and TSM (Linear model: R^2^ = 0.20, p < 0.01, Model 3). We assumed that the TSM for each segment would not change until 2100 [11]. There will be a critical RSLR point for each segment when the SAR equals the RSLR in our future projection [11]. We thus assumed that the SAR would reach its maximum in the scenario where RSLR is higher than the critical RSLR point, and after this point the SAR would stop increasing with higher RSLR.

Tidal Marsh: SAR = 0.571 RSLR + 0.22 TSM + 1.62 (Model 3)

### Extrapolation and projection

The global extrapolation was based on the 12 148 coastline segments from the DIVA modeling framework [39]. Within each coastline segment, the existing coastal wetlands, as reported by the UNEP WCMC, were combined [40]. The geostatistical principle assumes that vegetation distribution gradually changes with environmental factors, like latitude, longitude, temperature and precipitation [65]. We thus assumed that C accumulation at each segment had the highest similarity to that at the closest sampled site. Spatial extrapolation methods thus were used in this study to calculate the CAR of each of the tidal wetland segments: the CAR of each tidal wetland segment was estimated based on the nearest five reported CAR sites. However, due to the rare sampling points in Africa, Europe and high latitude regions, we stress that there will be less accuracy for these regions in the extrapolation.

To estimate changes in global CAR under projected RCP4.5 and RCP8.5 scenarios by 2100, we applied the linear changes in CAR that are a function of MAP, MAT and RSLR for tidal marshes and mangroves. We used projected temperature and precipitation from the NASA Earth Exchange Global Daily Downscaled Projections (NEX-GDDP) dataset [66], a high-resolution (0.25° × 0.25°) dataset of global climate projections downscaled and bias-corrected from the output of 21 general circulation models (GCMs) in the CMIP5 archive [67] under the RCP 4.5 (represents a moderate-emission scenario in which global-averaged radiative forcing is around 4.5 W m^−2^ in 2100) and RCP8.5 (a comparatively high-emission scenario in which global-averaged radiative forcing is about 8.5 W m^−2^ in 2100) scenarios in the period between 2020 and 2100. However, the ensemble of the 21 realizations described above is an ‘ensemble of opportunity,’ as these GCMs are not independent from each other [68,69]. Thus, it fails to cover the full uncertainty of future projections and underestimates the extreme value in tails. To provide a probabilistic ensemble of climate projections and cover the tails of the probability distribution that are missing from the GCM ensemble, we use the surrogate/model mixed ensemble (SMME) method [70] to assign probabilistic weights to GCM output and generate surrogate models based on the probability distribution of global mean surface temperature (GMST) produced by a simple climate model. We first assign the weights by comparing the GMST from the 21 GCM outputs with the probability distribution of GMST produced by a simple climate model (e.g. MAGGIC6, Meinshausen *et al.* [71]). The number and weights of model surrogates are also determined in this step to cover the tails of distribution that are not captured by the 21 GCM outputs. We then generate model surrogates by scaling the GMSTs in the tail bins of the GMST probability distribution by the forced component from a selected GCM output, and adding the unforced component from the same GCM. Similarly to Carleton *et al*. [72], we generate 12 model surrogates. Together with the 21 GCM outputs, this approach provides us with 33 climate projections in total and 33 weights assigned to each projection.

We extracted the full distribution of future global RSLR data from Kopp *et al.*[ 73] at decadal intervals for locations of each coastal wetland segment under RCP4.5 and RCP8.5 trajectories. Site-to-site differences in the RSLR projections [73] originate from varying non-climatic background uplift or subsidence, oceanographic effects and spatially variable responses of the geoid and the lithosphere to shrinking land ice.

The projected CAR for tidal marshes and mangroves was thus calculated based on their (MAT, MAP and RSLR) differences between current values and future values by Model 1 and Model 2. The climate and RSLR projections captured physical uncertainty in the climate system. Besides the physical uncertainty, an important second source of uncertainty arises from the statistic estimates of the linear models (Model 1 and 2). To capture both sources of uncertainty, we firstly conduct a Monte Carlo procedure. For each of the coastal segments, we randomly draw a set of parameters that compose the CAR in the statistic models from empirical normal distributions defined by the means and confidence intervals. Secondly, we randomly sample paired MAT and MAP data from the 33 ensemble members under RCP4.5 and RCP8.5 scenarios, respectively. Each sampled projection is weighted by corresponding weight which is normalized by the sum of weights of all the samples. Thirdly, we randomly sample an RSLR projection from the full distribution of RSLR projections [41]. Fourthly, a projection of the CAR is estimated by combing the parameter sets with corresponding predictors for each equation (e.g. MAT and RSLR for Model 1 and MAT and MAP for Model 2). Finally, we repeat this process to obtain 1000 projection estimates for each equation. The uncertainty of CAR projections is estimated based on the 1000 projections at each coastal segment and each type of wetland. In this study, we reported their median value and the 95% (2.5%–97.5%) quantiles of the probability distribution.

The future coastal wetland area changes under RCP4.5 and RCP8.5 were extracted from an integrated global model that considers both the ability of coastal wetlands to build up vertically by sediment accretion, and the accommodation space which is driven by population density in each segment [11]. The projected global coastal wetland C accumulation amount was then calculated as the product of projected future CARs and coastal wetland areas for each coastline segment.

The data analysis, global extrapolation and projection were performed by R version 3.6 [74] and Matlab R2016a (The MathWorks, Inc. Natick, MA, USA).

## Data and materials availability

All the data used in this study are shown in results and supplementary information. The code will be available from FW on reasonable request.

## Supplementary Material

nwaa296_Supplemental_File

## References

[1] Duarte CM, Losada IJ, Hendriks IE et al. The role of coastal plant communities for climate change mitigation and adaptation. Nat Clim Chang 2013; 3: 961–8. 10.1038/nclimate1970

[2] Breithaupt JL, Smoak JM, Smith TJ et al. Organic carbon burial rates in mangrove sediments: strengthening the global budget. Glob Biogeochem Cycles 2012; 26: 2012GB004375. 10.1029/2012GB004375

[3] Kirwan ML, Megonigal JP. Tidal wetland stability in the face of human impacts and sea-level rise. Nature 2013; 504: 53–60. 10.1038/nature12856 24305148

[4] Mudd SM, D’Alpaos A, Morris JT. How does vegetation affect sedimentation on tidal marshes? Investigating particle capture and hydrodynamic controls on biologically mediated sedimentation. J Geophys Res Earth Surf 2010; 115: 2009JF001566. 10.1029/2009JF001566

[5] Wang F, Lu X, Sanders CJ et al. Tidal wetland resilience to sea level rise increases their carbon sequestration capacity in United States. Nat Commun 2019; 10: 5434. 10.1038/s41467-019-13294-z 31780651 PMC6883032

[6] McLeod E, Chmura GL, Bouillon S et al. A blueprint for blue carbon: toward an improved understanding of the role of vegetated coastal habitats in sequestering CO_2_. Front Ecol Environ 2011; 9: 552–60. 10.1890/110004

[7] Macreadie PI, Anton A, Raven JA et al. The future of Blue Carbon science. Nat Commun 2019; 10: 3998. 10.1038/s41467-019-11693-w 31488846 PMC6728345

[8] Zinke L. The colours of carbon. Nat Rev Earth Environ 2020; 1: 141. 10.1038/s43017-020-0037-y

[9] Spivak AC, Sanderman J, Bowen JL et al. Global-change controls on soil-carbon accumulation and loss in coastal vegetated ecosystems. Nat Geosci 2019; 12: 685–92. 10.1038/s41561-019-0435-2

[10] Schuerch M, Vafeidis A, Slawig T et al. Modeling the influence of changing storm patterns on the ability of a salt marsh to keep pace with sea level rise. J Geophys Res Earth Surf 2013; 118: 84–96. 10.1029/2012JF002471

[11] Schuerch M, Spencer T, Temmerman S et al. Future response of global coastal wetlands to sea-level rise. Nature 2018; 561: 231–4. 10.1038/s41586-018-0476-5 30209368

[12] Rogers K, Kelleway JJ, Saintilan N et al. Wetland carbon storage controlled by millennial-scale variation in relative sea-level rise. Nature 2019; 567: 91–5. 10.1038/s41586-019-0951-7 30842636

[13] Lovelock CE, Cahoon DR, Friess DA et al. The vulnerability of Indo-Pacific mangrove forests to sea-level rise. Nature 2015; 526: 559–63. 10.1038/nature15538 26466567

[14] Parkinson RW, Craft C, Delaune RD et al. Marsh vulnerability to sea-level rise. Nat Clim Chang 2017; 7: 756. 10.1038/nclimate3424

[15] Kirwan ML, Mudd SM. Response of saltmarsh carbon accumulation to climate change. Nature 2012; 489: 550–4. 10.1038/nature11440 23018965

[16] Osland MJ, Gabler CA, Grace JB et al. Climate and plant controls on soil organic matter in coastal wetlands. Glob Chang Bio 2018; 24: 5361–79. 10.1111/gcb.14376 29957880

[17] Coldren GA, Langley JA, Feller IC et al. Warming accelerates mangrove expansion and surface elevation gain in a subtropical wetland. J Ecol 2019; 107: 79–90. 10.1111/1365-2745.13049

[18] Lovelock CE, Feller IC, Reef R et al. Variable effects of nutrient enrichment on soil respiration in mangrove forests. Plant Soil 2014; 379: 135–48. 10.1007/s11104-014-2036-6

[19] Webb EL, Friess DA, Krauss KW et al. A global standard for monitoring coastal wetland vulnerability to accelerated sea-level rise. Nat Clim Chang 2013; 3: 458–65. 10.1038/nclimate1756

[20] Breithaupt JL, Smoak JM, Byrne RH et al. Avoiding timescale bias in assessments of coastal wetland vertical change. Limnol Oceanogr 2018; 63: S477–95. 10.1002/lno.10783 29937578 PMC5993342

[21] Howard J, Hoyt S, Isensee K et al. Coastal Blue Carbon: Methods for Assessing Carbon Stocks and Emissions Factors in Mangroves, Tidal Salt Marshes, and Seagrasses. Arlington, VA: Conservation International, Intergovernmental Oceanographic Commission of UNESCO, International Union for Conservation of Nature, 2014.

[22] Donato DC, Kauffman JB, Murdiyarso D et al. Mangroves among the most carbon-rich forests in the tropics. Nat Geosci 2011; 4: 293–7. 10.1038/ngeo1123

[23] Atwood TB, Connolly RM, Almahasheer H et al. Global patterns in mangrove soil carbon stocks and losses. Nat Clim Chang 2017; 7: 523–8. 10.1038/nclimate3326

[24] Ouyang X, Lee SY. Updated estimates of carbon accumulation rates in coastal marsh sediments. Biogeosciences 2014; 11: 5057–71. 10.5194/bg-11-5057-2014

[25] Chmura GL, Anisfeld SC, Cahoon DR et al. Global carbon sequestration in tidal, saline wetland soils. Glob Biogeochem Cycles 2003; 17: 1111. 10.1029/2002GB001917

[26] Giri C, Ochieng E, Tieszen LL et al. Status and distribution of mangrove forests of the world using earth observation satellite data. Glob Ecol Biogeogr 2011; 20: 154–9. 10.1111/j.1466-8238.2010.00584.x

[27] Kusumaningtyas MA, Hutahaean AA, Fischer HW et al. Variability in the organic carbon stocks, sources, and accumulation rates of Indonesian mangrove ecosystems. Estuar Coast Shelf Sci 2019; 218: 310–23. 10.1016/j.ecss.2018.12.007

[28] Osland MJ, Feher LC, Griffith KT et al. Climatic controls on the global distribution, abundance, and species richness of mangrove forests. Ecol Monogr 2017; 87: 341–59. 10.1002/ecm.1248

[29] Simard M, Fatoyinbo L, Smetanka C et al. Mangrove canopy height globally related to precipitation, temperature and cyclone frequency. Nat Geosci 2019; 12: 40–5. 10.1038/s41561-018-0279-1

[30] Davidson EA, Janssens IA. Temperature sensitivity of soil carbon decomposition and feedbacks to climate change. Nature 2006; 440: 165–73. 10.1038/nature04514 16525463

[31] Sanders CJ, Maher DT, Tait DR et al. Are global mangrove carbon stocks driven by rainfall? J Geophys Res Biogeosci 2016; 121: 2600–9. 10.1002/2016JG003510

[32] Ewel KC, Bourgeois JA, Cole TG et al. Variation in environmental characteristics and vegetation in high-rainfall mangrove forests, Kosrae, Micronesia. Glob Ecol Biogeogr Lett 1998; 7: 49–56. 10.2307/2997696

[33] Castañeda-Moya E, Twilley RR, Rivera-Monroy VH. Allocation of biomass and net primary productivity of mangrove forests along environmental gradients in the Florida Coastal Everglades, USA. Forest Ecol Manage 2013; 307: 226–41. 10.1016/j.foreco.2013.07.011

[34] Tan J, Jakob C, Rossow WB et al. Increases in tropical rainfall driven by changes in frequency of organized deep convection. Nature 2015; 519: 451–4. 10.1038/nature14339 25810207

[35] Pickering MD, Horsburgh KJ, Blundell JR et al. The impact of future sea-level rise on the global tides. Cont Shelf Res 2017; 142: 50–68. 10.1016/j.csr.2017.02.004

[36] Schuerch M, Spencer T, Temmerman S et al. Reply to ‘Global coastal wetland expansion under accelerated sea-level rise is unlikely’. EarthArXiv 2020; doi: 10.31223/osf.io/ycunb.

[37] Kulp SA, Strauss BH. New elevation data triple estimates of global vulnerability to sea-level rise and coastal flooding. Nat Commun 2019; 10: 4844. 10.1038/s41467-019-12808-z 31664024 PMC6820795

[38] Dilley M, Chen RS, Deichmann U et al. Natural Disaster Hotspots: A Global Risk Analysis. Washington, DC: World Bank, 2005.

[39] Spencer T, Schuerch M, Nicholls RJ et al. Global coastal wetland change under sea-level rise and related stresses: the DIVA Wetland Change Model. Glob Planet Chang 2016; 139: 15–30. 10.1016/j.gloplacha.2015.12.018

[40] McOwen CJ, Weatherdon LV, Bochove J-WV et al. A global map of saltmarshes. Biodivers Data J 2017; 5: e11764. 10.3897/BDJ.5.e11764 PMC5515097 28765720

[41] Mendonça R, Müller RA, Clow D et al. Organic carbon burial in global lakes and reservoirs. Nat Commun 2017; 8: 1694. 10.1038/s41467-017-01789-6 29162815 PMC5698497

[42] Duarte CM, Middelburg JJ, Caraco N. Major role of marine vegetation on the oceanic carbon cycle. Biogeosciences 2005; 2: 1–8. 10.5194/bg-2-1-2005

[43] Quéré C, Andrew R, Friedlingstein P et al. Global carbon budget 2018. Earth Syst Sci Data 2018; 10: 2141–94. 10.5194/essd-10-2141-2018

[44] Breithaupt JL, Smoak JM, Smith TJ et al. Organic carbon burial rates in mangrove sediments: strengthening the global budget. Glob Biogeochem Cycles 2012; 26: GB3011. 10.1029/2012GB004375

[45] IPCC, Pachauri RK, Meyer LA. Climate Change 2014: Synthesis Report. Contribution of Working Groups I, II and III to the Fifth Assessment Report of the Intergovernmental Panel on Climate Change. Geneva: IPCC, 2014.

[46] Ouyang X, Lee SY, Connolly RM. The role of root decomposition in global mangrove and saltmarsh carbon budgets. Earth-Sci Rev 2017; 166: 53–63. 10.1016/j.earscirev.2017.01.004

[47] Robertson AI, Aongi DM. Massive turnover rates of fine root detrital carbon in tropical Australian mangroves. Oecologia 2016; 180: 841–51. 10.1007/s00442-015-3506-0 26581419

[48] Lamont K, Saintilan N, Kelleway JJ et al. Thirty-year repeat measures of mangrove above- and below-ground biomass reveals unexpectedly high carbon sequestration. Ecosystems 2020; 23: 370–82. 10.1007/s10021-019-00408-3

[49] Santos IR, Maher DT, Larkin R et al. Carbon outwelling and outgassing vs. burial in an estuarine tidal creek surrounded by mangrove and saltmarsh wetlands. Limnol Oceanogr 2019; 64: 996–1013. 10.1002/lno.11090

[50] Pérez A, Libardoni BG, Sanders CJ. Factors influencing organic carbon accumulation in mangrove ecosystems. Biol Lett 2018; 14: 0237. 10.1098/rsbl.2018.0237 PMC6227860 30381450

[51] Wang ZA, Kroeger KD, Ganju NK et al. Intertidal salt marshes as an important source of inorganic carbon to the coastal ocean. Limnol Oceanogr 2016; 61: 1916–31. 10.1002/lno.10347

[52] Doughty CL, Langley JA, Walker WS et al. Mangrove range expansion rapidly increases coastal wetland carbon storage. Estuar Coasts 2015; 39: 385–96. 10.1007/s12237-015-9993-8

[53] Saintilan N, Wilson NC, Rogers K et al. Mangrove expansion and salt marsh decline at mangrove poleward limits. Glob Chang Biol 2014; 20: 147–57. 10.1111/gcb.12341 23907934

[54] Morris JT, Sundareshwar PV, Nietch CT et al. Responses of coastal wetlands to rising sea level. Ecology 2002; 83: 2869–77. 10.1890/0012-9658(2002)083[2869:ROCWTR]2.0.CO;2

[55] Törnqvist TrE, Cahoon DR, Day JW et al. Global coastal wetland expansion under accelerated sea-level rise is unlikely. EarthArXiv 2019; doi: 10.31223/osf.io/d2nhs.

[56] Allen SE, Grimshaw HM, Parkinson JA*et al.* Chemical Analysis of Ecological Materials. Oxford and London: Blackwell, 1974.

[57] Craft CB, Seneca ED, Broome SW. Loss on ignition and kjeldahl digestion for estimating organic carbon and total nitrogen in estuarine marsh soils: calibration with dry combustion. Estuaries 1991; 14: 175–9. 10.2307/1351691

[58] Morris JT, Barber DC, Callaway JC et al. Contributions of organic and inorganic matter to sediment volume and accretion in tidal wetlands at steady state. Earth Future 2016; 4: 110–21. 10.1002/2015EF000334 PMC5074445 27819012

[59] Fick SE, Hijmans RJ. WorldClim 2: new 1-km spatial resolution climate surfaces for global land areas. Int J Climatol 2017; 37: 4302–15. 10.1002/joc.5086

[60] Holgate SJ, Matthews A, Woodworth PL et al. New data systems and products at the permanent service for mean sea level. J Coast Res 2013; 29: 493–504.

[61] Center for Hazards and Risk Research Columbia University, Center for International Earth Science Information Network Columbia University and International Bank for Reconstruction and Development - The World Bank et al. Global Cyclone Hazard Frequency and Distribution. Palisades, NY: NASA Socioeconomic Data and Applications Center (SEDAC), 2005.

[62] Bates D, Mächler M, Bolker B et al. Fitting linear mixed-effects models using lme4. J Stat Softw 2015; doi:10.18637/jss.v067.i01. 10.18637/jss.v067.i01

[63] Zhu K, Chiariello NR, Tobeck T et al. Nonlinear, interacting responses to climate limit grassland production under global change. Proc Natl Acad Sci USA 2016; 113: 10589–94. 10.1073/pnas.1606734113 27601643 PMC5035850

[64] Nakagawa S, Schielzeth H. A general and simple method for obtaining R^2^ from generalized linear mixed-effects models. Methods Ecol Evol 2013; 4: 133–42. 10.1111/j.2041-210x.2012.00261.x

[65] Reich PB, Luo Y, Bradford JB et al. Temperature drives global patterns in forest biomass distribution in leaves, stems, and roots. Proc Natl Acad Sci USA 2014; 111: 13721–6. 10.1073/pnas.1216053111 25225412 PMC4183289

[66] Thrasher B, Maurer EP, McKellar C et al. Technical note: bias correcting climate model simulated daily temperature extremes with quantile mapping. Hydrol Earth Syst Sci 2012; 16: 3309–14. 10.5194/hess-16-3309-2012

[67] Taylor KE, Stouffer RJ, Meehl GA. An overview of CMIP5 and the experiment design. Bull Am Meteorol Soc 2012; 93: 485–98. 10.1175/BAMS-D-11-00094.1

[68] van Vuuren DP, Edmonds J, Kainuma M et al. The representative concentration pathways: an overview. Clim Chang 2011; 109: 5–31. 10.1007/s10584-011-0148-z

[69] Moss RH, Edmonds JA, Hibbard KA et al. The next generation of scenarios for climate change research and assessment. Nature 2010; 463: 747–56. 10.1038/nature08823 20148028

[70] Rasmussen DJ, Meinshausen M, Kopp RE. Probability-weighted ensembles of U.S. county-level climate projections for climate risk analysis. J Appl Meteorol Climatol 2016; 55: 2301–22. 10.1175/JAMC-D-15-0302.1

[71] Meinshausen M, Raper SCB, Wigley TML. Emulating coupled atmosphere-ocean and carbon cycle models with a simpler model, MAGICC6—Part 1: model description and calibration. Atmos Chem Phys 2011; 11: 1417–56. 10.5194/acp-11-1417-2011

[72] National Bureau of Economic Research. *Valuing the global mortality consequences of climate change accounting for adaptation costs and benefits*. https://www.nber.org/papers/w27599 (4 March 2020, date last accessed).

[73] Kopp RE, Horton RM, Little CM et al. Probabilistic 21st and 22nd century sea-level projections at a global network of tide-gauge sites. Earth Future 2014; 2: 383–406. 10.1002/2014EF000239

[74] R Core Team. R: A Language and Environment for Statistical Computing. Vienna: R Foundation for Statistical Computing, 2016.

