# Supplementary material for: Global blue carbon accumulation in tidal wetlands increases with climate change
Source: Natl Sci Rev. 2020 Dec 15;8(9):nwaa296. doi: 10.1093/nsr/nwaa296 (PMC8433083; doi:10.1093/nsr/nwaa296)
Supplement: nwaa296_Supplemental_File [file nwaa296_supplemental_file.docx]

Supplementary Materials

Global blue carbon accumulation in tidal wetlands increases with climate change

Faming Wang^1,2,3,4†*^, Christian J. Sanders^4,5†*^, Isaac R. Santos^5,6^, Jianwu Tang^4^, Mark Schurech^7^, Matthew L. Kirwan^8^, Robert E. Kopp^9^, Kai Zhu^10^, Xiuzhen Li^4^, Jiacan Yuan^9,11^, Wenzhi Liu^2,12^, Zhian Li^1,2,3^

**This PDF file includes:**

Figures. S1 to S7

Tables S1 to S3


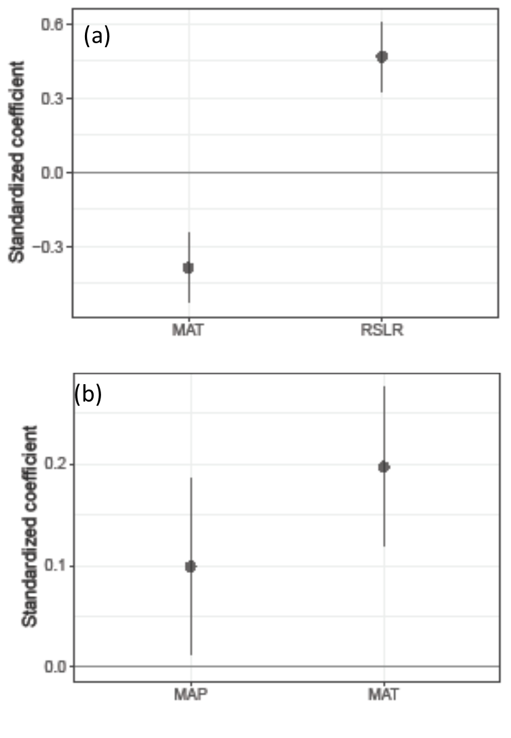


Fig. S1. Selected final model standardized coefficient and its variation. a. Tidal Marsh model: log(CAR)~MAT + RSLR +(1|Reference), with the data source reference as the random factor; b. Mangrove model: log(CAR)~ MAP+MAT+(1|Reference), with the data source reference as the random factor.


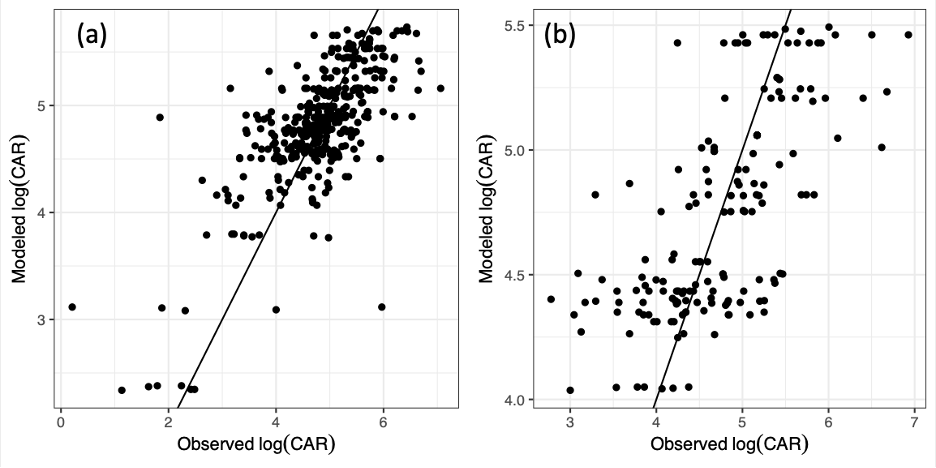


Fig. S2. Observed vs modeled log-transformed soil C accumulation rate in global tidal marsh (a) and mangrove sites (b). The solid line is 1:1 ratio line.


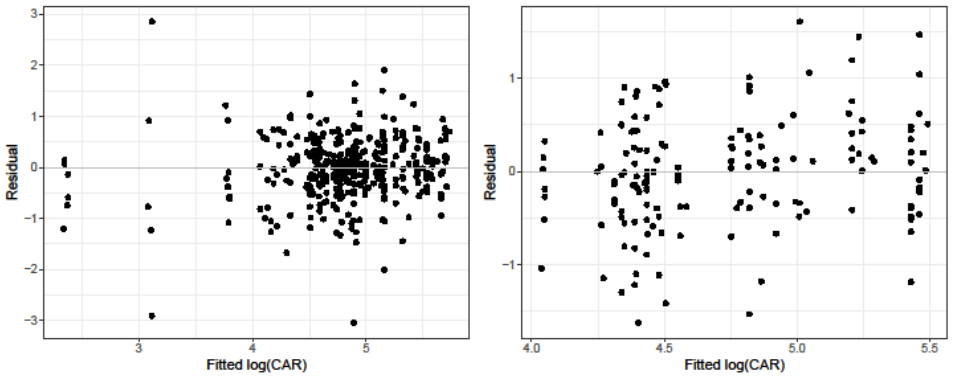


Fig.S3. The tidal marsh and mangrove final model residuals vs fitted CAR values. a. Tidal marsh model residual; b. Mangrove final model residual.


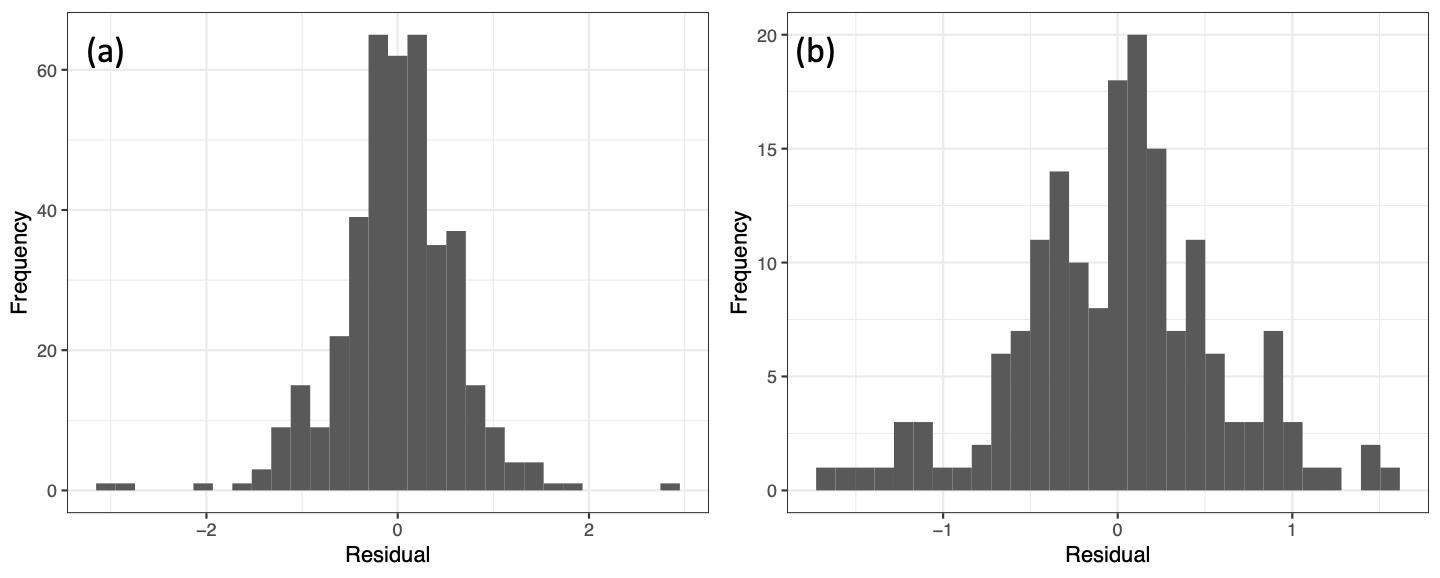


Fig.S4. The tidal marsh and mangrove final model residual distribution. a. Tidal marsh model residual; b. Mangrove final model residual.

Fig. S5.The relationship between C accumulation rate and global environmental factors (Temperature, Precipitation, and relative sea level rise rate (RSLR)) for tidal marsh and mangrove.

Fig. S6. The relationship between C accumulation rate and regional environmental factors (tidal range, wetland elevation, and tropical cyclone frequency) for tidal marsh and mangrove.

Fig.S7. The boxplot of tidal marsh and mangrove CAR rate in reported sites with different elevations and species compositions (Highmarsh vs Lowmarsh in tidal marsh sites, and Interior sites vs Margin sites in mangroves ).

Table S1.

The linear mixed models for the global tidal marsh and mangrove soil C accumulation rate (CAR).

|  | Models | Df | AIC |
| --- | --- | --- | --- |
|  | **Tidal Marshes** |  |  |
| Model 1 | log(CAR) ~ RSLR + MAP + MAT + TSM + mhws + mtidalrng + (1 \| References) | 9 | 911 |
| Model 2 | log(CAR) ~ RSLR + MAT + MAP + mtidalrng + mhws + (1 \| References) | 8 | 901 |
| Model 3 | log(CAR) ~ RSLR + mhws + MAT + mtidalrng + (1 \| References) | 7 | 885 |
| Model 4 | log(CAR) ~ RSLR + MAT + mtidalrng + (1 \| References) | 6 | 883 |
| Model 5 | log(CAR) ~ MAT + RSLR + (1 \| References) | 5 | 880 |
| Model 6 | log(CAR) ~ RSLR + (1 \| References) | 4 | 892 |
|  | **Mangroves** |  |  |
| Model 1 | log(CAR) ~ RSLR + MAT + MAP + TSM + mhws + mtidalrng + Cyclone+(1 \| References) | 10 | 368 |
| Model 2 | log(CAR) ~ MAP + MAT + RSLR +mhws+ mtidalrng + Cyclone+(1 \| References) | 9 | 367 |
| Model 3 | log(CAR) ~ MAP + MAT + RSLR +mhws+ mtidalrng + (1 \| References) | 8 | 366 |
| Model 4 | log(CAR) ~ MAT + MAP + mhws +mtidalrng + (1 \| References) | 7 | 366 |
| Model 5 | log(CAR) ~ MAP + MAT +mhws+ (1 \| References) | 5 | 367 |
| Model 6 | log(CAR) ~ MAP +MAT+ (1 \| References) | 4 | 366 |
| Model 7 | log(CAR) ~ MAP + (1 \| References) | 3 | 370 |

Note: CAR: Soil C accumulation rate, RSLR: Relative sea level rise rate, MAT: Mean annual temperature, MAP: mean annual precipitation, TSM: Total suspended matters, mhws: mean high spring water level, mtidalrng: mean tidal range, Cyclone: tropical cyclonge risk index.

Table S2.

The complied dataset and new dataset of tidal wetlands C accumulation rates.

| Lo | La | References | MAT | MAP | Vegetation | RSLR | CAR | TSM | mtidalrng | mhws | DEM Elevation | Cyclong frequency |
| --- | --- | --- | --- | --- | --- | --- | --- | --- | --- | --- | --- | --- |
| -81.08 | 25.36 | Breithaupt et al. 2014 | 23.60 | 1269.00 | Mangrove | 2.24 | 176.00 | 7.77 | 2.23 | 1.32 | 5.25 | 7.87 |
| -81.08 | 25.36 | Breithaupt et al. 2014 | 23.60 | 1269.00 | Mangrove | 2.24 | 176.00 | 7.77 | 2.23 | 1.32 | 5.25 | 7.87 |
| -81.08 | 25.36 | Breithaupt et al. 2014 | 23.60 | 1269.00 | Mangrove | 2.24 | 176.00 | 7.77 | 2.23 | 1.32 | 5.25 | 7.87 |
| -81.08 | 25.36 | Breithaupt et al. 2014 | 23.60 | 1269.00 | Mangrove | 2.24 | 176.00 | 7.77 | 2.23 | 1.32 | 5.25 | 7.87 |
| -81.08 | 25.36 | Breithaupt et al. 2014 | 23.60 | 1269.00 | Mangrove | 2.24 | 176.00 | 7.77 | 2.23 | 1.32 | 5.25 | 7.87 |
| -81.08 | 25.36 | Breithaupt et al. 2014 | 23.60 | 1269.00 | Mangrove | 2.24 | 176.00 | 7.77 | 2.23 | 1.32 | 5.25 | 7.87 |
| -81.73 | 26.02 | Cahoon and Lyach 1997 | 23.40 | 1306.00 | Mangrove | 2.30 | 328.00 | 4.30 | 0.94 | 0.71 | 1.77 | 6.58 |
| -81.74 | 26.03 | Cahoon and Lyach 1997 | 23.40 | 1306.00 | Mangrove | 2.30 | 291.00 | 4.30 | 0.94 | 0.71 | 0.00 | 6.55 |
| -81.75 | 26.02 | Cahoon and Lyach 1997 | 23.40 | 1306.00 | Mangrove | 2.30 | 191.00 | 4.30 | 0.94 | 0.71 | 1.76 | 6.54 |
| -81.73 | 26.02 | Cahoon and Lyach1997,1994 | 23.40 | 1306.00 | Mangrove | 2.30 | 228.00 | 4.30 | 0.94 | 0.71 | 1.77 | 6.58 |
| -80.60 | 25.00 | Callaway et al 1997 | 24.60 | 1159.00 | Mangrove | 4.20 | 129.60 | 12.66 | 0.54 | 0.37 | 0.00 | 7.92 |
| -80.30 | 25.30 | Callaway et al 1997 | 24.20 | 1344.00 | Mangrove | 4.20 | 165.80 | 4.26 | 0.57 | 0.34 | 1.74 | 7.78 |
| -80.70 | 24.90 | Callaway et al 1997 | 24.60 | 1147.00 | Mangrove | 1.90 | 120.00 | 7.39 | 0.57 | 0.37 | 4.06 | 8.02 |
| -80.60 | 25.00 | Callaway et al 1997 | 24.60 | 1159.00 | Mangrove | 1.90 | 57.70 | 12.66 | 0.54 | 0.37 | 0.00 | 7.92 |
| -80.55 | 24.99 | Callaway et al 1997 | 24.60 | 1159.00 | Mangrove | 3.90 | 152.20 | 9.16 | 0.57 | 0.37 | 3.07 | 7.90 |
| -81.70 | 26.00 | Lynch et al. 1989 | 23.40 | 1306.00 | Mangrove | 1.70 | 86.00 | 4.30 | 0.94 | 0.71 | 2.41 | 6.68 |
| -81.70 | 26.00 | Lynch et al. 1989 | 23.40 | 1306.00 | Mangrove | 1.40 | 90.00 | 4.30 | 0.94 | 0.71 | 2.07 | 6.68 |
| -81.70 | 26.00 | Lynch et al. 1989 | 23.40 | 1306.00 | Mangrove | 1.60 | 91.00 | 4.30 | 0.94 | 0.71 | 2.02 | 6.68 |
| -81.70 | 26.00 | Lynch et al. 1989 | 23.40 | 1306.00 | Mangrove | 1.70 | 99.00 | 4.30 | 0.94 | 0.71 | 2.53 | 6.69 |
| -81.78 | 26.08 | Marchio et al 2016 | 23.40 | 1306.00 | Mangrove | 2.40 | 21.00 | 4.59 | 0.92 | 0.70 | 1.07 | 6.36 |
| -81.78 | 26.10 | Marchio et al 2016 | 23.40 | 1306.00 | Mangrove | 2.40 | 47.00 | 4.59 | 0.92 | 0.70 | 4.89 | 6.29 |
| -81.78 | 26.10 | Marchio et al 2016 | 23.40 | 1306.00 | Mangrove | 2.40 | 74.00 | 4.59 | 0.92 | 0.70 | 2.45 | 6.30 |
| -81.78 | 26.10 | Marchio et al 2016 | 23.40 | 1306.00 | Mangrove | 2.40 | 126.00 | 4.59 | 0.92 | 0.70 | 3.19 | 6.30 |
| -81.77 | 26.09 | Marchio et al 2016 | 23.40 | 1306.00 | Mangrove | 2.40 | 50.00 | 4.59 | 0.92 | 0.70 | 2.32 | 6.34 |
| -81.78 | 26.09 | Marchio et al 2016 | 23.40 | 1306.00 | Mangrove | 2.40 | 127.00 | 4.59 | 0.92 | 0.70 | 0.00 | 6.32 |
| -81.78 | 26.09 | Marchio et al 2016 | 23.40 | 1306.00 | Mangrove | 2.40 | 162.00 | 4.59 | 0.92 | 0.70 | 1.32 | 6.34 |
| -81.00 | 25.20 | Smoak et al 2013 | 23.80 | 1255.00 | Mangrove | 2.50 | 448.76 | 7.77 | 2.23 | 1.32 | 1.00 | 8.01 |
| -81.10 | 25.40 | Smoak et al 2013 | 23.60 | 1269.00 | Mangrove | 3.60 | 100.00 | 7.77 | 2.23 | 1.32 | 2.67 | 7.83 |
| -35.10 | -8.70 | Sanders et al.2010a | 24.20 | 1738.00 | Mangrove | 5.00 | 410.00 | 4.46 | 1.71 | 1.16 | 3.88 | NA |
| -48.30 | -25.30 | Sanders et al. 2010c | 21.50 | 2241.00 | Mangrove | 2.50 | 317.00 | 3.83 | 1.49 | 1.18 |  | NA |
| -48.30 | -25.30 | Sanders et al. 2010c | 21.50 | 2241.00 | Mangrove | 2.90 | 337.00 | 3.83 | 1.49 | 1.18 |  | NA |
| 146.30 | -18.50 | Sanders et al. 2010b | 24.10 | 2135.00 | Mangrove | 1.48 | 336.00 | 3.54 | 2.28 | 1.89 | 0.00 | 6.80 |
| 146.30 | -18.50 | Sanders et al. 2010c | 24.10 | 2135.00 | Mangrove | 1.48 | 48.00 | 3.54 | 2.28 | 1.89 | 0.00 | 6.80 |
| -44.70 | -23.20 | Sanders et al. 2010c | 20.90 | 2014.00 | Mangrove | 4.00 | 393.00 | 1.15 | 1.12 | 0.93 | 0.00 | NA |
| -48.30 | -25.30 | Sanders et al. 2010a | 21.50 | 2241.00 | Mangrove | 3.90 | 362.00 | 3.83 | 1.49 | 1.18 |  | NA |
| -91.50 | 18.70 | Lynch 1989 | 26.40 | 1343.00 | Mangrove | 2.40 | 53.00 | 10.35 | 1.19 | 0.68 | 0.00 | 3.85 |
| -91.50 | 18.70 | Lynch 1989 | 26.40 | 1343.00 | Mangrove | 2.40 | 55.00 | 10.35 | 1.19 | 0.68 | 0.00 | 3.85 |
| -91.50 | 18.70 | Lynch 1989 | 26.40 | 1343.00 | Mangrove | 2.40 | 65.00 | 10.35 | 1.19 | 0.68 | 0.00 | 3.85 |
| -91.50 | 18.70 | Lynch 1989 | 26.40 | 1343.00 | Mangrove | 2.40 | 67.00 | 10.35 | 1.19 | 0.68 | 0.00 | 3.85 |
| -90.30 | 20.80 | Lynch 1989 | 26.50 | 869.00 | Mangrove | 3.00 | 70.00 | 10.27 | 0.54 | 0.36 | 2.61 | 6.62 |
| 110.57 | 19.99 | Zhang et al. 1996 | 24.10 | 1694.00 | Mangrove | 2.30 | 86.05 | 10.37 | 1.41 | 1.00 | 1.81 | 9.91 |
| 109.77 | 21.55 | Zhang et al. 1996 | 23.00 | 1470.00 | Mangrove | 2.30 | 76.85 | 9.68 | 2.64 | 1.92 | 1.87 | 8.89 |
| 109.77 | 21.55 | Zhang et al. 1996 | 23.00 | 1470.00 | Mangrove | 2.30 | 190.88 | 9.68 | 2.64 | 1.92 | 1.87 | 8.89 |
| 109.77 | 21.55 | Zhang et al. 1996 | 23.00 | 1470.00 | Mangrove | 2.30 | 34.71 | 9.68 | 2.64 | 1.92 | 1.87 | 8.89 |
| 109.77 | 21.55 | Zhang et al. 1996 | 23.00 | 1470.00 | Mangrove | 2.30 | 76.85 | 9.68 | 2.64 | 1.92 | 1.87 | 8.89 |
| 109.77 | 21.55 | Zhang et al. 1996 | 23.00 | 1470.00 | Mangrove | 2.30 | 44.62 | 9.68 | 2.64 | 1.92 | 1.87 | 8.89 |
| 146.30 | -18.50 | Alongi et al 1999 | 24.10 | 2135.00 | Mangrove | 1.80 | 67.00 | 3.54 | 2.28 | 1.89 | 0.00 | 6.80 |
| 99.16 | 10.28 | Alongi et al 2001 | 26.50 | 2034.00 | Mangrove | 0.08 | 228.00 | 6.81 | 1.29 | 0.84 | 2.10 | 1.00 |
| 99.16 | 10.28 | Alongi et al 2001 | 26.50 | 2034.00 | Mangrove | 0.08 | 228.00 | 6.81 | 1.29 | 0.84 | 2.10 | 1.00 |
| 99.21 | 10.37 | Alongi et al 2001 | 26.80 | 1942.00 | Mangrove | 0.08 | 222.00 | 6.81 | 1.29 | 0.84 | 2.57 | 1.00 |
| 99.21 | 10.37 | Alongi et al 2001 | 26.80 | 1942.00 | Mangrove | 0.08 | 222.00 | 6.81 | 1.29 | 0.84 | 2.57 | 1.00 |
| 146.15 | -18.36 | Brunskill et al 2002 | 23.20 | 1998.00 | Mangrove | 1.48 | 26.88 | 3.54 | 2.28 | 1.89 | 1.29 | 6.94 |
| 146.15 | -18.36 | Brunskill et al 2002 | 23.20 | 1998.00 | Mangrove | 1.48 | 174.72 | 3.54 | 2.28 | 1.89 | 1.29 | 6.94 |
| 146.14 | -18.37 | Brunskill et al 2002 | 23.20 | 1998.00 | Mangrove | 1.48 | 312.00 | 3.54 | 2.28 | 1.89 | 0.82 | 6.93 |
| 146.16 | -18.37 | Brunskill et al 2002 | 23.20 | 1998.00 | Mangrove | 1.48 | 84.00 | 3.54 | 2.28 | 1.89 | 0.59 | 6.93 |
| 146.16 | -18.39 | Brunskill et al 2002 | 23.20 | 1998.00 | Mangrove | 1.48 | 341.04 | 3.54 | 2.28 | 1.89 | 1.21 | 6.93 |
| 146.15 | -18.38 | Brunskill et al 2002 | 23.20 | 1998.00 | Mangrove | 1.48 | 294.00 | 3.54 | 2.28 | 1.89 | 1.07 | 6.93 |
| 146.15 | -18.38 | Brunskill et al 2002 | 23.20 | 1998.00 | Mangrove | 1.48 | 99.84 | 3.54 | 2.28 | 1.89 | 1.07 | 6.93 |
| 146.23 | -18.28 | Brunskill et al 2002 | 23.80 | 2397.00 | Mangrove | 1.48 | 154.56 | 4.26 | 2.18 | 1.81 | 3.16 | 6.99 |
| 146.23 | -18.27 | Brunskill et al 2002 | 23.80 | 2397.00 | Mangrove | 1.48 | 141.12 | 4.26 | 2.18 | 1.81 | 3.01 | 6.99 |
| 146.28 | -18.25 | Brunskill et al 2002 | 23.80 | 2397.00 | Mangrove | 1.48 | 70.56 | 4.26 | 2.18 | 1.81 | 0.87 | 6.99 |
| 146.23 | -18.25 | Brunskill et al 2002 | 23.80 | 2397.00 | Mangrove | 1.48 | 97.44 | 4.26 | 2.18 | 1.81 | 1.76 | 7.01 |
| -86.23 | 16.43 | Cahoon 2003 | 26.60 | 2275.00 | Mangrove | 1.50 | 106.91 | 0.36 | 0.21 | 0.14 | 3.23 | 2.85 |
| -86.23 | 16.43 | Cahoon 2003 | 26.60 | 2275.00 | Mangrove | 1.50 | 106.91 | 0.36 | 0.21 | 0.14 | 3.23 | 2.85 |
| -86.14 | 16.45 | Cahoon 2003 | 26.70 | 2332.00 | Mangrove | 1.50 | 748.40 | 0.36 | 0.21 | 0.14 |  | 2.89 |
| -86.14 | 16.45 | Cahoon 2003 | 26.70 | 2332.00 | Mangrove | 1.50 | 106.91 | 0.36 | 0.21 | 0.14 |  | 2.89 |
| -86.23 | 16.43 | Cahoon 2003 | 26.60 | 2275.00 | Mangrove | 1.50 | 106.91 | 0.36 | 0.21 | 0.14 | 3.23 | 2.85 |
| -86.23 | 16.43 | Cahoon 2003 | 26.60 | 2275.00 | Mangrove | 1.50 | 106.91 | 0.36 | 0.21 | 0.14 | 3.23 | 2.85 |
| -90.30 | 20.80 | Gonneea et al 2004 | 26.50 | 869.00 | Mangrove | 3.00 | 69.00 | 10.27 | 0.54 | 0.36 | 2.61 | 6.62 |
| -89.70 | 21.30 | Gonneea et al 2004 | 25.50 | 532.00 | Mangrove | 4.67 | 75.00 | 21.34 | 0.49 | 0.31 | 0.00 | 6.28 |
| -91.80 | 18.50 | Gonneea et al 2004 | 25.50 | 532.00 | Mangrove | 4.67 | 40.00 | 23.44 | 0.99 | 0.79 | 0.00 | 2.67 |
| 100.50 | 4.80 | Alongi et al 2004 | 27.40 | 2862.00 | Mangrove | 0.90 | 842.00 | 2.10 | 2.23 | 1.73 | 0.00 | NA |
| 100.50 | 4.80 | Alongi et al 2004 | 27.40 | 2862.00 | Mangrove | 0.90 | 949.00 | 2.10 | 2.23 | 1.73 | 0.00 | NA |
| 100.50 | 4.80 | Alongi et al 2004 | 27.40 | 2862.00 | Mangrove | 0.90 | 1129.00 | 2.10 | 2.23 | 1.73 | 0.00 | NA |
| 136.90 | -4.80 | Brunskill et al 2004 | 26.10 | 3369.00 | Mangrove | 3.48 | 558.00 | 15.10 | 1.85 | 1.42 |  | NA |
| 136.90 | -4.80 | Brunskill et al 2004 | 26.10 | 3369.00 | Mangrove | 3.48 | 412.00 | 15.10 | 1.85 | 1.42 |  | NA |
| 136.90 | -4.80 | Brunskill et al 2004 | 26.10 | 3369.00 | Mangrove | 3.48 | 637.00 | 15.10 | 1.85 | 1.42 |  | NA |
| 136.90 | -4.80 | Brunskill et al 2004 | 26.10 | 3369.00 | Mangrove | 3.48 | 717.00 | 15.10 | 1.85 | 1.42 |  | NA |
| -48.70 | -25.80 | Gonneea et al 2004 | 20.30 | 1937.00 | Mangrove | 5.60 | 296.00 | 3.66 | 1.37 | 1.11 |  | NA |
| 117.91 | 24.39 | Alongi et al 2005 | 21.40 | 1260.00 | Mangrove | 1.12 | 149.00 | 12.99 | 3.59 | 2.55 | 0.00 | 9.95 |
| 117.91 | 24.39 | Alongi et al 2005 | 21.40 | 1260.00 | Mangrove | 1.12 | 189.00 | 12.99 | 3.59 | 2.55 | 0.00 | 9.95 |
| 117.92 | 24.40 | Alongi et al 2005 | 21.40 | 1260.00 | Mangrove | 1.12 | 199.00 | 12.99 | 3.59 | 2.55 | 0.00 | 9.95 |
| 117.92 | 24.40 | Alongi et al 2005 | 21.40 | 1260.00 | Mangrove | 1.12 | 216.00 | 12.99 | 3.59 | 2.55 | 0.00 | 9.95 |
| 117.91 | 24.40 | Alongi et al 2005 | 21.40 | 1260.00 | Mangrove | 0.80 | 1020.00 | 12.99 | 3.59 | 2.55 | 0.00 | 9.94 |
| 117.91 | 24.40 | Alongi et al 2005 | 21.40 | 1260.00 | Mangrove | 0.80 | 667.00 | 12.99 | 3.59 | 2.55 | 0.00 | 9.94 |
| 124.20 | 24.30 | Tateda et al 2005 | 24.00 | 2085.00 | Mangrove | 2.18 | 22.00 | 1.22 | 1.14 | 0.92 | 0.00 | 10.00 |
| 124.20 | 24.30 | Tateda et al 2005 | 24.00 | 2085.00 | Mangrove | 2.18 | 230.00 | 1.22 | 1.14 | 0.92 | 0.00 | 10.00 |
| -92.76 | 15.13 | Adame et al 2005 | 26.70 | 3316.00 | Mangrove | 1.17 | 100.00 | 2.13 | 1.46 | 1.08 |  | 1.00 |
| -92.78 | 15.16 | Adame et al 2005 | 26.70 | 3316.00 | Mangrove | 1.17 | 140.00 | 2.13 | 1.46 | 1.08 |  | 1.00 |
| -92.81 | 15.18 | Adame et al 2005 | 27.40 | 2938.00 | Mangrove | 1.17 | 170.00 | 2.13 | 1.46 | 1.08 | 7.52 | 1.00 |
| -92.84 | 15.18 | Adame et al 2005 | 27.30 | 2659.00 | Mangrove | 1.17 | 180.00 | 2.13 | 1.46 | 1.08 | 3.50 | 1.00 |
| -92.85 | 15.18 | Adame et al 2005 | 27.30 | 2659.00 | Mangrove | 1.17 | 130.00 | 2.13 | 1.46 | 1.08 | 6.70 | 1.00 |
| -92.87 | 15.18 | Adame et al 2005 | 27.30 | 2659.00 | Mangrove | 1.17 | 150.00 | 2.13 | 1.46 | 1.08 | 9.29 | 1.00 |
| -92.83 | 15.17 | Adame et al 2005 | 27.40 | 2938.00 | Mangrove | 1.17 | 40.00 | 2.13 | 1.46 | 1.08 | 6.08 | 1.00 |
| -48.30 | -25.30 | Sanders et al. 2008 | 21.50 | 2241.00 | Mangrove | 1.80 | 296.00 | 3.83 | 1.49 | 1.18 |  | NA |
| 151.72 | -32.87 | Howe 2009 | 17.80 | 1119.00 | Mangrove | 0.65 | 105.00 | 2.05 | 1.11 | 0.92 | 0.10 | NA |
| 151.70 | -32.85 | Howe 2009 | 17.80 | 1119.00 | Mangrove | 0.65 | 89.00 | 2.05 | 1.11 | 0.92 | 0.80 | NA |
| 108.55 | 21.75 | Li et al 2010 | 22.80 | 2078.00 | Mangrove | 2.90 | 65.88 | 13.34 | 2.48 | 1.78 | 0.00 | 8.27 |
| 108.51 | 21.87 | Li et al 2010 | 22.70 | 2051.00 | Mangrove | 2.90 | 27.00 | 13.34 | 2.48 | 1.78 | 0.61 | 8.21 |
| 108.60 | 21.85 | Li et al 2010 | 22.70 | 2051.00 | Mangrove | 2.90 | 181.44 | 13.34 | 2.48 | 1.78 | 0.02 | 8.29 |
| 153.03 | -27.16 | Morelli 2012 | 20.20 | 1414.00 | Mangrove | 0.09 | 54.43 | 8.10 | 1.27 | 1.09 | 0.84 | 1.05 |
| 153.05 | -27.20 | Morelli 2012 | 20.30 | 1437.00 | Mangrove | 0.09 | 46.27 | 0.10 | 1.27 | 1.13 | 2.42 | 1.05 |
| 153.01 | -27.28 | Morelli 2012 | 20.30 | 1437.00 | Mangrove | 0.09 | 119.75 | 4.74 | 1.27 | 1.12 | 1.99 | 1.04 |
| 153.33 | -27.62 | Morelli 2012 | 19.90 | 1463.00 | Mangrove | 0.09 | 216.36 | 3.96 | 1.26 | 1.03 | 0.00 | 1.64 |
| 88.12 | 21.92 | Banerjee 2012 | 26.30 | 1549.00 | Mangrove | 1.45 | 20.09 | 25.00 | 2.58 | 1.97 | 0.53 | 5.00 |
| 88.47 | 21.78 | Banerjee 2012 | 26.20 | 1636.00 | Mangrove | 1.45 | 58.32 | 17.52 | 2.22 | 1.58 | 2.72 | 5.00 |
| 88.33 | 21.69 | Banerjee 2012 | 26.20 | 1651.00 | Mangrove | 1.45 | 66.30 | 21.07 | 2.14 | 1.63 | 1.19 | 5.00 |
| 88.77 | 22.08 | Banerjee 2012 | 26.20 | 1672.00 | Mangrove | 1.45 | 34.30 | 12.11 | 2.48 | 1.91 | 2.43 | 4.66 |
| 110.16 | 20.99 | Yang et al 2014 | 23.70 | 1244.00 | Mangrove | 2.30 | 292.23 | 11.31 | 2.16 | 2.11 | 2.26 | 9.35 |
| 109.76 | 21.57 | Yang et al 2014 | 23.00 | 1470.00 | Mangrove | 2.30 | 436.42 | 9.68 | 2.64 | 1.92 | 2.44 | 8.89 |
| 110.18 | 20.88 | Yang et al 2014 | 23.70 | 1302.00 | Mangrove | 2.30 | 243.97 | 11.31 | 2.16 | 2.11 | 0.75 | 9.43 |
| 110.42 | 21.16 | Yang et al 2014 | 23.60 | 1403.00 | Mangrove | 2.30 | 405.29 | 11.02 | 2.29 | 2.19 | 6.92 | 9.21 |
| -48.00 | -25.10 | Sanders et al. 2014 | 22.00 | 2412.00 | Mangrove | 3.20 | 250.00 | 3.03 | 1.53 | 1.23 | 0.00 | NA |
| -46.36 | -23.88 | Sanders et al. 2014 | 21.30 | 2671.00 | Mangrove | 3.20 | 1023.00 | 0.70 | 1.42 | 1.13 | 0.54 | NA |
| 108.58 | 21.85 | Xia et al 2015 | 22.70 | 2051.00 | Mangrove | 2.30 | 236.75 | 13.34 | 2.48 | 1.78 | 0.29 | 8.27 |
| 108.57 | 21.87 | Xia et al 2015 | 22.70 | 2051.00 | Mangrove | 2.30 | 118.37 | 13.34 | 2.48 | 1.78 | 0.00 | 8.26 |
| 108.60 | 21.86 | Xia et al 2015 | 22.70 | 2051.00 | Mangrove | 2.30 | 118.37 | 13.34 | 2.48 | 1.78 | 0.03 | 8.29 |
| 109.67 | 21.57 | Xia et al 2015 | 23.00 | 1470.00 | Mangrove | 2.30 | 43.25 | 9.90 | 2.62 | 1.87 | 1.47 | 8.87 |
| 109.76 | 21.49 | Xia et al 2015 | 23.00 | 1452.00 | Mangrove | 2.30 | 75.12 | 9.68 | 2.64 | 1.92 | 1.31 | 8.92 |
| 109.76 | 21.50 | Xia et al 2015 | 23.00 | 1452.00 | Mangrove | 2.30 | 70.57 | 9.68 | 2.64 | 1.92 | 2.44 | 8.92 |
| 109.76 | 21.50 | Xia et al 2015 | 23.00 | 1452.00 | Mangrove | 2.30 | 59.19 | 9.68 | 2.64 | 1.92 | 2.44 | 8.92 |
| 109.76 | 21.49 | Xia et al 2015 | 23.00 | 1452.00 | Mangrove | 2.30 | 50.08 | 9.68 | 2.64 | 1.92 | 1.44 | 8.92 |
| 153.41 | -27.52 | Lovelock et al 2015 | 19.90 | 1463.00 | Mangrove | 0.09 | 122.11 | 1.95 | 1.17 | 1.00 | 2.10 | 1.57 |
| 153.44 | -27.42 | Lovelock et al 2015 | 20.00 | 1472.00 | Mangrove | 0.09 | 103.90 | 1.95 | 1.17 | 1.00 |  | 1.50 |
| 153.44 | -27.44 | Lovelock et al 2015 | 20.00 | 1472.00 | Mangrove | 0.09 | 124.36 | 1.95 | 1.17 | 1.00 | 2.68 | 1.49 |
| 153.26 | -27.50 | Lovelock et al 2015 | 19.90 | 1314.00 | Mangrove | 0.09 | 94.86 | 3.83 | 1.26 | 1.04 | 2.31 | 1.53 |
| 153.10 | -27.35 | Lovelock et al 2015 | 20.20 | 1516.00 | Mangrove | 0.09 | 103.19 | 6.55 | 1.30 | 1.13 | 1.56 | 1.08 |
| 153.04 | -27.30 | Lovelock et al 2015 | 20.30 | 1437.00 | Mangrove | 0.09 | 16.07 | 6.55 | 1.30 | 1.13 | 4.94 | 1.04 |
| 153.39 | -27.78 | Sanders et al., 2016 | 20.20 | 1472.00 | Mangrove | 0.09 | 150.00 | 1.18 | 1.16 | 1.00 | 1.33 | 2.05 |
| 153.39 | -27.78 | Sanders et al., 2016 | 20.20 | 1472.00 | Mangrove | 0.09 | 150.00 | 1.18 | 1.16 | 1.00 | 1.02 | 2.05 |
| 108.50 | 21.81 | Xia et al 2016 | 22.80 | 2078.00 | Mangrove | 2.30 | 86.50 | 13.34 | 2.48 | 1.78 | 0.00 | 8.22 |
| 108.50 | 21.81 | Xia et al 2016 | 22.80 | 2078.00 | Mangrove | 2.30 | 186.67 | 13.34 | 2.48 | 1.78 | 0.00 | 8.22 |
| 108.57 | 21.87 | Meng et al 2016 | 22.70 | 2051.00 | Mangrove | 2.90 | 107.40 | 13.34 | 2.48 | 1.78 | 0.00 | 8.26 |
| 108.50 | 21.81 | Meng et al 2016 | 22.80 | 2078.00 | Mangrove | 2.90 | 22.80 | 13.34 | 2.48 | 1.78 | 0.00 | 8.22 |
| 106.89 | 10.53 | MacKenzie et al 2016 | 27.00 | 1757.00 | Mangrove | -1.25 | 227.60 | 14.97 | 2.94 | 1.92 | 0.57 | NA |
| 106.88 | 10.42 | MacKenzie et al 2016 | 26.90 | 1523.00 | Mangrove | -1.25 | 182.90 | 22.84 | 2.91 | 1.95 | 2.09 | NA |
| 105.11 | 8.65 | MacKenzie et al 2016 | 26.60 | 2320.00 | Mangrove | -1.25 | 388.60 | 28.71 | 2.32 | 1.25 | -2.00 | 1.00 |
| 105.11 | 8.65 | MacKenzie et al 2016 | 26.60 | 2320.00 | Mangrove | -1.25 | 274.50 | 28.71 | 2.32 | 1.25 | -2.00 | 1.00 |
| 105.11 | 8.65 | MacKenzie et al 2016 | 26.60 | 2320.00 | Mangrove | -1.25 | 602.70 | 28.71 | 2.32 | 1.25 | -2.00 | 1.00 |
| 105.11 | 8.65 | MacKenzie et al 2016 | 26.60 | 2320.00 | Mangrove | -1.25 | 233.70 | 28.71 | 2.32 | 1.25 | -2.00 | 1.00 |
| 105.08 | 8.63 | MacKenzie et al 2016 | 26.60 | 2320.00 | Mangrove | -1.25 | 121.00 | 28.71 | 2.32 | 1.25 | 0.97 | 1.00 |
| 105.08 | 8.63 | MacKenzie et al 2016 | 26.60 | 2320.00 | Mangrove | -1.25 | 206.80 | 28.71 | 2.32 | 1.25 | 0.97 | 1.00 |
| 134.62 | 7.63 | MacKenzie et al 2016 | 27.30 | 3482.00 | Mangrove | 1.73 | 322.30 | 2.90 | 1.11 | 0.83 | 1.83 | 1.85 |
| 134.62 | 7.63 | MacKenzie et al 2016 | 27.30 | 3482.00 | Mangrove | 1.73 | 369.70 | 2.90 | 1.11 | 0.83 | 1.83 | 1.85 |
| 134.54 | 7.58 | MacKenzie et al 2016 | 27.30 | 3482.00 | Mangrove | 1.73 | 280.60 | 2.90 | 1.11 | 0.83 | 3.47 | 1.85 |
| 134.54 | 7.58 | MacKenzie et al 2016 | 27.30 | 3482.00 | Mangrove | 1.73 | 153.80 | 2.90 | 1.11 | 0.83 | 3.47 | 1.85 |
| 134.58 | 7.39 | MacKenzie et al 2016 | 27.30 | 3482.00 | Mangrove | 1.73 | 69.80 | 1.57 | 1.11 | 0.84 | 3.44 | 1.85 |
| 134.58 | 7.39 | MacKenzie et al 2016 | 27.30 | 3482.00 | Mangrove | 1.73 | 157.50 | 1.57 | 1.11 | 0.84 | 3.44 | 1.85 |
| 134.51 | 7.41 | MacKenzie et al 2016 | 27.30 | 3482.00 | Mangrove | 1.73 | 141.00 | 1.57 | 1.11 | 0.84 | 2.31 | 1.85 |
| 134.51 | 7.41 | MacKenzie et al 2016 | 27.30 | 3482.00 | Mangrove | 1.73 | 136.40 | 1.57 | 1.11 | 0.84 | 2.31 | 1.85 |
| 134.63 | 7.58 | MacKenzie et al 2016 | 27.30 | 3482.00 | Mangrove | 1.73 | 251.90 | 2.90 | 1.11 | 0.83 | 9.11 | 1.85 |
| 134.51 | 7.38 | MacKenzie et al 2016 | 27.30 | 3482.00 | Mangrove | 1.73 | 356.70 | 1.57 | 1.11 | 0.84 | 3.40 | 1.85 |
| 134.51 | 7.38 | MacKenzie et al 2016 | 27.30 | 3482.00 | Mangrove | 1.73 | 119.50 | 1.57 | 1.11 | 0.84 | 3.40 | 1.85 |
| 109.75 | 21.57 | Zhu, et al 2016 | 23.00 | 1470.00 | Mangrove | 2.90 | 80.47 | 9.68 | 2.64 | 1.92 | 2.88 | 8.88 |
| 109.75 | 21.56 | Zhu, et al 2016 | 23.00 | 1470.00 | Mangrove | 2.90 | 105.42 | 9.68 | 2.64 | 1.92 | 1.45 | 8.88 |
| 109.75 | 21.56 | Zhu, et al 2016 | 23.00 | 1470.00 | Mangrove | 2.90 | 34.58 | 9.68 | 2.64 | 1.92 | 0.83 | 8.89 |
| 109.78 | 21.53 | Zhu, et al 2016 | 23.00 | 1470.00 | Mangrove | 2.90 | 83.97 | 9.68 | 2.64 | 1.92 | 0.80 | 8.90 |
| 109.78 | 21.53 | Zhu, et al 2016 | 23.00 | 1470.00 | Mangrove | 2.90 | 150.56 | 9.68 | 2.64 | 1.92 | 0.80 | 8.90 |
| 109.78 | 21.52 | Zhu, et al 2016 | 23.00 | 1470.00 | Mangrove | 2.90 | 69.09 | 9.68 | 2.64 | 1.92 | 0.68 | 8.91 |
| 175.86 | -37.20 | Perez et al 2017 | 14.20 | 2148.00 | Mangrove | 3.86 | 43.80 | 2.70 | 1.76 | 1.09 | 1.68 | 1.00 |
| 175.86 | -37.20 | Perez et al 2017 | 14.20 | 2148.00 | Mangrove | 3.86 | 47.50 | 2.70 | 1.76 | 1.09 | 2.47 | 1.00 |
| 175.86 | -37.20 | Perez et al 2017 | 14.20 | 2148.00 | Mangrove | 3.86 | 79.50 | 2.70 | 1.76 | 1.09 | 2.50 | 1.00 |
| 153.13 | -30.30 | Sanders own data | 18.30 | 1790.00 | Mangrove | 0.99 | 144.74 | 0.88 | 1.14 | 0.96 | 0.00 | 1.00 |
| 153.12 | -30.30 | Sanders own data | 18.30 | 1790.00 | Mangrove | 0.99 | 35.34 | 0.88 | 1.14 | 0.96 | 3.45 | 1.00 |
| 153.13 | -30.30 | Sanders own data | 18.30 | 1790.00 | Mangrove | 0.99 | 70.00 | 0.88 | 1.14 | 0.96 | 2.30 | 1.00 |
| 153.13 | -30.30 | Sanders own data | 18.30 | 1790.00 | Mangrove | 0.99 | 46.82 | 0.88 | 1.14 | 0.96 | 1.70 | 1.00 |
| 153.12 | -30.30 | Sanders own data | 18.30 | 1790.00 | Mangrove | 0.99 | 23.87 | 0.88 | 1.14 | 0.96 | 3.45 | 1.00 |
| 153.13 | -30.30 | Sanders own data | 18.30 | 1790.00 | Mangrove | 0.99 | 87.72 | 0.88 | 1.14 | 0.96 | 0.65 | 1.00 |
| 130.91 | -12.52 | Sanders own data | 27.40 | 1569.00 | Mangrove | 3.49 | 168.07 | 5.13 | 5.26 | 4.05 | 2.81 | 1.59 |
| 130.90 | -12.53 | Sanders own data | 27.40 | 1569.00 | Mangrove | 3.49 | 267.49 | 5.13 | 5.26 | 4.05 | 4.20 | 1.59 |
| 146.27 | -18.28 | Sanders own data | 23.80 | 2397.00 | Mangrove | 1.95 | 190.26 | 4.26 | 2.18 | 1.81 | 1.80 | 6.97 |
| 146.27 | -18.28 | Sanders own data | 23.80 | 2397.00 | Mangrove | 1.95 | 142.64 | 4.26 | 2.18 | 1.81 | 0.77 | 6.98 |
| 151.87 | -24.20 | Sanders own data | 22.10 | 1176.00 | Mangrove | 1.58 | 47.98 | 2.23 | 1.92 | 1.55 | 1.84 | 5.88 |
| 151.87 | -24.20 | Sanders own data | 22.10 | 1176.00 | Mangrove | 1.58 | 65.59 | 2.23 | 1.92 | 1.55 | 3.18 | 5.89 |
| 153.38 | -27.78 | Sanders own data | 20.20 | 1472.00 | Mangrove | 0.99 | 99.16 | 1.18 | 1.16 | 1.00 | 2.81 | 2.05 |
| 153.38 | -27.78 | Sanders own data | 20.20 | 1472.00 | Mangrove | 0.99 | 59.04 | 1.18 | 1.16 | 1.00 | 2.10 | 2.04 |
| 151.77 | -32.85 | Sanders own data | 17.80 | 1119.00 | Mangrove | 0.65 | 31.52 | 2.05 | 1.11 | 0.92 | 0.28 | NA |
| 151.76 | -32.84 | Sanders own data | 17.80 | 1119.00 | Mangrove | 0.65 | 69.29 | 2.05 | 1.11 | 0.92 | 0.43 | NA |
| 144.49 | -38.26 | Sanders own data | 14.30 | 589.00 | Mangrove | 0.65 | 66.67 | 1.34 | 1.01 | 0.92 | 0.60 | NA |
| 144.49 | -38.26 | Sanders own data | 14.30 | 589.00 | Mangrove | 0.65 | 20.73 | 1.34 | 1.01 | 0.92 | 0.40 | NA |
| 153.23 | -29.98 | Sanders own data | 19.00 | 1514.00 | Mangrove | 0.99 | 191.41 | 0.88 | 1.14 | 0.96 | 5.78 | 1.00 |
| 153.22 | -29.98 | Sanders own data | 19.00 | 1514.00 | Mangrove | 0.99 | 68.04 | 0.88 | 1.14 | 0.96 | 3.36 | 1.00 |
| 153.43 | -29.11 | Sanders own data | 19.40 | 1523.00 | Mangrove | 0.99 | 73.92 | 1.14 | 1.13 | 0.97 | 5.88 | 3.25 |
| 79.80 | 11.44 | Sanders own data | 28.40 | 1259.00 | Mangrove | 0.52 | 92.37 | 4.14 | 0.55 | 0.48 | 0.93 | 6.91 |
| 76.33 | 9.92 | Sanders own data | 27.30 | 3278.00 | Mangrove | 0.52 | 226.94 | 0.95 | 0.60 | 0.42 | 3.05 | 1.00 |
| 76.32 | 9.92 | Sanders own data | 27.30 | 3278.00 | Mangrove | 0.52 | 794.81 | 0.95 | 0.60 | 0.42 | 2.64 | 1.00 |
| -46.65 | -0.87 | Sanders own data | 26.30 | 2337.00 | Mangrove | 2.39 | 253.57 | 23.71 | 5.30 | 3.45 | 4.11 | NA |
| -43.03 | -22.72 | Sanders own data | 23.10 | 1322.00 | Mangrove | 2.39 | 276.16 | 6.23 | 1.03 | 0.88 | 1.72 | NA |
| -43.02 | -22.73 | Sanders own data | 23.10 | 1322.00 | Mangrove | 2.39 | 292.63 | 6.23 | 1.03 | 0.88 | 1.39 | NA |
| -40.31 | -20.24 | Sanders own data | 24.60 | 1090.00 | Mangrove | 2.39 | 379.08 | 6.30 | 1.29 | 0.97 | 1.11 | NA |
| -40.66 | -20.80 | Sanders own data | 24.60 | 1090.00 | Mangrove | 2.39 | 43.00 | 7.99 | 1.39 | 1.03 | 7.41 | NA |
| -40.17 | -19.94 | Sanders own data | 24.60 | 1137.00 | Mangrove | 2.39 | 281.95 | 7.52 | 1.29 | 0.97 | 1.43 | NA |
| -80.29 | -3.44 | Sanders own data | 25.30 | 287.00 | Mangrove | -1.22 | 170.13 | 2.29 | 2.04 | 1.42 | 1.02 | NA |
| -80.27 | -3.44 | Sanders own data | 25.30 | 287.00 | Mangrove | -1.22 | 461.97 | 2.29 | 2.04 | 1.42 | 4.65 | NA |
| 153.26 | -29.85 | Sanders own data | 19.00 | 1514.00 | Mangrove | 0.99 | 76.94 | 0.88 | 1.14 | 0.96 | 3.72 | 1.61 |
| 153.26 | -29.85 | Sanders own data | 19.00 | 1514.00 | Mangrove | 0.99 | 126.00 | 0.88 | 1.14 | 0.96 | 3.72 | 1.61 |
| -43.61 | -23.02 | Sanders own data | 23.20 | 1273.00 | Mangrove | 2.39 | 584.82 | 2.23 | 1.04 | 0.87 | 1.01 | NA |
| 36.34 | -18.76 | Sanders own data | 25.20 | 1168.00 | Mangrove | 0.66 | 79.87 | 15.64 | 2.67 | 1.93 | 1.11 | 8.98 |
| 153.20 | -30.14 | Sanders own data | 19.70 | 1753.00 | Mangrove | 0.99 | 180.54 | 0.88 | 1.14 | 0.96 | 4.10 | 1.00 |
| 153.20 | -30.14 | Sanders own data | 19.70 | 1753.00 | Mangrove | 0.99 | 213.86 | 0.88 | 1.14 | 0.96 | 3.44 | 1.00 |
| 153.59 | -28.88 | Sanders own data | 19.70 | 1753.00 | Mangrove | 0.99 | 29.07 | 1.14 | 1.13 | 0.97 | 0.00 | 3.07 |
| 108.81 | -7.67 | Kusumaningtyas et al 2019 | 26.50 | 3260.00 | Mangrove | 5.81 | 658.00 | 7.71 | 1.16 | 1.12 | 2.08 | NA |
| 109.00 | -7.67 | Kusumaningtyas et al 2019 | 26.50 | 3145.00 | Mangrove | 5.81 | 194.00 | 4.24 | 1.21 | 1.16 | -0.34 | NA |
| 49.57 | 27.29 | Saderne et al. 2018 | 25.00 | 94.00 | Mangrove | 1.25 | 8.70 | 10.55 | 1.07 | 0.89 | 0.00 | NA |
| 49.56 | 27.28 | Saderne et al. 2018 | 25.00 | 94.00 | Mangrove | 1.25 | 6.20 | 10.55 | 1.07 | 0.89 | 0.00 | NA |
| 49.57 | 27.28 | Saderne et al. 2018 | 25.00 | 94.00 | Mangrove | 1.25 | 23.00 | 10.55 | 1.07 | 0.89 | 0.00 | NA |
| 50.02 | 26.72 | Saderne et al. 2018 | 25.50 | 83.00 | Mangrove | 1.25 | 38.00 | 10.55 | 1.07 | 0.89 | 0.00 | NA |
| 50.01 | 26.66 | Saderne et al. 2018 | 25.50 | 83.00 | Mangrove | 1.25 | 22.00 | 10.55 | 1.07 | 0.89 | 0.00 | NA |
| 50.01 | 26.64 | Saderne et al. 2018 | 25.50 | 83.00 | Mangrove | 1.25 | 20.00 | 10.55 | 1.07 | 0.89 | 0.09 | NA |
| 50.01 | 26.64 | Saderne et al. 2018 | 25.50 | 83.00 | Mangrove | 1.25 | 12.00 | 10.55 | 1.07 | 0.89 | 0.01 | NA |
| 118.08 | 2.33 | Kusumaningtyas et al 2019 | 26.60 | 1988.15 | Mangrove | 3.42 | 1722.00 | 5.89 | 1.68 | 1.39 | 2.42 | NA |
| 133.48 | -2.51 | Sasmito 2020 | 26.93 | 2530.58 | Mangrove | 3.82 | 50.00 | 7.65 | 1.62 | 0.90 |  | NA |
| 133.51 | -2.58 | Sasmito 2020 | 26.90 | 2550.57 | Mangrove | 3.82 | 90.00 | 7.65 | 1.62 | 0.90 |  | NA |
| -72.80 | 41.26 | Anisfeld et al. 1999 | 10.40 | 1155.00 | Saltmarsh | 2.81 | 198.00 | 4.83 | 0.79 | 0.59 | 1.59 | 7.38 |
| -72.81 | 41.26 | Anisfeld et al. 1999 | 10.40 | 1155.00 | Saltmarsh | 2.81 | 75.00 | 4.83 | 0.79 | 0.59 | 0.31 | 7.37 |
| -72.66 | 41.27 | Anisfeld et al. 1999 | 10.30 | 1182.00 | Saltmarsh | 2.81 | 195.00 | 6.70 | 0.78 | 0.59 | 0.00 | 7.53 |
| -72.67 | 41.27 | Anisfeld et al. 1999 | 10.40 | 1155.00 | Saltmarsh | 2.81 | 192.00 | 6.70 | 0.78 | 0.59 | 0.00 | 7.51 |
| -72.67 | 41.25 | Anisfeld et al. 1999 | 10.40 | 1155.00 | Saltmarsh | 2.81 | 192.00 | 6.70 | 0.78 | 0.59 | 0.00 | 7.53 |
| -72.66 | 41.25 | Anisfeld et al. 1999 | 10.30 | 1182.00 | Saltmarsh | 2.81 | 154.00 | 6.70 | 0.78 | 0.59 | 0.00 | 7.54 |
| -72.68 | 41.25 | Anisfeld et al. 1999 | 10.40 | 1155.00 | Saltmarsh | 2.81 | 123.20 | 6.70 | 0.78 | 0.59 | 0.00 | 7.52 |
| -72.67 | 41.26 | Anisfeld et al. 1999 | 10.40 | 1155.00 | Saltmarsh | 2.81 | 220.00 | 6.70 | 0.78 | 0.59 | 0.00 | 7.53 |
| -72.67 | 41.26 | Anisfeld et al. 1999 | 10.40 | 1155.00 | Saltmarsh | 2.81 | 72.00 | 6.70 | 0.78 | 0.59 | 0.00 | 7.53 |
| -72.66 | 41.27 | Anisfeld et al. 1999 | 10.30 | 1182.00 | Saltmarsh | 2.81 | 105.00 | 6.70 | 0.78 | 0.59 | 0.00 | 7.53 |
| -72.67 | 41.27 | Anisfeld et al. 1999 | 10.40 | 1155.00 | Saltmarsh | 2.81 | 64.00 | 6.70 | 0.78 | 0.59 | 0.00 | 7.51 |
| -72.80 | 41.25 | Anisfeld et al. 1999 | 10.40 | 1155.00 | Saltmarsh | 2.81 | 112.50 | 4.83 | 0.79 | 0.59 | 0.00 | 7.39 |
| -72.80 | 41.25 | Anisfeld et al. 1999 | 10.40 | 1155.00 | Saltmarsh | 2.81 | 31.20 | 4.83 | 0.79 | 0.59 | 0.00 | 7.39 |
| -72.81 | 41.25 | Anisfeld et al. 1999 | 10.40 | 1155.00 | Saltmarsh | 2.81 | 84.00 | 4.83 | 0.79 | 0.59 | 1.58 | 7.38 |
| -73.14 | 40.96 | Armentano and Woodwell 1975 | 10.90 | 1159.00 | Saltmarsh | 2.44 | 216.13 | 2.30 | 0.91 | 0.69 | 0.12 | 7.26 |
| -73.14 | 40.96 | Armentano and Woodwell 1975 | 10.90 | 1159.00 | Saltmarsh | 2.44 | 196.00 | 2.30 | 0.91 | 0.69 | 0.12 | 7.26 |
| -73.14 | 40.96 | Armentano and Woodwell 1975 | 10.90 | 1159.00 | Saltmarsh | 2.44 | 146.00 | 2.30 | 0.91 | 0.69 | 0.12 | 7.26 |
| -73.14 | 40.96 | Armentano and Woodwell 1975 | 10.90 | 1159.00 | Saltmarsh | 2.44 | 196.86 | 2.30 | 0.91 | 0.69 | 0.12 | 7.26 |
| -74.05 | 40.81 | Artigas et al 2015 | 11.90 | 1163.00 | Saltmarsh | 2.84 | 192.00 | 8.67 | 1.10 | 0.80 | 0.00 | 6.06 |
| -75.27 | 38.87 | Boyd et al. 2016 | 12.80 | 1090.00 | Saltmarsh | 3.40 | 31.24 | 17.32 | 1.15 | 0.80 | -1.00 | 6.88 |
| -75.26 | 38.87 | Boyd et al. 2016 | 12.80 | 1090.00 | Saltmarsh | 3.40 | 157.00 | 17.32 | 1.15 | 0.80 | 0.00 | 6.89 |
| -75.26 | 38.86 | Boyd et al. 2016 | 12.80 | 1090.00 | Saltmarsh | 3.40 | 38.11 | 17.32 | 1.15 | 0.80 | -1.00 | 6.90 |
| -75.25 | 38.85 | Boyd et al. 2016 | 12.80 | 1090.00 | Saltmarsh | 3.40 | 169.00 | 17.32 | 1.15 | 0.80 | 0.46 | 6.93 |
| -75.26 | 38.83 | Boyd et al. 2016 | 12.80 | 1090.00 | Saltmarsh | 3.40 | 168.00 | 17.32 | 1.15 | 0.80 | 0.99 | 6.93 |
| -75.23 | 38.83 | Boyd et al. 2016 | 12.90 | 1097.00 | Saltmarsh | 3.40 | 177.00 | 17.32 | 1.15 | 0.80 | 0.59 | 6.96 |
| -75.21 | 38.81 | Boyd et al. 2016 | 12.90 | 1097.00 | Saltmarsh | 3.40 | 180.00 | 17.32 | 1.15 | 0.80 | 0.88 | 6.98 |
| -75.21 | 38.81 | Boyd et al. 2016 | 12.90 | 1097.00 | Saltmarsh | 3.40 | 187.26 | 17.32 | 1.15 | 0.80 | 0.64 | 6.99 |
| -75.20 | 38.81 | Boyd et al. 2016 | 12.90 | 1097.00 | Saltmarsh | 3.40 | 186.00 | 17.32 | 1.15 | 0.80 | 0.36 | 7.01 |
| -75.30 | 38.90 | Boyd et al. 2016 | 12.80 | 1090.00 | Saltmarsh | 3.40 | 224.50 | 17.32 | 1.15 | 0.80 | -1.00 | 6.82 |
| -75.30 | 38.89 | Boyd et al. 2016 | 12.80 | 1090.00 | Saltmarsh | 3.40 | 199.50 | 17.32 | 1.15 | 0.80 | 0.47 | 6.85 |
| -75.28 | 38.88 | Boyd et al. 2016 | 12.80 | 1090.00 | Saltmarsh | 3.40 | 148.00 | 17.32 | 1.15 | 0.80 | -1.00 | 6.87 |
| -71.39 | 41.82 | Bricker-Urso et al. 1989 | 9.70 | 1192.00 | Saltmarsh | 2.60 | 203.00 | 1.40 | 0.62 | 0.47 |  | 7.83 |
| -71.39 | 41.77 | Bricker-Urso et al. 1989 | 9.70 | 1192.00 | Saltmarsh | 2.60 | 495.00 | 1.40 | 0.62 | 0.47 |  | 7.85 |
| -71.38 | 41.74 | Bricker-Urso et al. 1989 | 9.70 | 1192.00 | Saltmarsh | 2.60 | 226.00 | 1.40 | 0.62 | 0.47 |  | 7.87 |
| -71.37 | 41.51 | Bricker-Urso et al. 1989 | 9.90 | 1177.00 | Saltmarsh | 2.60 | 234.00 | 0.79 | 0.65 | 0.47 | 1.81 | 7.95 |
| -71.69 | 41.36 | Bricker-Urso et al. 1989 | 9.80 | 1184.00 | Saltmarsh | 2.60 | 192.00 | 2.15 | 0.74 | 0.53 | 2.65 | 7.95 |
| -71.39 | 41.77 | Bricker-Urso et al. 1989 | 9.70 | 1192.00 | Saltmarsh | 2.60 | 209.00 | 1.40 | 0.62 | 0.47 |  | 7.85 |
| -71.34 | 41.65 | Bricker-Urso et al. 1989 | 9.90 | 1177.00 | Saltmarsh | 2.60 | 237.00 | 0.96 | 0.60 | 0.47 | 2.75 | 7.91 |
| -71.34 | 41.65 | Bricker-Urso et al. 1989 | 9.90 | 1177.00 | Saltmarsh | 2.60 | 490.50 | 0.96 | 0.60 | 0.47 | 2.75 | 7.91 |
| -96.79 | 28.32 | Callaway et al 1997 | 21.40 | 974.00 | Saltmarsh | 3.10 | 221.76 | 19.98 | 0.43 | 0.27 | 2.87 | 3.07 |
| -88.77 | 30.38 | Callaway et al 1997 | 19.60 | 1616.00 | Saltmarsh | 1.50 | 175.56 | 9.19 | 0.43 | 0.30 | 1.29 | 7.59 |
| -95.50 | 28.84 | Callaway et al 1997 | 20.70 | 1320.00 | Saltmarsh | 6.30 | 253.89 | 14.21 | 0.57 | 0.30 | 0.52 | 3.08 |
| -121.86 | 38.04 | Callaway et al. 2012 | 15.30 | 425.00 | Saltmarsh | 1.96 | 150.20 | 5.55 | 1.53 | 1.30 | 0.59 | NA |
| -121.86 | 38.04 | Callaway et al. 2012 | 15.30 | 425.00 | Saltmarsh | 1.96 | 158.40 | 5.55 | 1.53 | 1.30 | 0.59 | NA |
| -121.86 | 38.04 | Callaway et al. 2012 | 15.30 | 425.00 | Saltmarsh | 1.96 | 165.50 | 5.55 | 1.53 | 1.30 | 0.59 | NA |
| -121.86 | 38.04 | Callaway et al. 2012 | 15.30 | 425.00 | Saltmarsh | 1.96 | 174.10 | 5.55 | 1.53 | 1.30 | 0.59 | NA |
| -121.86 | 38.04 | Callaway et al. 2012 | 15.30 | 425.00 | Saltmarsh | 1.96 | 129.60 | 5.55 | 1.53 | 1.30 | 0.59 | NA |
| -122.50 | 38.01 | Callaway et al. 2012 | 14.40 | 745.00 | Saltmarsh | 1.96 | 186.20 | 21.18 | 1.56 | 1.30 | -1.00 | NA |
| -122.50 | 38.01 | Callaway et al. 2012 | 14.40 | 745.00 | Saltmarsh | 1.96 | 72.90 | 21.18 | 1.56 | 1.30 | -1.00 | NA |
| -122.50 | 38.01 | Callaway et al. 2012 | 14.40 | 745.00 | Saltmarsh | 1.96 | 97.50 | 21.18 | 1.56 | 1.30 | -1.00 | NA |
| -122.50 | 38.01 | Callaway et al. 2012 | 14.40 | 745.00 | Saltmarsh | 1.96 | 86.70 | 21.18 | 1.56 | 1.30 | -1.00 | NA |
| -122.50 | 38.01 | Callaway et al. 2012 | 14.40 | 745.00 | Saltmarsh | 1.96 | 82.10 | 21.18 | 1.56 | 1.30 | -1.00 | NA |
| -122.50 | 38.01 | Callaway et al. 2012 | 14.40 | 745.00 | Saltmarsh | 1.96 | 92.00 | 21.18 | 1.56 | 1.30 | -1.00 | NA |
| -122.30 | 38.22 | Callaway et al. 2012 | 14.70 | 698.00 | Saltmarsh | 1.96 | 47.00 | 12.52 | 1.56 | 1.30 | 0.00 | NA |
| -122.30 | 38.22 | Callaway et al. 2012 | 14.70 | 698.00 | Saltmarsh | 1.96 | 124.70 | 12.52 | 1.56 | 1.30 | 0.00 | NA |
| -122.30 | 38.22 | Callaway et al. 2012 | 14.70 | 698.00 | Saltmarsh | 1.96 | 71.90 | 12.52 | 1.56 | 1.30 | 0.00 | NA |
| -122.30 | 38.22 | Callaway et al. 2012 | 14.70 | 698.00 | Saltmarsh | 1.96 | 72.30 | 12.52 | 1.56 | 1.30 | 0.00 | NA |
| -122.30 | 38.22 | Callaway et al. 2012 | 14.70 | 698.00 | Saltmarsh | 1.96 | 68.20 | 12.52 | 1.56 | 1.30 | 0.00 | NA |
| -122.30 | 38.22 | Callaway et al. 2012 | 14.70 | 698.00 | Saltmarsh | 1.96 | 41.70 | 12.52 | 1.56 | 1.30 | 0.00 | NA |
| -122.30 | 38.22 | Callaway et al. 2012 | 14.70 | 698.00 | Saltmarsh | 1.96 | 232.40 | 12.52 | 1.56 | 1.30 | 0.00 | NA |
| -122.30 | 38.22 | Callaway et al. 2012 | 14.70 | 698.00 | Saltmarsh | 1.96 | 142.60 | 12.52 | 1.56 | 1.30 | 0.00 | NA |
| -122.56 | 38.18 | Callaway et al. 2012 | 14.00 | 807.00 | Saltmarsh | 1.96 | 63.80 | 21.18 | 1.56 | 1.30 | -0.34 | NA |
| -122.56 | 38.18 | Callaway et al. 2012 | 14.00 | 807.00 | Saltmarsh | 1.96 | 78.80 | 21.18 | 1.56 | 1.30 | -0.34 | NA |
| -122.56 | 38.18 | Callaway et al. 2012 | 14.00 | 807.00 | Saltmarsh | 1.96 | 87.70 | 21.18 | 1.56 | 1.30 | -0.34 | NA |
| -122.56 | 38.18 | Callaway et al. 2012 | 14.00 | 807.00 | Saltmarsh | 1.96 | 53.50 | 21.18 | 1.56 | 1.30 | -0.34 | NA |
| -122.56 | 38.18 | Callaway et al. 2012 | 14.00 | 807.00 | Saltmarsh | 1.96 | 53.60 | 21.18 | 1.56 | 1.30 | -0.34 | NA |
| -122.01 | 38.19 | Callaway et al. 2012 | 15.20 | 582.00 | Saltmarsh | 1.96 | 74.90 | 20.66 | 1.55 | 1.30 | 1.57 | NA |
| -122.01 | 38.19 | Callaway et al. 2012 | 15.20 | 582.00 | Saltmarsh | 1.96 | 79.10 | 20.66 | 1.55 | 1.30 | 1.57 | NA |
| -122.01 | 38.19 | Callaway et al. 2012 | 15.20 | 582.00 | Saltmarsh | 1.96 | 118.40 | 20.66 | 1.55 | 1.30 | 1.57 | NA |
| -122.01 | 38.19 | Callaway et al. 2012 | 15.20 | 582.00 | Saltmarsh | 1.96 | 121.50 | 20.66 | 1.55 | 1.30 | 1.57 | NA |
| -122.01 | 38.19 | Callaway et al. 2012 | 15.20 | 582.00 | Saltmarsh | 1.96 | 129.10 | 20.66 | 1.55 | 1.30 | 1.57 | NA |
| -122.01 | 38.19 | Callaway et al. 2012 | 15.20 | 582.00 | Saltmarsh | 1.96 | 106.70 | 20.66 | 1.55 | 1.30 | 1.57 | NA |
| -122.14 | 37.60 | Callaway et al. 2012 | 14.80 | 428.00 | Saltmarsh | 1.96 | 102.50 | 9.52 | 1.50 | 1.25 | 0.00 | NA |
| -122.14 | 37.60 | Callaway et al. 2012 | 14.80 | 428.00 | Saltmarsh | 1.96 | 160.80 | 9.52 | 1.50 | 1.25 | 0.00 | NA |
| -122.14 | 37.60 | Callaway et al. 2012 | 14.80 | 428.00 | Saltmarsh | 1.96 | 132.60 | 9.52 | 1.50 | 1.25 | 0.00 | NA |
| -122.14 | 37.60 | Callaway et al. 2012 | 14.80 | 428.00 | Saltmarsh | 1.96 | 117.90 | 9.52 | 1.50 | 1.25 | 0.00 | NA |
| -76.89 | 37.55 | Campana 1998 | 14.30 | 1108.00 | Saltmarsh | 3.81 | 114.61 | 9.12 | 0.23 | 0.17 | -1.00 | 6.05 |
| -71.33 | 41.73 | Cary et al. 2015 | 9.90 | 1184.00 | Saltmarsh | 2.72 | 133.41 | 1.50 | 0.61 | 0.47 |  | 7.89 |
| -71.45 | 41.69 | Cary et al. 2015 | 9.70 | 1192.00 | Saltmarsh | 2.72 | 237.82 | 1.40 | 0.62 | 0.47 | 2.53 | 7.87 |
| -71.37 | 41.51 | Cary et al. 2015 | 9.90 | 1177.00 | Saltmarsh | 2.72 | 342.23 | 0.79 | 0.65 | 0.47 | 1.81 | 7.95 |
| -71.59 | 41.20 | Cary et al. 2015 | 10.30 | 1100.00 | Saltmarsh | 2.72 | 139.21 | 2.15 | 0.74 | 0.53 | -0.63 | 7.97 |
| -71.32 | 41.63 | Cary et al. 2015 | 10.20 | 1165.00 | Saltmarsh | 2.72 | 168.21 | 0.96 | 0.60 | 0.47 | 2.56 | 7.92 |
| -71.32 | 41.63 | Cary et al. 2015 | 10.20 | 1165.00 | Saltmarsh | 2.72 | 133.41 | 0.96 | 0.60 | 0.47 | 2.56 | 7.92 |
| -75.16 | 38.79 | Chrzastowski et al. 1987 | 13.00 | 1096.00 | Saltmarsh | 3.00 | 267.30 | 17.32 | 1.15 | 0.80 | 0.00 | 7.07 |
| -75.40 | 39.77 | Church 2006 | 12.50 | 1099.00 | Saltmarsh | 2.93 | 197.76 | 11.70 | 1.57 | 1.03 | 0.00 | 5.57 |
| -75.18 | 39.86 | Church 2006 | 12.40 | 1109.00 | Saltmarsh | 2.93 | 487.43 | 4.78 | 1.33 | 1.03 | 1.27 | 5.92 |
| -75.16 | 38.79 | Church et al 1981 | 13.00 | 1096.00 | Saltmarsh | 3.00 | 273.24 | 17.32 | 1.15 | 0.80 | 0.00 | 7.07 |
| -72.95 | 40.71 | Corman et al. 2012 | 11.00 | 1155.00 | Saltmarsh | 3.50 | 121.50 | 6.09 | 0.91 | 0.65 | 0.99 | 7.77 |
| -72.95 | 40.71 | Corman et al. 2012 | 11.00 | 1155.00 | Saltmarsh | 3.50 | 151.80 | 6.09 | 0.91 | 0.65 | 0.99 | 7.77 |
| -81.47 | 31.34 | Craft 2007 | 19.30 | 1292.00 | Saltmarsh | 3.17 | 110.00 | 1.93 | 1.41 | 1.00 | 0.44 | 6.89 |
| -81.40 | 31.32 | Craft 2007 | 19.50 | 1292.00 | Saltmarsh | 3.17 | 145.00 | 1.93 | 1.41 | 1.00 | 0.16 | 7.00 |
| -81.30 | 31.33 | Craft 2007 | 19.40 | 1290.00 | Saltmarsh | 3.17 | 35.00 | 14.33 | 1.40 | 0.99 | 0.19 | 7.17 |
| -81.36 | 31.45 | Craft 2007 | 19.30 | 1292.00 | Saltmarsh | 3.17 | 30.00 | 11.44 | 1.45 | 0.98 | 0.79 | 7.15 |
| -81.32 | 31.42 | Craft 2007 | 19.20 | 1292.00 | Saltmarsh | 3.17 | 40.00 | 11.44 | 1.45 | 0.98 | 0.07 | 7.17 |
| -81.28 | 31.39 | Craft 2007 | 19.20 | 1292.00 | Saltmarsh | 3.17 | 30.00 | 11.44 | 1.45 | 0.98 | 0.46 | 7.20 |
| -81.41 | 31.54 | Craft 2007 | 19.20 | 1278.00 | Saltmarsh | 3.17 | 15.00 | 11.44 | 1.45 | 0.98 | 1.27 | 7.15 |
| -81.30 | 31.55 | Craft 2007 | 19.10 | 1281.00 | Saltmarsh | 3.17 | 25.00 | 11.44 | 1.45 | 0.98 | 0.00 | 7.26 |
| -81.22 | 31.52 | Craft 2007 | 19.10 | 1281.00 | Saltmarsh | 3.17 | 24.00 | 11.44 | 1.45 | 0.98 | -1.00 | 7.30 |
| -81.61 | 31.48 | Craft 2012 | 19.30 | 1289.00 | Saltmarsh | 3.17 | 90.00 | 2.20 | 1.44 | 1.01 | 3.61 | 6.77 |
| -81.29 | 31.98 | Craft 2012 | 19.10 | 1254.00 | Saltmarsh | 3.17 | 83.00 | 11.44 | 1.45 | 0.98 | 3.18 | 7.37 |
| -81.91 | 30.92 | Craft 2012 | 19.70 | 1311.00 | Saltmarsh | 3.17 | 57.00 | 8.07 | 1.44 | 0.96 | 1.94 | 5.47 |
| -81.25 | 31.66 | Craft 2012 | 19.10 | 1281.00 | Saltmarsh | 3.17 | 88.00 | 11.44 | 1.45 | 0.98 | 0.00 | 7.36 |
| -76.73 | 35.37 | Craft et al. 1993 | 16.30 | 1314.00 | Saltmarsh | 1.90 | 106.50 | 1.69 | 0.80 | 0.60 | 0.92 | 9.24 |
| -76.73 | 35.37 | Craft et al. 1993 | 16.30 | 1314.00 | Saltmarsh | 1.90 | 145.50 | 1.69 | 0.80 | 0.60 | 0.92 | 9.24 |
| -75.54 | 35.78 | Craft et al. 1993 | 16.50 | 1322.00 | Saltmarsh | 1.90 | 58.90 | 3.89 | 0.75 | 0.56 | 0.00 | 9.19 |
| -75.54 | 35.78 | Craft et al. 1993 | 16.50 | 1322.00 | Saltmarsh | 1.90 | 21.30 | 3.89 | 0.75 | 0.56 | 0.00 | 9.19 |
| -74.43 | 39.54 | Darke et al 2015 | 11.90 | 1107.00 | Saltmarsh | 0.20 | 152.00 | 7.71 | 0.93 | 0.67 | 0.01 | 7.13 |
| -74.42 | 39.51 | Darke et al 2015 | 11.90 | 1107.00 | Saltmarsh | 0.21 | 118.00 | 7.71 | 0.93 | 0.67 | -0.10 | 7.16 |
| -70.81 | 42.78 | Darke et al 2015 | 10.90 | 1159.00 | Saltmarsh | 0.20 | 116.00 | 2.44 | 4.45 | 2.78 | 0.66 | 7.40 |
| -70.81 | 42.76 | Darke et al 2015 | 10.90 | 1159.00 | Saltmarsh | 0.21 | 170.00 | 2.44 | 4.45 | 2.78 | 0.63 | 7.42 |
| -70.57 | 43.32 | Darke et al 2015 | 8.30 | 1154.00 | Saltmarsh | 0.20 | 41.00 | 1.15 | 4.65 | 2.81 | 1.86 | 6.66 |
| -70.59 | 43.27 | Darke et al 2015 | 8.30 | 1154.00 | Saltmarsh | 0.21 | 95.00 | 1.15 | 4.65 | 2.81 | 2.77 | 6.79 |
| -72.89 | 40.77 | Darke et al 2015 | 11.00 | 1155.00 | Saltmarsh | 0.20 | 93.00 | 4.57 | 0.90 | 0.64 | -1.00 | 7.77 |
| -72.89 | 40.77 | Darke et al 2015 | 11.00 | 1155.00 | Saltmarsh | 0.21 | 114.00 | 4.57 | 0.90 | 0.64 | 0.79 | 7.77 |
| -90.14 | 29.21 | Delaune 1978 | 20.60 | 1544.00 | Saltmarsh | 9.05 | 330.39 | 17.03 | 0.45 | 0.29 | 0.74 | 8.18 |
| -90.14 | 29.21 | Delaune 1978 | 20.60 | 1544.00 | Saltmarsh | 9.05 | 519.42 | 17.03 | 0.45 | 0.29 | 0.74 | 8.18 |
| -90.00 | 29.24 | DeLaune et al 1986 | 20.60 | 1544.00 | Saltmarsh | 9.05 | 198.00 | 17.03 | 0.45 | 0.29 | 1.65 | 8.23 |
| -90.00 | 29.24 | DeLaune et al 1986 | 20.60 | 1544.00 | Saltmarsh | 9.05 | 165.00 | 17.03 | 0.45 | 0.29 | 1.03 | 8.23 |
| -89.93 | 29.28 | DeLaune et al 1986 | 20.60 | 1539.00 | Saltmarsh | 9.05 | 314.34 | 17.03 | 0.45 | 0.29 | 0.03 | 8.24 |
| -89.92 | 29.29 | DeLaune et al 1986 | 20.60 | 1539.00 | Saltmarsh | 9.05 | 135.72 | 17.03 | 0.45 | 0.29 | 0.76 | 8.24 |
| -71.32 | 41.63 | Donnelly and Bertness, 2001 | 10.20 | 1165.00 | Saltmarsh | 2.72 | 144.47 | 0.96 | 0.60 | 0.47 |  | 7.92 |
| -71.32 | 41.63 | Donnelly and Bertness, 2001 | 10.20 | 1165.00 | Saltmarsh | 2.72 | 123.92 | 0.96 | 0.60 | 0.47 |  | 7.92 |
| -71.32 | 41.63 | Donnelly and Bertness, 2001 | 10.20 | 1165.00 | Saltmarsh | 2.72 | 133.45 | 0.96 | 0.60 | 0.47 |  | 7.92 |
| -71.32 | 41.63 | Donnelly and Bertness, 2001 | 10.20 | 1165.00 | Saltmarsh | 2.72 | 95.32 | 0.96 | 0.60 | 0.47 |  | 7.92 |
| -71.30 | 41.72 | Donnelly and Bertness, 2001 | 9.90 | 1184.00 | Saltmarsh | 2.72 | 68.54 | 1.50 | 0.61 | 0.47 | 3.49 | 7.89 |
| -71.30 | 41.72 | Donnelly and Bertness, 2001 | 9.90 | 1184.00 | Saltmarsh | 2.72 | 109.90 | 1.50 | 0.61 | 0.47 | 3.49 | 7.89 |
| -79.17 | 33.49 | Drexler et al. 2013 | 17.60 | 1327.00 | Saltmarsh | 3.90 | 100.00 | 3.43 | 1.10 | 0.77 |  | 8.61 |
| -79.14 | 33.52 | Drexler et al. 2013 | 17.50 | 1310.00 | Saltmarsh | 3.90 | 100.00 | 3.43 | 1.10 | 0.77 |  | 8.64 |
| -79.14 | 33.52 | Drexler et al. 2013 | 17.50 | 1310.00 | Saltmarsh | 3.90 | 100.00 | 3.43 | 1.10 | 0.77 |  | 8.64 |
| -79.16 | 33.52 | Drexler et al. 2013 | 17.50 | 1310.00 | Saltmarsh | 3.90 | 300.00 | 3.43 | 1.10 | 0.77 |  | 8.62 |
| -79.16 | 33.52 | Drexler et al. 2013 | 17.50 | 1310.00 | Saltmarsh | 3.90 | 300.00 | 3.43 | 1.10 | 0.77 |  | 8.62 |
| -79.17 | 33.54 | Drexler et al. 2013 | 17.50 | 1306.00 | Saltmarsh | 3.90 | 50.00 | 3.43 | 1.10 | 0.77 |  | 8.60 |
| -79.17 | 33.54 | Drexler et al. 2013 | 17.50 | 1306.00 | Saltmarsh | 3.90 | 100.00 | 3.43 | 1.10 | 0.77 |  | 8.60 |
| -79.15 | 33.52 | Drexler et al. 2013 | 17.50 | 1310.00 | Saltmarsh | 3.90 | 400.00 | 3.43 | 1.10 | 0.77 |  | 8.63 |
| -79.15 | 33.52 | Drexler et al. 2013 | 17.50 | 1310.00 | Saltmarsh | 3.90 | 300.00 | 3.43 | 1.10 | 0.77 |  | 8.63 |
| -79.16 | 33.52 | Drexler et al. 2013 | 17.50 | 1310.00 | Saltmarsh | 3.90 | 120.00 | 3.43 | 1.10 | 0.77 |  | 8.62 |
| -73.14 | 40.96 | Flessa et al. 1977 | 10.90 | 1159.00 | Saltmarsh | 2.44 | 87.25 | 2.30 | 0.91 | 0.69 | 0.12 | 7.26 |
| -67.15 | 44.85 | Goodman et al. 2007 | 6.20 | 1171.00 | Saltmarsh | 2.40 | 119.47 | 1.20 | 5.63 | 3.44 |  | 5.52 |
| -67.46 | 44.73 | Goodman et al. 2007 | 6.20 | 1222.00 | Saltmarsh | 1.50 | 63.05 | 1.20 | 5.63 | 3.44 | 1.53 | 5.74 |
| -69.61 | 44.04 | Goodman et al. 2007 | 7.50 | 1140.00 | Saltmarsh | 2.90 | 120.15 | 2.75 | 4.54 | 2.79 | -2.00 | 5.64 |
| -67.63 | 44.54 | Goodman et al. 2007 | 6.30 | 1249.00 | Saltmarsh | 2.20 | 135.79 | 1.20 | 5.63 | 3.44 | 1.08 | 5.89 |
| -67.38 | 44.63 | Goodman et al. 2007 | 6.30 | 1242.00 | Saltmarsh | 1.50 | 59.10 | 1.20 | 5.63 | 3.44 | 5.53 | 5.81 |
| -69.57 | 43.94 | Goodman et al. 2007 | 7.70 | 1156.00 | Saltmarsh | 2.80 | 51.69 | 2.75 | 4.54 | 2.79 | 6.89 | 5.94 |
| -69.55 | 44.02 | Goodman et al. 2007 | 7.50 | 1140.00 | Saltmarsh | 2.90 | 137.71 | 1.60 | 4.54 | 2.85 |  | 5.75 |
| -69.57 | 43.98 | Goodman et al. 2007 | 7.70 | 1156.00 | Saltmarsh | 2.90 | 117.28 | 2.75 | 4.54 | 2.79 | 2.50 | 5.84 |
| -70.35 | 43.56 | Goodman et al. 2007 | 7.90 | 1147.00 | Saltmarsh | 2.30 | 112.09 | 1.07 | 4.57 | 2.80 | 0.00 | 6.19 |
| -70.35 | 43.56 | Goodman et al. 2007 | 7.90 | 1147.00 | Saltmarsh | 2.30 | 54.52 | 1.07 | 4.57 | 2.80 | 0.00 | 6.19 |
| -69.98 | 43.87 | Goodman et al. 2007 | 7.60 | 1142.00 | Saltmarsh | 2.30 | 55.09 | 2.75 | 4.54 | 2.79 | 4.78 | 5.69 |
| -76.77 | 37.57 | Greiner and Hershner 1998 | 14.30 | 1110.00 | Saltmarsh | 3.81 | 99.29 | 9.12 | 0.23 | 0.17 |  | 6.15 |
| -76.80 | 37.55 | Greiner and Hershner 1998 | 14.30 | 1110.00 | Saltmarsh | 3.81 | 150.27 | 9.12 | 0.23 | 0.17 | 5.94 | 6.17 |
| -76.80 | 37.55 | Greiner and Hershner 1998 | 14.30 | 1110.00 | Saltmarsh | 3.81 | 142.31 | 9.12 | 0.23 | 0.17 | 1.56 | 6.18 |
| -76.77 | 37.61 | Greiner and Hershner 1998 | 14.30 | 1110.00 | Saltmarsh | 3.81 | 229.65 | 9.12 | 0.23 | 0.17 |  | 6.05 |
| -90.54 | 29.87 | Hatton et al. 1983 | 20.00 | 1608.00 | Saltmarsh | 9.05 | 238.50 | 14.63 | 0.43 | 0.25 | 0.24 | 7.53 |
| -90.54 | 29.87 | Hatton et al. 1983 | 20.00 | 1608.00 | Saltmarsh | 9.05 | 153.00 | 14.63 | 0.43 | 0.25 | 0.24 | 7.53 |
| -90.24 | 29.61 | Hatton et al. 1983 | 20.40 | 1594.00 | Saltmarsh | 9.05 | 134.50 | 17.03 | 0.45 | 0.29 | 0.07 | 8.03 |
| -90.24 | 29.61 | Hatton et al. 1983 | 20.40 | 1594.00 | Saltmarsh | 9.05 | 398.50 | 17.03 | 0.45 | 0.29 | 0.07 | 8.03 |
| -90.17 | 29.34 | Hatton et al. 1983 | 20.50 | 1568.00 | Saltmarsh | 9.05 | 174.00 | 17.03 | 0.45 | 0.29 | 0.28 | 8.15 |
| -90.17 | 29.34 | Hatton et al. 1983 | 20.50 | 1568.00 | Saltmarsh | 9.05 | 413.00 | 17.03 | 0.45 | 0.29 | 0.28 | 8.15 |
| -90.08 | 29.23 | Hatton et al. 1983 | 20.60 | 1544.00 | Saltmarsh | 9.05 | 217.50 | 17.03 | 0.45 | 0.29 | 0.76 | 8.21 |
| -90.08 | 29.23 | Hatton et al. 1983 | 20.60 | 1544.00 | Saltmarsh | 9.05 | 337.50 | 17.03 | 0.45 | 0.29 | 0.76 | 8.21 |
| -75.83 | 37.45 | Kastler and Wiberg 1996 | 14.60 | 1051.00 | Saltmarsh | 4.07 | 55.33 | 8.32 | 0.44 | 0.52 | -1.00 | 7.61 |
| -75.71 | 37.47 | Kastler and Wiberg 1996 | 14.50 | 1046.00 | Saltmarsh | 4.07 | 85.45 | 8.32 | 0.44 | 0.52 | -2.00 | 7.79 |
| -75.83 | 38.23 | Kearney and Stevenson 1991 | 13.50 | 1081.00 | Saltmarsh | 3.74 | 152.15 | 9.98 | 0.54 | 0.43 | -3.00 | 6.85 |
| -75.82 | 38.22 | Kearney and Stevenson 1991 | 13.50 | 1081.00 | Saltmarsh | 3.74 | 263.63 | 9.98 | 0.54 | 0.43 | 0.00 | 6.91 |
| -75.82 | 38.22 | Kearney and Stevenson 1991 | 13.50 | 1081.00 | Saltmarsh | 3.74 | 134.76 | 9.98 | 0.54 | 0.43 | 0.00 | 6.91 |
| -75.85 | 38.23 | Kearney and Stevenson 1991 | 13.70 | 1070.00 | Saltmarsh | 3.74 | 206.13 | 9.98 | 0.54 | 0.43 | 0.75 | 6.81 |
| -75.79 | 38.49 | Kearney and Ward 1986 | 13.40 | 1103.00 | Saltmarsh | 3.69 | 777.00 | 6.34 | 0.61 | 0.50 | 1.38 | 6.52 |
| -76.71 | 38.78 | Khan and Brush 1994 | 13.00 | 1060.00 | Saltmarsh | 3.74 | 250.00 | 5.07 | 0.61 | 0.49 | 0.35 | 3.90 |
| -76.71 | 38.78 | Khan and Brush 1994 | 13.00 | 1060.00 | Saltmarsh | 3.74 | 50.00 | 5.07 | 0.61 | 0.49 | 0.00 | 3.90 |
| -81.10 | 31.87 | Loomis and Craft 2010 | 19.00 | 1259.00 | Saltmarsh | 3.17 | 28.24 | 11.44 | 1.45 | 0.98 | 0.00 | 7.53 |
| -81.15 | 31.91 | Loomis and Craft 2010 | 19.00 | 1259.00 | Saltmarsh | 3.17 | 48.79 | 11.44 | 1.45 | 0.98 | 0.53 | 7.52 |
| -81.26 | 31.92 | Loomis and Craft 2010 | 19.10 | 1254.00 | Saltmarsh | 3.17 | 107.14 | 11.44 | 1.45 | 0.98 | 0.11 | 7.46 |
| -81.34 | 31.35 | Loomis and Craft 2010 | 19.30 | 1292.00 | Saltmarsh | 3.17 | 22.41 | 1.93 | 1.41 | 1.00 | 0.60 | 7.12 |
| -81.40 | 31.30 | Loomis and Craft 2010 | 19.50 | 1292.00 | Saltmarsh | 3.17 | 107.90 | 1.93 | 1.41 | 1.00 | 0.00 | 6.97 |
| -81.51 | 31.35 | Loomis and Craft 2010 | 19.30 | 1289.00 | Saltmarsh | 3.17 | 107.73 | 2.20 | 1.44 | 1.01 | 3.80 | 6.82 |
| -81.51 | 30.99 | Loomis and Craft 2010 | 19.80 | 1292.00 | Saltmarsh | 3.17 | 25.86 | 8.07 | 1.44 | 0.96 | 0.42 | 6.44 |
| -81.57 | 31.00 | Loomis and Craft 2010 | 19.80 | 1292.00 | Saltmarsh | 3.17 | 59.32 | 8.07 | 1.44 | 0.96 | 0.66 | 6.27 |
| -81.75 | 30.98 | Loomis and Craft 2010 | 19.80 | 1304.00 | Saltmarsh | 3.17 | 116.71 | 8.07 | 1.44 | 0.96 | 1.13 | 5.83 |
| -72.86 | 41.26 | McCaffrey and Thomson 1980 | 10.40 | 1130.00 | Saltmarsh | 2.81 | 150.00 | 4.83 | 0.79 | 0.59 | 0.79 | 7.32 |
| -70.51 | 41.55 | Gonneea et al. 2018 | 9.90 | 1178.00 | Saltmarsh | 2.70 | 121.72 | 2.28 | 1.23 | 0.33 | 0.54 | 7.92 |
| -70.51 | 41.55 | Gonneea et al. 2018 | 9.90 | 1178.00 | Saltmarsh | 2.70 | 53.12 | 2.28 | 1.23 | 0.33 | 0.52 | 7.92 |
| -70.51 | 41.55 | Gonneea et al. 2018 | 9.90 | 1178.00 | Saltmarsh | 2.70 | 118.38 | 2.28 | 1.23 | 0.33 | 0.00 | 7.92 |
| -70.58 | 41.56 | Gonneea et al. 2018 | 9.90 | 1178.00 | Saltmarsh | 2.70 | 114.55 | 1.96 | 0.43 | 0.33 | 0.00 | 7.93 |
| -70.58 | 41.56 | Gonneea et al. 2018 | 9.90 | 1178.00 | Saltmarsh | 2.70 | 90.85 | 1.96 | 0.43 | 0.33 | -4.00 | 7.93 |
| -70.58 | 41.56 | Gonneea et al. 2018 | 9.90 | 1178.00 | Saltmarsh | 2.70 | 139.33 | 1.96 | 0.43 | 0.33 | -4.00 | 7.92 |
| -70.50 | 41.58 | Gonneea et al. 2018 | 9.90 | 1178.00 | Saltmarsh | 2.70 | 120.96 | 2.28 | 1.23 | 0.33 | 0.00 | 7.92 |
| -70.50 | 41.58 | Gonneea et al. 2018 | 9.90 | 1178.00 | Saltmarsh | 2.70 | 95.89 | 2.28 | 1.23 | 0.33 | -1.00 | 7.92 |
| -70.50 | 41.58 | Gonneea et al. 2018 | 9.90 | 1178.00 | Saltmarsh | 2.70 | 122.89 | 2.28 | 1.23 | 0.33 | -1.00 | 7.92 |
| -70.54 | 41.56 | Gonneea et al. 2018 | 9.90 | 1178.00 | Saltmarsh | 2.70 | 93.67 | 1.96 | 0.43 | 0.33 | 1.75 | 7.92 |
| -70.54 | 41.56 | Gonneea et al. 2018 | 9.90 | 1178.00 | Saltmarsh | 2.70 | 111.50 | 1.96 | 0.43 | 0.33 | 1.25 | 7.92 |
| -76.89 | 37.55 | Neubauer et al 2002 | 14.30 | 1108.00 | Saltmarsh | 3.81 | 174.49 | 9.12 | 0.23 | 0.17 | -1.00 | 6.05 |
| -81.13 | 32.17 | Noe et al 2016 | 18.80 | 1256.00 | Saltmarsh | 3.17 | 48.00 | 11.20 | 1.36 | 0.97 | 0.30 | 7.34 |
| -79.34 | 33.34 | Noe et al 2016 | 17.70 | 1302.00 | Saltmarsh | 3.17 | 69.00 | 9.25 | 0.99 | 0.71 | 2.01 | 8.48 |
| -90.24 | 29.61 | Nyman et al 1990 | 20.40 | 1594.00 | Saltmarsh | 9.05 | 347.65 | 17.03 | 0.45 | 0.29 | 0.07 | 8.03 |
| -90.54 | 29.87 | Nyman et al 1990 | 20.00 | 1608.00 | Saltmarsh | 9.05 | 81.09 | 14.63 | 0.43 | 0.25 | 0.24 | 7.53 |
| -90.54 | 29.87 | Nyman et al 1990 | 20.00 | 1608.00 | Saltmarsh | 9.05 | 231.05 | 14.63 | 0.43 | 0.25 | 0.24 | 7.53 |
| -90.24 | 29.61 | Nyman et al 1990 | 20.40 | 1594.00 | Saltmarsh | 9.05 | 130.56 | 17.03 | 0.45 | 0.29 | 0.07 | 8.03 |
| -89.92 | 29.29 | Nyman et al 1990 | 20.60 | 1539.00 | Saltmarsh | 9.05 | 380.70 | 17.03 | 0.45 | 0.29 | 0.76 | 8.24 |
| -90.55 | 29.39 | Nyman et al 1993 | 20.40 | 1597.00 | Saltmarsh | 9.05 | 622.50 | 17.03 | 0.45 | 0.29 | 0.00 | 7.87 |
| -90.55 | 29.39 | Nyman et al 1995 | 20.40 | 1597.00 | Saltmarsh | 9.05 | 283.50 | 17.03 | 0.45 | 0.29 | 0.00 | 7.87 |
| -90.55 | 29.39 | Nyman et al 1995 | 20.40 | 1597.00 | Saltmarsh | 9.05 | 220.00 | 17.03 | 0.45 | 0.29 | 0.00 | 7.87 |
| -90.55 | 29.39 | Nyman et al 1995 | 20.40 | 1597.00 | Saltmarsh | 9.05 | 288.00 | 17.03 | 0.45 | 0.29 | 0.00 | 7.87 |
| -90.55 | 29.39 | Nyman et al 1995 | 20.40 | 1597.00 | Saltmarsh | 9.05 | 219.50 | 17.03 | 0.45 | 0.29 | 0.00 | 7.87 |
| -90.55 | 29.39 | Nyman et al 1995 | 20.40 | 1597.00 | Saltmarsh | 9.05 | 155.00 | 17.03 | 0.45 | 0.29 | 0.00 | 7.87 |
| -90.55 | 29.39 | Nyman et al 1995 | 20.40 | 1597.00 | Saltmarsh | 9.05 | 203.00 | 17.03 | 0.45 | 0.29 | 0.00 | 7.87 |
| -90.55 | 29.39 | Nyman et al 1995 | 20.40 | 1597.00 | Saltmarsh | 9.05 | 228.00 | 17.03 | 0.45 | 0.29 | 0.00 | 7.87 |
| -90.55 | 29.39 | Nyman et al 1995 | 20.40 | 1597.00 | Saltmarsh | 9.05 | 388.00 | 17.03 | 0.45 | 0.29 | 0.00 | 7.87 |
| -90.55 | 29.39 | Nyman et al 1995 | 20.40 | 1597.00 | Saltmarsh | 9.05 | 296.00 | 17.03 | 0.45 | 0.29 | 0.00 | 7.87 |
| -90.55 | 29.39 | Nyman et al 1995 | 20.40 | 1597.00 | Saltmarsh | 9.05 | 398.00 | 17.03 | 0.45 | 0.29 | 0.00 | 7.87 |
| -90.55 | 29.39 | Nyman et al 1995 | 20.40 | 1597.00 | Saltmarsh | 9.05 | 217.00 | 17.03 | 0.45 | 0.29 | 0.00 | 7.87 |
| -90.55 | 29.39 | Nyman et al 1995 | 20.40 | 1597.00 | Saltmarsh | 9.05 | 360.00 | 17.03 | 0.45 | 0.29 | 0.00 | 7.87 |
| -90.55 | 29.39 | Nyman et al 1995 | 20.40 | 1597.00 | Saltmarsh | 9.05 | 312.00 | 17.03 | 0.45 | 0.29 | 0.00 | 7.87 |
| -90.55 | 29.39 | Nyman et al 1995 | 20.40 | 1597.00 | Saltmarsh | 9.05 | 172.50 | 17.03 | 0.45 | 0.29 | 0.00 | 7.87 |
| -90.29 | 29.28 | Nyman et al. 2006 | 20.60 | 1550.00 | Saltmarsh | 9.05 | 228.00 | 17.03 | 0.45 | 0.29 | 0.63 | 8.08 |
| -92.95 | 29.90 | Nyman et al. 2006 | 19.90 | 1466.00 | Saltmarsh | 9.05 | 310.50 | 20.75 | 0.65 | 0.44 | 0.02 | 4.83 |
| -90.33 | 29.77 | Nyman et al. 2006 | 20.40 | 1589.00 | Saltmarsh | 9.05 | 308.50 | 14.86 | 0.37 | 0.23 | 0.09 | 7.87 |
| -93.05 | 29.92 | Nyman et al. 2006 | 20.00 | 1446.00 | Saltmarsh | 9.05 | 312.06 | 20.75 | 0.65 | 0.44 | 0.00 | 4.72 |
| -90.54 | 29.41 | Nyman et al. 2006 | 20.40 | 1597.00 | Saltmarsh | 9.05 | 309.00 | 17.03 | 0.45 | 0.29 | -0.35 | 7.87 |
| -91.03 | 29.41 | Nyman et al. 2006 | 20.40 | 1607.00 | Saltmarsh | 9.05 | 212.00 | 17.03 | 0.45 | 0.29 | 0.01 | 7.17 |
| -90.54 | 29.41 | Nyman et al. 2006 | 20.40 | 1597.00 | Saltmarsh | 9.05 | 271.00 | 17.03 | 0.45 | 0.29 | -0.35 | 7.87 |
| -91.03 | 29.41 | Nyman et al. 2006 | 20.40 | 1607.00 | Saltmarsh | 9.05 | 302.00 | 17.03 | 0.45 | 0.29 | 0.01 | 7.17 |
| -71.87 | 41.34 | Orson and Niering1998 | 9.80 | 1212.00 | Saltmarsh | 2.55 | 94.18 | 2.54 | 0.77 | 0.54 | 0.00 | 7.92 |
| -71.86 | 41.33 | Orson and Niering1998 | 9.80 | 1212.00 | Saltmarsh | 2.55 | 164.18 | 2.00 | 0.77 | 0.55 | 2.58 | 7.92 |
| -71.87 | 41.34 | Orson and Niering1998 | 9.80 | 1212.00 | Saltmarsh | 2.55 | 97.04 | 2.54 | 0.77 | 0.54 | 1.23 | 7.92 |
| -75.17 | 39.86 | Orson et al. 1990 | 12.40 | 1109.00 | Saltmarsh | 3.40 | 100.00 | 3.64 | 1.23 | 1.10 | 1.61 | 5.93 |
| -75.17 | 39.86 | Orson et al. 1990 | 12.40 | 1109.00 | Saltmarsh | 3.40 | 110.00 | 3.64 | 1.23 | 1.10 | 2.21 | 5.93 |
| -75.17 | 39.86 | Orson et al. 1990 | 12.40 | 1109.00 | Saltmarsh | 3.40 | 128.00 | 3.64 | 1.23 | 1.10 | 2.21 | 5.93 |
| -75.17 | 39.86 | Orson et al. 1990 | 12.40 | 1109.00 | Saltmarsh | 3.40 | 130.00 | 3.64 | 1.23 | 1.10 | -1.00 | 5.93 |
| -69.95 | 41.83 | Roman et al. 1997 | 9.50 | 1122.00 | Saltmarsh | 2.40 | 256.50 | 2.61 | 3.86 | 2.39 | 0.40 | 7.87 |
| -69.95 | 41.84 | Roman et al. 1997 | 9.50 | 1124.00 | Saltmarsh | 2.40 | 131.10 | 2.61 | 3.86 | 2.39 | -1.00 | 7.87 |
| -69.95 | 41.84 | Roman et al. 1997 | 9.50 | 1124.00 | Saltmarsh | 2.40 | 193.80 | 2.61 | 3.86 | 2.39 | 4.56 | 7.87 |
| -70.84 | 42.73 | Semien 2012 | 9.50 | 1124.00 | Saltmarsh | 2.00 | 73.25 | 2.68 | 4.45 | 2.81 | 0.73 | 7.43 |
| -70.84 | 42.73 | Semien 2012 | 9.50 | 1124.00 | Saltmarsh | 2.00 | 79.48 | 2.68 | 4.45 | 2.81 | 0.73 | 7.43 |
| -70.84 | 42.73 | Semien 2012 | 9.50 | 1124.00 | Saltmarsh | 2.00 | 57.11 | 2.68 | 4.45 | 2.81 | 0.73 | 7.43 |
| -76.06 | 38.42 | Stevenson et al. 1985 | 13.70 | 1089.00 | Saltmarsh | 3.87 | 175.16 | 9.98 | 0.54 | 0.43 | -1.00 | 6.07 |
| -76.06 | 38.42 | Stevenson et al. 1985 | 13.70 | 1089.00 | Saltmarsh | 3.87 | 77.63 | 9.98 | 0.54 | 0.43 | -1.00 | 6.07 |
| -74.27 | 40.57 | Sturdevant et al. 2002 | 11.60 | 1189.00 | Saltmarsh | 4.35 | 160.00 | 1.48 | 1.11 | 0.80 | -3.00 | 6.11 |
| -74.27 | 40.57 | Sturdevant et al. 2002 | 11.60 | 1189.00 | Saltmarsh | 4.35 | 97.50 | 1.48 | 1.11 | 0.80 | 0.45 | 6.11 |
| -74.27 | 40.56 | Sturdevant et al. 2002 | 11.60 | 1189.00 | Saltmarsh | 4.35 | 132.00 | 1.48 | 1.11 | 0.80 | -1.00 | 6.12 |
| -74.27 | 40.56 | Sturdevant et al. 2002 | 11.60 | 1189.00 | Saltmarsh | 4.35 | 142.00 | 1.48 | 1.11 | 0.80 | 0.00 | 6.12 |
| -124.19 | 43.37 | Thom 1992 | 11.10 | 1631.00 | Saltmarsh | 1.70 | 110.15 | 1.68 | 2.04 | 1.67 | 0.19 | NA |
| -124.00 | 46.84 | Thom 1992 | 9.90 | 2052.00 | Saltmarsh | 1.30 | 114.00 | 5.61 | 2.33 | 1.91 | 0.73 | NA |
| -124.00 | 46.84 | Thom 1992 | 9.90 | 2052.00 | Saltmarsh | 1.30 | 119.69 | 5.61 | 2.33 | 1.91 | 0.73 | NA |
| -122.69 | 47.10 | Thom 1992 | 10.40 | 1161.00 | Saltmarsh | 2.00 | 101.53 | 0.56 | 2.42 | 2.08 | 0.00 | NA |
| -122.69 | 47.10 | Thom 1992 | 10.40 | 1161.00 | Saltmarsh | 2.00 | 76.50 | 0.56 | 2.42 | 2.08 | 0.00 | NA |
| -122.69 | 47.10 | Thom 1992 | 10.40 | 1161.00 | Saltmarsh | 2.00 | 104.54 | 0.56 | 2.42 | 2.08 | 0.00 | NA |
| -122.69 | 47.10 | Thom 1992 | 10.40 | 1161.00 | Saltmarsh | 2.00 | 108.00 | 0.56 | 2.42 | 2.08 | 0.00 | NA |
| -122.49 | 48.51 | Thom 1992 | 9.60 | 990.00 | Saltmarsh | 0.80 | 156.81 | 1.99 | 2.61 | 2.07 | 0.00 | NA |
| -124.35 | 47.53 | Thom 1992 | 9.50 | 3087.00 | Saltmarsh | 1.70 | 110.15 | 2.19 | 2.03 | 1.76 | -0.22 | NA |
| -124.35 | 47.53 | Thom 1992 | 9.50 | 3087.00 | Saltmarsh | 1.70 | 100.77 | 2.19 | 2.03 | 1.76 | -0.22 | NA |
| -123.05 | 48.08 | Thom 1992 | 9.20 | 744.00 | Saltmarsh | 0.60 | 110.15 | 0.74 | 2.45 | 2.00 | 0.32 | NA |
| -79.19 | 33.32 | Vogel et al 1996 | 17.60 | 1330.00 | Saltmarsh | 2.00 | 242.60 | 9.25 | 0.99 | 0.71 | -4.00 | 8.64 |
| -79.17 | 33.34 | Vogel et al 1996 | 17.60 | 1327.00 | Saltmarsh | 2.00 | 121.80 | 9.25 | 0.99 | 0.71 | -3.00 | 8.66 |
| -79.20 | 33.32 | Vogel et al 1996 | 17.60 | 1330.00 | Saltmarsh | 2.00 | 205.38 | 9.25 | 0.99 | 0.71 | -3.00 | 8.64 |
| -75.79 | 38.49 | Ward et al 1998 | 13.40 | 1103.00 | Saltmarsh | 3.69 | 175.30 | 6.34 | 0.61 | 0.50 | 1.38 | 6.52 |
| -75.82 | 38.47 | Ward et al 1998 | 13.40 | 1103.00 | Saltmarsh | 3.69 | 175.30 | 6.34 | 0.61 | 0.50 | -1.00 | 6.48 |
| -75.83 | 38.45 | Ward et al 1998 | 13.40 | 1103.00 | Saltmarsh | 3.69 | 285.24 | 9.98 | 0.54 | 0.43 | -1.00 | 6.48 |
| -75.83 | 38.45 | Ward et al 1998 | 13.40 | 1103.00 | Saltmarsh | 3.69 | 285.24 | 9.98 | 0.54 | 0.43 | -1.00 | 6.48 |
| -75.79 | 38.49 | Ward et al 1998 | 13.40 | 1103.00 | Saltmarsh | 3.69 | 495.67 | 6.34 | 0.61 | 0.50 | 1.38 | 6.52 |
| -75.82 | 38.48 | Ward et al 1998 | 13.40 | 1103.00 | Saltmarsh | 3.69 | 495.67 | 6.34 | 0.61 | 0.50 | -1.00 | 6.46 |
| -75.81 | 38.48 | Ward et al 1998 | 13.40 | 1103.00 | Saltmarsh | 3.69 | 143.66 | 6.34 | 0.61 | 0.50 | -1.00 | 6.47 |
| -75.81 | 38.48 | Ward et al 1998 | 13.40 | 1103.00 | Saltmarsh | 3.69 | 143.66 | 6.34 | 0.61 | 0.50 | -1.00 | 6.47 |
| -75.81 | 38.48 | Ward et al 1998 | 13.40 | 1103.00 | Saltmarsh | 3.69 | 163.46 | 6.34 | 0.61 | 0.50 | -1.00 | 6.47 |
| -75.80 | 38.50 | Ward et al 1998 | 13.40 | 1103.00 | Saltmarsh | 3.69 | 685.90 | 6.34 | 0.61 | 0.50 | -1.00 | 6.48 |
| -75.79 | 38.49 | Ward et al 1998 | 13.40 | 1103.00 | Saltmarsh | 3.69 | 181.06 | 6.34 | 0.61 | 0.50 | 1.38 | 6.52 |
| -75.84 | 38.41 | Ward et al 1998 | 13.60 | 1095.00 | Saltmarsh | 3.69 | 181.06 | 9.98 | 0.54 | 0.43 | 0.00 | 6.51 |
| -75.79 | 38.49 | Ward et al 1998 | 13.40 | 1103.00 | Saltmarsh | 3.69 | 115.92 | 6.34 | 0.61 | 0.50 | 1.38 | 6.52 |
| -75.92 | 38.35 | Ward et al 1998 | 13.60 | 1095.00 | Saltmarsh | 3.69 | 115.92 | 9.98 | 0.54 | 0.43 | -2.00 | 6.49 |
| -75.84 | 38.22 | Ward et al 1998 | 13.70 | 1070.00 | Saltmarsh | 3.69 | 39.48 | 9.98 | 0.54 | 0.43 | -2.00 | 6.88 |
| -75.83 | 38.23 | Ward et al 1998 | 13.50 | 1081.00 | Saltmarsh | 3.69 | 198.19 | 9.98 | 0.54 | 0.43 | 0.00 | 6.87 |
| -75.82 | 38.23 | Ward et al 1998 | 13.50 | 1081.00 | Saltmarsh | 3.69 | 6.30 | 9.98 | 0.54 | 0.43 | 0.00 | 6.88 |
| -75.84 | 38.23 | Ward et al 1998 | 13.70 | 1070.00 | Saltmarsh | 3.69 | 43.68 | 9.98 | 0.54 | 0.43 | -2.00 | 6.85 |
| -75.80 | 38.22 | Ward et al 1998 | 13.50 | 1081.00 | Saltmarsh | 3.69 | 110.70 | 9.98 | 0.54 | 0.43 | -1.00 | 6.93 |
| -75.81 | 38.21 | Ward et al 1998 | 13.50 | 1081.00 | Saltmarsh | 3.69 | 83.54 | 9.98 | 0.54 | 0.43 | -2.00 | 6.94 |
| -75.81 | 38.21 | Ward et al 1998 | 13.50 | 1081.00 | Saltmarsh | 3.69 | 137.94 | 9.98 | 0.54 | 0.43 | -3.00 | 6.94 |
| -75.82 | 38.21 | Ward et al 1998 | 13.50 | 1081.00 | Saltmarsh | 3.69 | 79.14 | 9.98 | 0.54 | 0.43 | 0.00 | 6.92 |
| -75.82 | 38.21 | Ward et al 1998 | 13.50 | 1081.00 | Saltmarsh | 3.69 | 65.27 | 9.98 | 0.54 | 0.43 | -3.00 | 6.91 |
| -75.83 | 38.22 | Ward et al 1998 | 13.50 | 1081.00 | Saltmarsh | 3.69 | 95.58 | 9.98 | 0.54 | 0.43 | -2.00 | 6.90 |
| -75.82 | 38.23 | Ward et al 1998 | 13.50 | 1081.00 | Saltmarsh | 3.69 | 47.04 | 9.98 | 0.54 | 0.43 | 0.17 | 6.86 |
| -75.81 | 38.21 | Ward et al 1998 | 13.50 | 1081.00 | Saltmarsh | 3.69 | 126.14 | 9.98 | 0.54 | 0.43 | 2.17 | 6.94 |
| -75.81 | 38.23 | Ward et al 1998 | 13.50 | 1081.00 | Saltmarsh | 3.69 | 130.20 | 9.98 | 0.54 | 0.43 | -4.00 | 6.90 |
| -75.80 | 38.23 | Ward et al 1998 | 13.50 | 1081.00 | Saltmarsh | 3.69 | 301.41 | 9.98 | 0.54 | 0.43 | -2.00 | 6.90 |
| -75.81 | 38.21 | Ward et al 1998 | 13.50 | 1081.00 | Saltmarsh | 3.69 | 136.22 | 9.98 | 0.54 | 0.43 | 2.17 | 6.94 |
| -75.81 | 38.21 | Ward et al 1998 | 13.50 | 1081.00 | Saltmarsh | 3.69 | 188.25 | 9.98 | 0.54 | 0.43 | 0.00 | 6.94 |
| -75.41 | 39.43 | Weston et al. 2014 | 12.60 | 1073.00 | Saltmarsh | 3.40 | 335.80 | 23.64 | 1.54 | 1.03 | -4.00 | 6.16 |
| -75.45 | 39.62 | Weston et al. 2014 | 12.40 | 1103.00 | Saltmarsh | 3.40 | 250.40 | 20.58 | 1.57 | 1.03 | -0.05 | 5.74 |
| -75.36 | 39.79 | Weston et al. 2014 | 12.50 | 1099.00 | Saltmarsh | 3.40 | 109.80 | 9.30 | 1.57 | 1.03 | -3.00 | 5.65 |
| -66.43 | 45.10 | Chmura 2001 | 6.00 | 1239.00 | Saltmarsh | 2.10 | 101.64 | 3.30 | 7.08 | 4.30 | 2.98 | 4.58 |
| 117.60 | 38.83 | Li et al 2003 | 12.30 | 567.00 | Saltmarsh | 3.00 | 34.08 | 21.71 | 1.32 | 1.12 | 0.54 | 1.00 |
| 117.60 | 38.83 | Li et al 2003 | 12.30 | 567.00 | Saltmarsh | 3.00 | 27.77 | 21.71 | 1.32 | 1.12 | 0.54 | 1.00 |
| 117.75 | 38.35 | Li et al 2003 | 12.40 | 577.00 | Saltmarsh | 3.00 | 41.66 | 23.07 | 1.20 | 1.10 | 0.00 | 1.00 |
| 117.75 | 38.35 | Li et al 2003 | 12.40 | 577.00 | Saltmarsh | 3.00 | 27.77 | 23.07 | 1.20 | 1.10 | 0.00 | 1.00 |
| 117.75 | 38.35 | Li et al 2003 | 12.40 | 577.00 | Saltmarsh | 3.00 | 73.22 | 23.07 | 1.20 | 1.10 | 0.00 | 1.00 |
| 117.75 | 38.35 | Li et al 2003 | 12.40 | 577.00 | Saltmarsh | 3.00 | 61.85 | 23.07 | 1.20 | 1.10 | 0.00 | 1.00 |
| 117.75 | 38.35 | Li et al 2003 | 12.40 | 577.00 | Saltmarsh | 3.00 | 53.02 | 23.07 | 1.20 | 1.10 | 0.00 | 1.00 |
| 117.75 | 38.35 | Li et al 2003 | 12.40 | 577.00 | Saltmarsh | 3.00 | 378.70 | 23.07 | 1.20 | 1.10 | 0.00 | 1.00 |
| 117.75 | 38.35 | Li et al 2003 | 12.40 | 577.00 | Saltmarsh | 3.00 | 378.70 | 23.07 | 1.20 | 1.10 | 0.00 | 1.00 |
| 117.56 | 38.67 | Li et al 2003 | 12.30 | 567.00 | Saltmarsh | 3.00 | 161.58 | 22.53 | 1.36 | 1.19 | 0.18 | 1.00 |
| 117.75 | 38.35 | Li et al 2003 | 12.40 | 577.00 | Saltmarsh | 3.00 | 244.89 | 23.07 | 1.20 | 1.10 | 0.00 | 1.00 |
| -66.98 | 45.16 | Chmura et al 2003 | 6.00 | 1171.00 | Saltmarsh | 2.75 | 156.49 | 3.30 | 7.08 | 4.30 | 2.45 | 5.18 |
| -66.43 | 45.10 | Chmura et al 2003 | 6.00 | 1239.00 | Saltmarsh | 2.75 | 481.88 | 3.30 | 7.08 | 4.30 | 2.98 | 4.58 |
| -66.43 | 45.10 | Chmura et al 2003 | 6.00 | 1239.00 | Saltmarsh | 2.75 | 110.85 | 3.30 | 7.08 | 4.30 | 2.98 | 4.58 |
| -66.16 | 45.19 | Chmura et al 2003 | 6.00 | 1291.00 | Saltmarsh | 2.75 | 250.44 | 5.40 | 7.85 | 4.75 | 1.13 | 4.38 |
| -66.16 | 45.19 | Chmura et al 2003 | 6.00 | 1291.00 | Saltmarsh | 2.75 | 169.82 | 5.40 | 7.85 | 4.75 | 1.13 | 4.38 |
| -65.55 | 45.34 | Chmura et al 2003 | 5.40 | 1266.00 | Saltmarsh | 2.75 | 358.02 | 8.98 | 8.23 | 4.99 | 2.54 | 3.98 |
| -65.55 | 45.34 | Chmura et al 2003 | 5.40 | 1266.00 | Saltmarsh | 2.75 | 205.20 | 8.98 | 8.23 | 4.99 | 2.54 | 3.98 |
| -64.79 | 45.62 | Chmura et al 2003 | 5.80 | 1153.00 | Saltmarsh | 2.75 | 743.90 | 16.97 | 8.79 | 5.31 | 3.19 | 3.66 |
| -64.79 | 45.62 | Chmura et al 2003 | 5.80 | 1153.00 | Saltmarsh | 2.75 | 270.96 | 16.97 | 8.79 | 5.31 | 3.19 | 3.66 |
| -64.61 | 45.94 | Chmura et al 2003 | 5.60 | 1141.00 | Saltmarsh | 2.75 | 366.90 | 18.96 | 0.80 | 0.58 | 3.09 | 3.03 |
| -64.61 | 45.94 | Chmura et al 2003 | 5.60 | 1141.00 | Saltmarsh | 2.75 | 631.73 | 18.96 | 0.80 | 0.58 | 3.09 | 3.03 |
| -64.35 | 45.87 | Chmura et al 2003 | 5.50 | 1142.00 | Saltmarsh | 2.75 | 569.39 | 41.09 | 1.26 | 1.44 | 3.88 | 3.21 |
| -64.35 | 45.87 | Chmura et al 2003 | 5.50 | 1142.00 | Saltmarsh | 2.75 | 362.38 | 41.09 | 1.26 | 1.44 | 3.88 | 3.21 |
| 4.27 | 51.12 | Temmerman et al 2003 | 10.30 | 774.00 | Saltmarsh | 2.35 | 168.71 | 7.18 | 3.10 | 2.33 | 1.02 | NA |
| 4.27 | 51.12 | Temmerman et al 2003 | 10.30 | 774.00 | Saltmarsh | 2.35 | 153.38 | 7.18 | 3.10 | 2.33 | 1.02 | NA |
| 4.27 | 51.12 | Temmerman et al 2003 | 10.30 | 774.00 | Saltmarsh | 2.35 | 230.06 | 7.18 | 3.10 | 2.33 | 1.02 | NA |
| 4.27 | 51.12 | Temmerman et al 2003 | 10.30 | 774.00 | Saltmarsh | 2.35 | 61.35 | 7.18 | 3.10 | 2.33 | 0.94 | NA |
| 4.27 | 51.12 | Temmerman et al 2003 | 10.30 | 774.00 | Saltmarsh | 2.35 | 230.06 | 7.18 | 3.10 | 2.33 | 0.94 | NA |
| 4.27 | 51.12 | Temmerman et al 2003 | 10.30 | 774.00 | Saltmarsh | 2.35 | 766.88 | 7.18 | 3.10 | 2.33 | 0.94 | NA |
| 4.27 | 51.12 | Temmerman et al 2003 | 10.30 | 774.00 | Saltmarsh | 2.35 | 306.75 | 7.18 | 3.10 | 2.33 | 1.26 | NA |
| 4.27 | 51.12 | Temmerman et al 2003 | 10.30 | 774.00 | Saltmarsh | 2.35 | 153.38 | 7.18 | 3.10 | 2.33 | 1.26 | NA |
| 4.27 | 51.12 | Temmerman et al 2003 | 10.30 | 774.00 | Saltmarsh | 2.35 | 153.38 | 7.18 | 3.10 | 2.33 | 1.26 | NA |
| 3.72 | 51.35 | Temmerman et al 2003 | 10.10 | 744.00 | Saltmarsh | 2.35 | 23.35 | 16.14 | 4.18 | 2.90 | -1.00 | NA |
| 3.72 | 51.35 | Temmerman et al 2003 | 10.10 | 744.00 | Saltmarsh | 2.35 | 121.42 | 16.14 | 4.18 | 2.90 | -1.00 | NA |
| 3.72 | 51.35 | Temmerman et al 2003 | 10.10 | 744.00 | Saltmarsh | 2.35 | 173.96 | 16.14 | 4.18 | 2.90 | -1.00 | NA |
| 3.72 | 51.35 | Temmerman et al 2003 | 10.10 | 744.00 | Saltmarsh | 2.35 | 96.90 | 16.14 | 4.18 | 2.90 | 0.00 | NA |
| 3.72 | 51.35 | Temmerman et al 2003 | 10.10 | 744.00 | Saltmarsh | 2.35 | 198.48 | 16.14 | 4.18 | 2.90 | 0.00 | NA |
| 3.72 | 51.35 | Temmerman et al 2003 | 10.10 | 744.00 | Saltmarsh | 2.35 | 93.40 | 16.14 | 4.18 | 2.90 | 0.00 | NA |
| 3.72 | 51.35 | Temmerman et al 2003 | 10.10 | 744.00 | Saltmarsh | 2.35 | 467.00 | 16.14 | 4.18 | 2.90 | 0.73 | NA |
| 3.72 | 51.35 | Temmerman et al 2003 | 10.10 | 744.00 | Saltmarsh | 2.35 | 1167.50 | 16.14 | 4.18 | 2.90 | 0.00 | NA |
| 3.72 | 51.35 | Temmerman et al 2003 | 10.10 | 744.00 | Saltmarsh | 2.35 | 60.71 | 16.14 | 4.18 | 2.90 | 0.00 | NA |
| 3.72 | 51.35 | Temmerman et al 2003 | 10.10 | 744.00 | Saltmarsh | 2.35 | 262.69 | 16.14 | 4.18 | 2.90 | 0.00 | NA |
| 3.72 | 51.35 | Temmerman et al 2003 | 10.10 | 744.00 | Saltmarsh | 2.35 | 296.55 | 16.14 | 4.18 | 2.90 | 0.00 | NA |
| -64.92 | 46.77 | Chmura 2004 | 5.10 | 1149.00 | Saltmarsh | 2.30 | 125.99 | 5.40 | 1.52 | 1.18 | 1.48 | 1.86 |
| -64.92 | 46.77 | Chmura 2004 | 5.10 | 1149.00 | Saltmarsh | 2.30 | 126.15 | 5.40 | 1.52 | 1.18 | 1.48 | 1.86 |
| -64.87 | 47.48 | Chmura 2004 | 4.60 | 1089.00 | Saltmarsh | 2.30 | 82.00 | 5.40 | 1.52 | 1.18 | 0.20 | 1.23 |
| -63.43 | 44.66 | Chmura 2004 | 6.40 | 1405.00 | Saltmarsh | 2.40 | 192.60 | 0.35 | 1.24 | 0.80 | 0.13 | 3.17 |
| -63.18 | 44.70 | Chmura 2004 | 6.30 | 1385.00 | Saltmarsh | 2.40 | 131.60 | 0.35 | 1.24 | 0.80 | -1.00 | 3.20 |
| -66.07 | 43.74 | Chmura 2004 | 6.70 | 1257.00 | Saltmarsh | 2.80 | 142.35 | 2.43 | 3.42 | 2.06 | 0.46 | 3.01 |
| -65.81 | 43.67 | Chmura 2004 | 6.40 | 1274.00 | Saltmarsh | 2.80 | 141.40 | 2.43 | 3.42 | 2.06 | 0.65 | 2.98 |
| -65.92 | 43.77 | Chmura 2004 | 6.60 | 1257.00 | Saltmarsh | 2.80 | 94.35 | 2.43 | 3.42 | 2.06 | 0.10 | 3.00 |
| -63.17 | 46.42 | Chmura 2004 | 5.60 | 1111.00 | Saltmarsh | 3.12 | 157.25 | 6.12 | 1.81 | 1.54 | 0.00 | 3.99 |
| -63.17 | 46.42 | Chmura 2004 | 5.60 | 1111.00 | Saltmarsh | 3.12 | 110.00 | 6.12 | 1.81 | 1.54 | 0.00 | 3.99 |
| -63.73 | 46.50 | Chmura 2004 | 5.50 | 1073.00 | Saltmarsh | 3.12 | 86.40 | 9.90 | 1.51 | 1.28 | 1.50 | 2.98 |
| -66.43 | 45.10 | Chmura 2004 | 6.00 | 1239.00 | Saltmarsh | 2.10 | 87.98 | 3.30 | 7.08 | 4.30 | 2.98 | 4.58 |
| -66.36 | 45.12 | Chmura 2004 | 6.00 | 1239.00 | Saltmarsh | 2.10 | 99.12 | 3.30 | 7.08 | 4.30 | 1.21 | 4.53 |
| -66.48 | 45.13 | Chmura 2004 | 6.00 | 1239.00 | Saltmarsh | 2.10 | 109.50 | 3.30 | 7.08 | 4.30 | 2.68 | 4.64 |
| -66.98 | 45.16 | Hung 2006 | 6.00 | 1171.00 | Saltmarsh | 2.75 | 282.15 | 3.30 | 7.08 | 4.30 | 2.45 | 5.18 |
| 151.72 | -32.86 | Howe 2009 | 17.80 | 1119.00 | Saltmarsh | 0.65 | 137.00 | 2.05 | 1.11 | 0.92 | 0.25 | NA |
| 151.71 | -32.84 | Howe 2009 | 17.80 | 1119.00 | Saltmarsh | 0.65 | 64.00 | 2.05 | 1.11 | 0.92 | 0.95 | NA |
| 120.63 | 33.57 | Liu et al 2010 | 14.30 | 988.00 | Saltmarsh | 5.33 | 391.85 | 14.56 | 3.05 | 2.13 | 0.00 | 4.07 |
| 120.62 | 33.57 | Liu et al 2010 | 14.30 | 988.00 | Saltmarsh | 5.33 | 342.87 | 14.56 | 3.05 | 2.13 | -2.00 | 4.06 |
| 120.62 | 33.57 | Liu et al 2010 | 14.30 | 988.00 | Saltmarsh | 5.33 | 238.58 | 14.56 | 3.05 | 2.13 | -1.00 | 4.05 |
| 120.61 | 33.57 | Liu et al 2010 | 14.30 | 988.00 | Saltmarsh | 5.33 | 151.07 | 14.56 | 3.05 | 2.13 | -2.00 | 4.03 |
| 120.57 | 33.56 | Liu et al 2010 | 14.30 | 988.00 | Saltmarsh | 5.33 | 134.93 | 14.56 | 3.05 | 2.13 | 0.00 | 4.05 |
| 120.56 | 33.55 | Liu et al 2010 | 14.30 | 988.00 | Saltmarsh | 5.33 | 95.24 | 14.56 | 3.05 | 2.13 | -1.00 | 4.03 |
| 120.56 | 33.58 | Gao et al 2011 | 14.30 | 988.00 | Saltmarsh | 5.33 | 31.74 | 14.56 | 3.05 | 2.13 | 0.00 | 3.98 |
| 120.56 | 33.58 | Gao et al 2011 | 14.30 | 988.00 | Saltmarsh | 5.33 | 182.48 | 14.56 | 3.05 | 2.13 | 0.00 | 3.98 |
| 120.56 | 33.58 | Gao et al 2011 | 14.30 | 988.00 | Saltmarsh | 5.33 | 231.55 | 14.56 | 3.05 | 2.13 | 0.00 | 3.98 |
| 120.56 | 33.58 | Gao et al 2011 | 14.30 | 988.00 | Saltmarsh | 5.33 | 180.60 | 14.56 | 3.05 | 2.13 | 0.00 | 3.98 |
| 120.56 | 33.58 | Gao et al 2011 | 14.30 | 988.00 | Saltmarsh | 5.33 | 50.40 | 14.56 | 3.05 | 2.13 | 0.00 | 3.98 |
| 120.56 | 33.58 | Gao et al 2011 | 14.30 | 988.00 | Saltmarsh | 5.33 | 65.56 | 14.56 | 3.05 | 2.13 | 0.00 | 3.98 |
| 121.36 | 32.42 | Li et al 2011 | 14.80 | 1026.00 | Saltmarsh | 5.33 | 22.34 | 12.82 | 2.76 | 2.19 | 0.00 | 5.85 |
| 121.36 | 32.42 | Li et al 2011 | 14.80 | 1026.00 | Saltmarsh | 5.33 | 18.11 | 12.82 | 2.76 | 2.19 | 0.00 | 5.85 |
| 119.16 | 37.77 | Wang et al 2014 | 12.20 | 586.00 | Saltmarsh | 3.00 | 201.35 | 30.46 | 0.85 | 0.67 | -1.00 | 1.76 |
| 153.41 | -27.52 | Lovelock et al 2015 | 19.90 | 1463.00 | Saltmarsh | 0.09 | 390.72 | 1.95 | 1.17 | 1.00 | 2.10 | 1.57 |
| 153.44 | -27.44 | Lovelock et al 2015 | 20.00 | 1472.00 | Saltmarsh | 0.09 | 6.53 | 1.95 | 1.17 | 1.00 | 2.68 | 1.49 |
| 153.26 | -27.50 | Lovelock et al 2015 | 19.90 | 1314.00 | Saltmarsh | 0.09 | 1.23 | 3.83 | 1.26 | 1.04 | 2.31 | 1.53 |
| 153.10 | -27.35 | Lovelock et al 2015 | 20.20 | 1516.00 | Saltmarsh | 0.09 | 54.66 | 6.55 | 1.30 | 1.13 | 1.56 | 1.08 |
| 153.04 | -27.30 | Lovelock et al 2015 | 20.30 | 1437.00 | Saltmarsh | 0.09 | 10.08 | 6.55 | 1.30 | 1.13 | 4.94 | 1.04 |
| 9.55 | 53.65 | Butzeck 2015 | 8.40 | 755.00 | Saltmarsh | 2.53 | 170.00 | 6.93 | 3.13 | 2.10 | -2.00 | NA |
| 9.55 | 53.65 | Butzeck 2015 | 8.40 | 755.00 | Saltmarsh | 2.53 | 405.00 | 6.93 | 3.13 | 2.10 | -2.00 | NA |
| 9.03 | 53.90 | Butzeck 2015 | 8.40 | 790.00 | Saltmarsh | 2.53 | 169.00 | 14.78 | 3.09 | 2.10 | -1.00 | NA |
| 9.03 | 53.90 | Butzeck 2015 | 8.40 | 790.00 | Saltmarsh | 2.53 | 810.75 | 14.78 | 3.09 | 2.10 | 0.76 | NA |
| 9.03 | 53.90 | Butzeck 2015 | 8.40 | 790.00 | Saltmarsh | 2.53 | 48.00 | 14.78 | 3.09 | 2.10 | 0.76 | NA |
| 8.88 | 53.97 | Butzeck 2015 | 8.40 | 791.00 | Saltmarsh | 2.53 | 287.00 | 17.60 | 3.37 | 2.02 | 0.00 | NA |
| 120.49 | 33.79 | Bao et al 2015 | 14.20 | 990.00 | Saltmarsh | 5.33 | 165.00 | 24.57 | 1.64 | 1.15 | 0.74 | 3.58 |
| 120.48 | 33.78 | Bao et al 2015 | 14.20 | 990.00 | Saltmarsh | 5.33 | 116.00 | 24.57 | 1.64 | 1.15 | 0.66 | 3.58 |
| -75.10 | 38.40 | Elsey-Quirk et al 2011 | 13.20 | 1092.00 | Saltmarsh | 5.59 | 154.00 | 6.58 | 0.89 | 0.63 | 0.17 | 7.57 |
| -75.10 | 38.40 | Elsey-Quirk et al 2011 | 13.20 | 1092.00 | Saltmarsh | 5.59 | 119.00 | 6.58 | 0.89 | 0.63 | 0.33 | 7.57 |
| 153.13 | -30.30 | Sanders own data | 18.30 | 1790.00 | Saltmarsh | 0.99 | 147.67 | 0.88 | 1.14 | 0.96 | 1.39 | 1.00 |
| 153.13 | -30.30 | Sanders own data | 18.30 | 1790.00 | Saltmarsh | 0.99 | 92.00 | 0.88 | 1.14 | 0.96 | 2.30 | 1.00 |
| 153.12 | -30.30 | Sanders own data | 18.30 | 1790.00 | Saltmarsh | 0.99 | 100.33 | 0.88 | 1.14 | 0.96 | 4.88 | 1.00 |
| 153.23 | -29.98 | Sanders own data | 19.00 | 1514.00 | Saltmarsh | 0.99 | 199.00 | 0.88 | 1.14 | 0.96 | 4.81 | 1.00 |
| 153.22 | -29.97 | Sanders own data | 19.00 | 1514.00 | Saltmarsh | 0.99 | 29.74 | 0.88 | 1.14 | 0.96 | 2.95 | 1.00 |
| 153.21 | -29.98 | Sanders own data | 19.00 | 1514.00 | Saltmarsh | 0.99 | 119.95 | 0.88 | 1.14 | 0.96 | 2.83 | 1.00 |
| 153.26 | -29.88 | Sanders own data | 19.00 | 1514.00 | Saltmarsh | 0.99 | 56.64 | 0.88 | 1.14 | 0.96 |  | 1.62 |
| 153.27 | -29.86 | Sanders own data | 19.00 | 1514.00 | Saltmarsh | 0.99 | 210.16 | 0.88 | 1.14 | 0.96 | 1.55 | 1.50 |
| 153.43 | -29.11 | Sanders own data | 19.40 | 1523.00 | Saltmarsh | 0.99 | 13.82 | 1.14 | 1.13 | 0.97 | 5.88 | 3.25 |
| 153.20 | -30.14 | Sanders own data | 19.70 | 1753.00 | Saltmarsh | 0.99 | 142.43 | 0.88 | 1.14 | 0.96 | 7.08 | 1.00 |
| -125.73 | 49.09 | Chastain et al 2018 | 9.00 | 3126.00 | Saltmarsh | -0.05 | 198.00 | 0.29 | 2.36 | 1.93 | 0.26 | NA |
| -125.73 | 49.09 | Chastain et al 2018 | 9.00 | 3126.00 | Saltmarsh | -0.05 | 37.00 | 0.29 | 2.36 | 1.93 | 0.26 | NA |
| -125.67 | 49.14 | Chastain et al 2018 | 8.70 | 2946.00 | Saltmarsh | -0.05 | 264.00 | 0.29 | 2.36 | 1.93 | 3.41 | NA |
| -125.28 | 49.28 | Chastain et al 2018 | 6.00 | 2298.00 | Saltmarsh | -0.05 | 156.00 | 0.09 | 4.23 | 3.59 |  | NA |
| -125.13 | 49.13 | Chastain et al 2018 | 7.70 | 2393.00 | Saltmarsh | -0.05 | 75.00 | 0.09 | 4.23 | 3.59 | 0.00 | NA |
| 49.57 | 27.29 | Saderne et al. 2018 | 25.00 | 94.00 | Saltmarsh | 1.25 | 9.40 | 10.55 | 1.07 | 0.89 | 0.06 | NA |
| 49.56 | 27.31 | Saderne et al. 2018 | 25.00 | 94.00 | Saltmarsh | 1.25 | 6.00 | 10.55 | 1.07 | 0.89 | -0.07 | NA |
| 49.48 | 27.30 | Saderne et al. 2018 | 25.10 | 91.00 | Saltmarsh | 1.25 | 5.10 | 10.55 | 1.07 | 0.89 | 0.49 | NA |
| 50.02 | 26.74 | Saderne et al. 2018 | 25.50 | 83.00 | Saltmarsh | 1.25 | 3.10 | 10.55 | 1.07 | 0.89 | 0.92 | NA |
| 50.00 | 26.74 | Saderne et al. 2018 | 25.40 | 85.00 | Saltmarsh | 1.25 | 12.00 | 10.55 | 1.07 | 0.89 | 0.00 | NA |
| 50.01 | 26.66 | Saderne et al. 2018 | 25.40 | 85.00 | Saltmarsh | 1.25 | 11.20 | 10.55 | 1.07 | 0.89 | 0.11 | NA |

Table S3. The global extrapolation of tidal wetlands soil C accumulation amount results for each coastal countries.

| Nations | Area (km^2) | | | Current C accumulation Amount (Gg/yr) | | |
| --- | --- | --- | --- | --- | --- | --- |
|  | Tidal marsh | Mangrove | Total | Tidal marsh | Mangrove | Total |
| ABW | 0.00 | 0.99 | 0.99 | 0.00 | 0.12 | 0.12 |
| AGO | 0.00 | 302.12 | 302.12 | 0.00 | 32.86 | 32.86 |
| AIA | 0.00 | 0.05 | 0.05 | 0.00 | 0.01 | 0.01 |
| ALA | 0.82 | 0.00 | 0.82 | 0.26 | 0.00 | 0.26 |
| ALB | 46.62 | 0.00 | 46.62 | 14.94 | 0.00 | 14.94 |
| ARE | 47.79 | 120.51 | 168.30 | 0.99 | 2.44 | 3.42 |
| ARG | 1188.70 | 0.00 | 1188.70 | 382.29 | 0.00 | 382.22 |
| ASM | 0.00 | 0.00 | 0.00 | 0.00 | 0.00 | 0 |
| ATG | 0.00 | 9.25 | 9.25 | 0.00 | 1.16 | 1.16 |
| AUS | 14933.44 | 9552.53 | 24485.96 | 4110.64 | 2751.99 | 6864.3 |
| BEL | 4.76 | 0.00 | 4.76 | 1.21 | 0.00 | 1.21 |
| BEN | 0.00 | 42.88 | 42.88 | 0.00 | 8.94 | 8.94 |
| BES | 0.00 | 2.43 | 2.43 | 0.00 | 0.30 | 0.3 |
| BGD | 0.00 | 4473.66 | 4473.66 | 0.00 | 251.35 | 251.16 |
| BGR | 0.32 | 0.00 | 0.32 | 0.10 | 0.00 | 0.1 |
| BHR | 0.00 | 0.85 | 0.85 | 0.00 | 0.02 | 0.02 |
| BHS | 0.00 | 800.77 | 800.77 | 0.00 | 100.14 | 100.15 |
| BLM | 0.00 | 0.03 | 0.03 | 0.00 | 0.00 | 0 |
| BLZ | 0.00 | 570.74 | 570.74 | 0.00 | 61.02 | 61.02 |
| BMU | 0.00 | 0.10 | 0.10 | 0.00 | 0.02 | 0.02 |
| BRA | 929.87 | 10656.81 | 11586.68 | 299.04 | 2965.70 | 3264.86 |
| BRB | 0.00 | 0.35 | 0.35 | 0.00 | 0.05 | 0.05 |
| BRN | 0.00 | 123.17 | 123.17 | 0.00 | 65.05 | 65.05 |
| CAN | 1927.28 | 0.00 | 1927.28 | 311.18 | 0.00 | 311.18 |
| CHL | 9.24 | 0.00 | 9.24 | 2.97 | 0.00 | 2.97 |
| CHN | 5448.75 | 171.86 | 5620.60 | 1190.09 | 47.61 | 1238.59 |
| CIV | 0.00 | 43.14 | 43.14 | 0.00 | 11.80 | 11.8 |
| CMR | 0.00 | 2229.17 | 2229.17 | 0.00 | 211.72 | 212.08 |
| COD | 0.00 | 218.87 | 218.87 | 0.00 | 23.81 | 23.81 |
| COL | 0.00 | 2127.82 | 2127.82 | 0.00 | 628.88 | 628.47 |
| COM | 0.00 | 1.09 | 1.09 | 0.00 | 0.21 | 0.21 |
| CRI | 0.00 | 390.35 | 390.35 | 0.00 | 89.23 | 89.23 |
| CUB | 0.00 | 4466.20 | 4466.20 | 0.00 | 708.02 | 708.21 |
| CUW | 0.00 | 0.77 | 0.77 | 0.00 | 0.10 | 0.1 |
| CYM | 0.00 | 75.87 | 75.87 | 0.00 | 18.73 | 18.73 |
| CYP | 19.61 | 0.00 | 19.61 | 0.19 | 0.00 | 0.19 |
| DEU | 197.04 | 0.00 | 197.04 | 67.53 | 0.00 | 67.53 |
| DJI | 0.00 | 5.51 | 5.51 | 0.00 | 0.11 | 0.11 |
| DNK | 303.63 | 0.00 | 303.63 | 92.54 | 0.00 | 92.54 |
| DOM | 0.00 | 180.50 | 180.50 | 0.00 | 22.57 | 22.57 |
| ECU | 9.36 | 1396.90 | 1406.25 | 2.98 | 442.12 | 445.33 |
| EGY | 0.00 | 0.34 | 0.34 | 0.00 | 0.00 | 0 |
| ERI | 0.00 | 49.70 | 49.70 | 0.00 | 0.95 | 0.95 |
| ESP | 883.78 | 0.00 | 883.78 | 117.64 | 0.00 | 117.64 |
| EST | 3.89 | 0.00 | 3.89 | 1.25 | 0.00 | 1.25 |
| FIN | 131.93 | 0.00 | 131.93 | 42.29 | 0.00 | 42.29 |
| FJI | 0.00 | 1102.36 | 1102.36 | 0.00 | 62.54 | 62.54 |
| FRA | 800.80 | 0.00 | 800.80 | 120.99 | 0.00 | 120.99 |
| FSM | 0.00 | 88.21 | 88.21 | 0.00 | 24.31 | 24.31 |
| GAB | 480.00 | 1589.25 | 2069.25 | 50.59 | 166.82 | 217.41 |
| GBR | 572.33 | 0.00 | 572.33 | 73.50 | 0.00 | 73.5 |
| GHA | 0.00 | 114.03 | 114.03 | 0.00 | 24.66 | 24.66 |
| GIN | 0.00 | 2382.31 | 2382.31 | 0.00 | 651.61 | 651.61 |
| GLP | 0.00 | 30.14 | 30.14 | 0.00 | 3.77 | 3.77 |
| GMB | 0.00 | 712.38 | 712.38 | 0.00 | 194.85 | 194.85 |
| GNB | 0.00 | 2662.45 | 2662.45 | 0.00 | 728.23 | 728.85 |
| GNQ | 0.00 | 232.57 | 232.57 | 0.00 | 24.38 | 24.38 |
| GRD | 0.00 | 2.12 | 2.12 | 0.00 | 0.32 | 0.32 |
| GTM | 0.00 | 350.69 | 350.69 | 0.00 | 41.62 | 41.62 |
| GUF | 0.00 | 885.10 | 885.10 | 0.00 | 252.67 | 252.3 |
| GUM | 0.08 | 0.32 | 0.40 | 0.02 | 0.09 | 0.11 |
| GUY | 0.00 | 235.25 | 235.25 | 0.00 | 58.72 | 58.72 |
| HKG | 0.01 | 7.55 | 7.56 | 0.00 | 3.64 | 3.65 |
| HND | 0.00 | 676.91 | 676.91 | 0.00 | 117.20 | 117.21 |
| HRV | 5.38 | 0.00 | 5.38 | 1.72 | 0.00 | 1.72 |
| HTI | 0.00 | 146.67 | 146.67 | 0.00 | 18.34 | 18.34 |
| IDN | 0.00 | 27987.28 | 27987.28 | 0.00 | 14709.61 | 14706.87 |
| IND | 0.00 | 4134.28 | 4134.28 | 0.00 | 488.67 | 488.67 |
| IRL | 98.86 | 0.00 | 98.86 | 12.70 | 0.00 | 12.7 |
| IRN | 20.00 | 120.99 | 140.99 | 0.35 | 2.04 | 2.39 |
| ISL | 26.17 | 0.00 | 26.17 | 3.36 | 0.00 | 3.36 |
| ITA | 439.42 | 0.00 | 439.42 | 132.89 | 0.00 | 132.89 |
| JAM | 0.00 | 98.46 | 98.46 | 0.00 | 12.31 | 12.31 |
| JPN | 0.00 | 10.00 | 10.00 | 0.00 | 1.86 | 1.86 |
| KEN | 0.00 | 399.48 | 399.48 | 0.00 | 11.59 | 11.59 |
| KHM | 0.00 | 476.07 | 476.07 | 0.00 | 148.10 | 148.1 |
| KIR | 0.00 | 0.18 | 0.18 | 0.00 | 0.02 | 0.02 |
| KNA | 0.00 | 0.53 | 0.53 | 0.00 | 0.07 | 0.07 |
| LBR | 0.00 | 101.75 | 101.75 | 0.00 | 27.83 | 27.83 |
| LCA | 0.00 | 1.40 | 1.40 | 0.00 | 0.21 | 0.21 |
| LKA | 0.00 | 218.16 | 218.16 | 0.00 | 52.38 | 52.38 |
| LVA | 0.71 | 0.00 | 0.71 | 0.23 | 0.00 | 0.23 |
| MAC | 0.00 | 0.11 | 0.11 | 0.00 | 0.02 | 0.03 |
| MAF | 0.00 | 0.15 | 0.15 | 0.00 | 0.02 | 0.02 |
| MAR | 0.00 | 10.29 | 10.29 | 0.00 | 1.32 | 1.32 |
| MDG | 58.10 | 2412.11 | 2470.21 | 13.91 | 557.04 | 570.95 |
| MDV | 0.00 | 0.85 | 0.85 | 0.00 | 0.20 | 0.2 |
| MEX | 4742.12 | 7323.94 | 12066.06 | 683.04 | 874.41 | 1557.79 |
| MHL | 0.00 | 0.01 | 0.01 | 0.00 | 0.00 | 0 |
| MMR | 0.00 | 5096.77 | 5096.77 | 0.00 | 958.73 | 959.35 |
| MNE | 1.05 | 0.00 | 1.05 | 0.34 | 0.00 | 0.34 |
| MNP | 0.00 | 0.27 | 0.27 | 0.00 | 0.07 | 0.07 |
| MOZ | 0.00 | 2967.18 | 2967.18 | 0.00 | 86.09 | 86.09 |
| MRT | 0.00 | 1.94 | 1.94 | 0.00 | 0.38 | 0.38 |
| MTQ | 0.00 | 10.82 | 10.82 | 0.00 | 1.64 | 1.64 |
| MYS | 0.00 | 5583.57 | 5583.57 | 0.00 | 3247.49 | 3249.08 |
| MYT | 0.00 | 5.16 | 5.16 | 0.00 | 1.17 | 1.17 |
| NCL | 0.00 | 247.67 | 247.67 | 0.00 | 37.03 | 37.03 |
| NGA | 0.00 | 6381.64 | 6381.64 | 0.00 | 1329.96 | 1329.83 |
| NIC | 0.00 | 750.84 | 750.84 | 0.00 | 135.78 | 135.78 |
| NLD | 156.45 | 0.00 | 156.45 | 54.78 | 0.00 | 54.78 |
| NRU | 0.00 | 0.03 | 0.03 | 0.00 | 0.00 | 0 |
| NZL | 196.50 | 272.49 | 469.00 | 15.49 | 24.24 | 39.73 |
| OMN | 0.00 | 2.25 | 2.25 | 0.00 | 0.05 | 0.05 |
| PAK | 0.00 | 588.25 | 588.25 | 0.00 | 101.05 | 101.05 |
| PAN | 0.00 | 1550.44 | 1550.44 | 0.00 | 368.19 | 368.19 |
| PER | 275.24 | 37.23 | 312.47 | 87.72 | 11.87 | 99.59 |
| PHL | 0.00 | 2598.62 | 2598.62 | 0.00 | 1005.64 | 1005.86 |
| PLW | 0.00 | 67.45 | 67.45 | 0.00 | 17.55 | 17.55 |
| PNG | 0.00 | 4870.99 | 4870.99 | 0.00 | 1959.41 | 1962.53 |
| PRI | 58.76 | 83.77 | 142.52 | 7.35 | 10.48 | 17.82 |
| PRT | 198.54 | 0.00 | 198.54 | 25.50 | 0.00 | 25.5 |
| PYF | 0.00 | 0.01 | 0.01 | 0.00 | 0.00 | 0 |
| QAT | 0.00 | 3.85 | 3.85 | 0.00 | 0.08 | 0.08 |
| ROU | 701.73 | 0.00 | 701.73 | 224.94 | 0.00 | 224.54 |
| RUS | 7007.19 | 0.00 | 7007.19 | 648.09 | 0.00 | 648.25 |
| SAU | 0.19 | 81.74 | 81.93 | 0.00 | 1.02 | 1.03 |
| SDN | 0.00 | 2.83 | 2.83 | 0.00 | 0.04 | 0.04 |
| SEN | 0.00 | 1363.40 | 1363.40 | 0.00 | 372.50 | 372.6 |
| SGP | 0.00 | 2.38 | 2.38 | 0.00 | 1.54 | 1.54 |
| SLB | 0.00 | 464.47 | 464.47 | 0.00 | 57.17 | 57.17 |
| SLE | 0.00 | 1462.00 | 1462.00 | 0.00 | 399.89 | 399.49 |
| SLV | 0.00 | 339.24 | 339.24 | 0.00 | 36.83 | 36.83 |
| SOM | 0.00 | 21.34 | 21.34 | 0.00 | 0.59 | 0.59 |
| SUR | 0.00 | 749.03 | 749.03 | 0.00 | 233.59 | 233.71 |
| SVN | 2.21 | 0.00 | 2.21 | 0.71 | 0.00 | 0.71 |
| SWE | 49.86 | 0.00 | 49.86 | 15.97 | 0.00 | 15.97 |
| SYC | 0.00 | 10.94 | 10.94 | 0.00 | 2.49 | 2.49 |
| TCA | 0.00 | 171.88 | 171.88 | 0.00 | 21.49 | 21.49 |
| TGO | 0.00 | 4.02 | 4.02 | 0.00 | 0.84 | 0.84 |
| THA | 0.00 | 2499.15 | 2499.15 | 0.00 | 1116.75 | 1116.41 |
| TLS | 0.00 | 10.18 | 10.18 | 0.00 | 2.91 | 2.91 |
| TON | 0.00 | 7.83 | 7.83 | 0.00 | 0.93 | 0.93 |
| TTO | 0.00 | 63.76 | 63.76 | 0.00 | 15.25 | 15.25 |
| TUR | 256.30 | 0.00 | 256.30 | 34.17 | 0.00 | 34.17 |
| TUV | 0.00 | 0.08 | 0.08 | 0.00 | 0.00 | 0 |
| TWN | 46.26 | 1.65 | 47.91 | 16.15 | 0.60 | 16.75 |
| TZA | 0.00 | 996.67 | 996.67 | 0.00 | 28.92 | 28.92 |
| URY | 24.88 | 0.00 | 24.88 | 8.00 | 0.00 | 8 |
| USA | 19940.71 | 2376.77 | 22317.48 | 3744.31 | 366.71 | 4112.09 |
| VCT | 0.00 | 0.47 | 0.47 | 0.00 | 0.07 | 0.07 |
| VEN | 0.00 | 3348.04 | 3348.04 | 0.00 | 805.14 | 805.3 |
| VGB | 0.00 | 0.78 | 0.78 | 0.00 | 0.10 | 0.1 |
| VIR | 1.27 | 1.86 | 3.13 | 0.16 | 0.23 | 0.39 |
| VNM | 0.05 | 2164.09 | 2164.13 | 0.00 | 543.76 | 543.98 |
| VUT | 0.00 | 13.65 | 13.65 | 0.00 | 0.95 | 0.95 |
| WLF | 0.00 | 0.14 | 0.14 | 0.00 | 0.02 | 0.02 |
| WSM | 0.00 | 3.77 | 3.77 | 0.00 | 0.45 | 0.45 |
| YEM | 0.00 | 10.28 | 10.28 | 0.00 | 0.21 | 0.21 |
| ZAF | 61.47 | 17.48 | 78.96 | 12.08 | 0.51 | 12.59 |
| Total | 62309.45 | 139840.85 | 202150.30 | 12627.21 | 41016.92 | 53650.86 |
